# Lysine acetylation of major *Chlamydia trachom*atis antigens

**DOI:** 10.1016/j.euprot.2016.01.007

**Published:** 2016-01-28

**Authors:** Jelena Mihailovic, Aleksandra Inic-Kanada, Katarina Smiljanic, Elisabeth Stein, Talin Barisani-Asenbauer, Tanja Cirkovic Velickovic

**Affiliations:** a Center of Excellence for Molecular Food Sciences and Department of Biochemistry, University of Belgrade⿿Faculty of Chemistry, Belgrade, Serbia; b OCUVAC⿿Center of Ocular Inflammation and Infection, Laura Bassi Centers of Expertise, Center for Pathophysiology, Infectiology and Immunology, Medical University of Vienna, Vienna, Austria

**Keywords:** *Chlamydia trachomatis*, Lysine acetylation, Antigens, Mass spectrometry

## Abstract

*Chlamydia trachomatis* (Ct) is a human pathogen causing trachoma and infertility. We investigated acetylation at lysine residues of chlamydial antigenic proteins: major outer membrane protein (MOMP), 60 kDa chaperonin (chlamydial Hsp60), elongation factor G (EF-G), enolase and the polymorphic membrane proteins PmpB, PmpE and PmpF. 60 kDa chaperonin, EF-G and PmpB showed the highest degree of acetylation.

Our data show that important Ct antigens could be post-translationally modified by acetylation of lysine residues at multiple sites. Further studies are needed to investigate total acetylome of Ct and the impact PTMs might have on Ct biology and pathogenicity.

## Highlights

⿢ *Chlamydia trachomatis* causes trachoma and sexually transmitted diseases.
⿢ Molecular mechanisms of chlamydial pathogenesis and immunity remain unclear.
⿢ Acetylation of lysine is a post-translational modification that occurs in prokaryotes.
⿢ Lysine acetylation sites were discovered in major chlamydial antigens.
⿢ 60 kDa chaperonin, EF-G and PmpB showed the highest degree of acetylation.

## Graphical abstract

**Figure:**
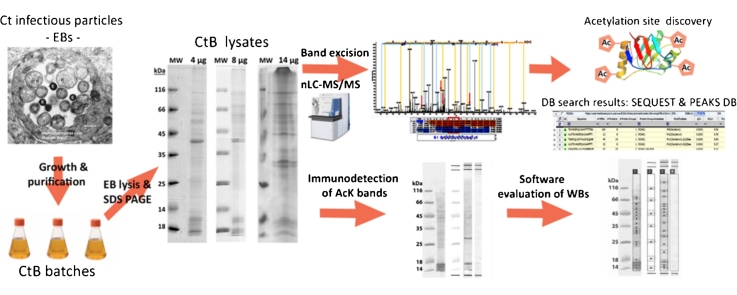

## 1 Introduction

*Chlamydia trachomatis* (Ct) is an obligate intracellular pathogen causing socioeconomically significant morbidities as blindness (trachoma) and female infertility [1]. Ct species is divided into three biovars and various serovars that differ in their major outer membrane proteins and are associated with a number of diseases. Serovars A, B, Ba and C are considered ocular strains, serovars D⿿K cause sexually transmitted diseases worldwide, and serovars L1⿿L3 inflict severe systematic infection lymphogranuloma venereum [2]. Factors that contribute to chlamydial pathogenicity and persistence in tissues are still not fully understood. Proteomic approaches could help elucidate involved mechanisms, since post-translational modifications (PTMs) and level of protein expression are taken into account ⿿ categories otherwise frequently underestimated or overlooked in transcriptomics and genomics studies.

In order for an organism to survive and thrive in ever changing and hostile environments it needs flexible survival mechanisms. PTM of proteins is a strategy that organisms employ to control their biological processes and to adapt to rapid environmental changes. Some of the most studied PTMs include phosphorylation (Ser, Thr, Tyr), ubiquitination and sumoylation (Lys), methylation (Arg, Lys) and acetylation (Lys) [3].

Acetylation of lysine is an important, functional PTM that in eukaryotes, among many significant regulatory mechanisms, also affects cell cycle and tumour suppression through activation of p53 by acetylation of its distinct lysine residues. It occurs on the ε-amino group of lysine, and it is one of the easiest PTM sites to identify, due to the 42 Da mass shift (resulting from the replacement of one of the hydrogens from the amine group with COCH_3_) on the modified amino acid. It is a dynamic process performed by acetyl transferases, while the reverse is performed by deacetylases. Until recently it was thought to occur only in eukaryotes, but emerging data suggest that it occurs in prokaryotes as well [4]. Moreover, this reversible process enables some bacterial species to regulate cellular processes, infect, survive and even thrive in hostile environments [5]. Whether there are post-translationally acetylated lysine Ct antigenic proteins is unknown, as well as their impact and role within pathobiology of Ct.

Lysine acetylation could affect Ct infectious potential and pathogenicity and also alter the immunogenicity of Ct proteins. ⿿If⿿ and ⿿how⿿ need to be answered in order to help reveal the role of specific antigens in immunopathology and protection. Many immunological studies on Ct, mainly on genital and lesser on ocular serovars, have identified several major antigens eliciting strong humoral antibody response with a protective role. These antigens include a serotyping agent like the major outer membrane protein (MOMP) [6], polymorphic membrane proteins (Pmps) and chlamydial Hsp60 (cHsp60; known as GroEL and 60 kDa chaperonin) associated with Ct pathology and other dominant antigens from cytosol with an unknown role, as highly conserved metabolic/modifying and general secretion pathway enzymes [7]. Within this complex story of Ct pathologies, we have recently undertaken a quantitative immunoproteomic and chemometrics study of ocular Ct serovar B (CtB) and its severe trachoma relevant antigen characterization raising IgG response within 2 endemic African country populations at the stage of trachoma-caused blindness (data unpublished). There are no proteomic studies on ocular serovar B and consequently no immunoproteomic research with human trachoma patients and CtB immunodominant antigens, at this point.

Based on this immunoproteomic study, we have selected for our current preliminary research on the existence of Ct proteins⿿ lysine acetylation, 5 most impactful major antigens, that have the highest influence on endemic trachoma patients and healthy control differentiation with significantly higher or exclusive IgG response in trachoma patients elicited by: MOMP, cHSP60), elongation factor G (EF-G), enolase and PmpF. Additionally, lysine acetylation of PmpB as pelvic infection disease dominant antigen [8] and PmpE as promising vaccine candidate for genital disease model [9], were also selected for examination. Out of the seven selected proteins, MOMP, 60 kDa chaperonin and Pmps have been characterized as major (dominant) Ct antigens in all serovars (ocular, genital and LGV) [1].

In this study we assessed three biological samples obtained at different time points of CtB with the gel-aided shotgun proteomics as a model to investigate the acetylation pattern of seven important CtB antigens: MOMP, 60 kDa chaperonin, EF-G, enolase, PmpB, PmpE and PmpF, that has been supported by Western blot of CtB proteome probed with anti-acetylated lysine specific antibody.

## 2 Materials and methods

### 2.1 Ct growth conditions and EBs purification

CtB (ATCC^®^ VR-573⿢) were propagated in McCoy cells (ATCC^®^ CRL-1696⿢, passages varying from P3 to P26) according to standard procedures [10]. Harvested stocks were centrifuged at 200 ÿ *g* to pellet cellular debris. To purify EBs supernatants were layered over discontinuous Renografin gradients as described by Caldwell et al. [11]. The resulting EB fraction was washed twice in 0.01 M sodium phosphate (pH 7.2) containing 0.25 M sucrose and 5 mM l-glutamic acid (SPG). Pellets were resuspended in SPG and stored at ⿿80 °C until analysis. For heat-inactivation samples were incubated at 56 °C for 30 min.

### 2.2 Electrophoresis

SDS-PAGE was carried out according to Laemmli [12] using a Hoefer scientific instrumentation apparatus (Amersham Biosciences, USA) with a discontinuous buffer system. Protein components were resolved on 12% polyacrylamide gels (PAA), which were stained using Coomassie Brilliant Blue R-250 (Sigma⿿Aldrich, Germany).

### 2.3 Western blot

CtB lysate (20 μg/lane) was resolved on 12% PAA and subsequently transferred to a nitrocellulose membrane, 0.2 μm (Bio-Rad, Germany) by blotting according to Towbin et al. [13] using BlueFlash⿢ Semi-Dry Blotter (Serva electrophoresis, Germany). The membrane was blocked with 1% bovine serum albumin (BSA) and probed with commercial anti-acetyl lysine IgG developed in rabbit (Abcam, ab80178) in 1:1000 dilution. BSA solution (0.5%) was used instead of primary antibody as a control. Western blot was developed using alkaline phosphatase conjugated goat anti rabbit IgG (Jackson ImmunoResearch, 111-055-045) in 1:1000 dilution, as a secondary antibody. The phosphatase activity was visualized using 5-bromo-4-chloro-3-indolyl phosphate (BCIP) and nitroblue tetrazolium (NBT). Imaging and analyses of the immunoblot bands were performed by laser Typhoon 7000 series scanner and Image Quant TL 7.0 software (GE Healthcare, USA). The procedure is explained in Supplementary data.

### 2.4 Shotgun proteomics of CTB antigens

#### 2.4.1 Bands excision and preparation for MS

CtB preparations (4 μg, 8 μg and 14 μg) corresponding to 3 biological batches of CtB were electrophoretically resolved on a 12% polyacrylamide gel. After colloidal CBB staining bands from all resolved preparations were excised and in gel digested according to Shevchenko et al. [14]. Briefly, in-gel digestion procedure is compatible with downstream nano-LC⿿MS/MS characterization of digests derived from protein bands. When complex protein mixtures are gel-separated and analyzed by LC⿿MS/MS, the in-gel digestion procedure enables the analysis of entire proteomes of organelles and the majority of proteins in cell lysates. First step is band excision from the gel with a surgical scalpel. After washing with 25 mM ammonium bicarbonate buffer (ABC) and solution of 50% acetonitrile with 25 mM ABC in order to eliminate contaminants related to gel dying, the protein bands were treated with 10 mM DTT to reduce disulphide bridges. Afterwards, bands were alkylated with 55 mM iodoacetamide to block cystein residues and prevent re-formation of disulphide bonds. Proteins in gel bands were digested overnight at 37 °C with 10⿿20 μL of 15 ng/μL trypsin (proteomics grade, Sigma, Germany) applied per band depending on its size (the square area range of the band treated was 5⿿10 mm^2^). Peptide mixtures were filtered by zip tips prior to MS analysis.

#### 2.4.2 Nano-LC⿿MS/MS

All excised and digested bands were analyzed by LC⿿MS/MS. Peptides were chromatographically separated using the EASY-nLC II system (Thermo, Germany) with a 2 column set up: trap column C18-A1, 2 cm (Thermo, Germany) and analytical column PepMap C18, 15 cm ÿ 75 μm, 3 μm particles, 100 ÿ pore size (Thermo, Germany). Mobile phases used were A: 0.1% formic acid in water and B: 0.1% formic acid in acetonitrile. All solvents used were MS grade (Sigma, Germany). Total of 2 μL of each sample was loaded and separated by a gradient over the course of 80 min with a flow rate of 300 nL/min. The flow gradient was (i) 0⿿5 min at 5% B, (ii) 5⿿55 min, 5⿿70% B, (iii) 55⿿60 min 70⿿95% B, (iv) 60⿿70 min 95% B, (v) 70⿿75 min 95⿿5% B, (vi) 75⿿80 min 5% B.

Peptides were analyzed by LTQ Orbitrap XL mass spectrometer in data dependent mode with nano-ESI spray voltage of 1.9 kV, capillary temperature of 275 °C and tube lens value set at 110 V. All spectra were acquired in positive mode with high-resolution full scan in the mass range *m*/*z* 300⿿2000 and Orbitrap resolution of 30,000. The 5 most intense precursors were subjected to collision induced dissociation (CID) with normalized collision energy of 35 and activation time of 30 ms. Dynamic exclusion with 1 repeat count over 10 s and exclusion for 10 s was applied.

#### 2.4.3 Identification of chlamydial proteins through protein database search

Identification of chlamydial proteins was performed by Proteome discoverer 1.3 (Thermo, Germany) and PEAKS Studio 7.5 (Bioinformatics Solutions Inc., Canada). Signature MS/MS spectra were searched by SEQUEST and PEAKS DB algorithms against Uniprot (Swiss-Prot + trEMBL) derived Ct protein database (downloaded on 30/09/2015 from http://www.uniprot.org/). Database contained 40536 sequences, both reviewed and unreviewed. Carbamidomethylation (Cys) as fixed, while acetylation (Lys), oxidation (Met) and deamidation (Gln, Asn) were taken into account as potential modifications, incomplete cleavage (up to 4 missed cleavage sites) and well as peptide FDR of 0.1⿿5% was allowed. Tolerance was set at ±0.8 Da for peptides and at ±10 ppm fragments, ⿿Decoy⿿ was enabled. Spectra acquired from all gel slices were processed as a contiguous input file resulting in a single report file. Only protein hits with SEQUEST score higher than 10, at least 2 high confidence peptides and at least one unique peptide, were searched for lysine acetylation. All MS/MS spectra of Lys-acetylated candidates were manually checked for predicted lysine site(s).

## 3 Results and discussion

### 3.1 Shotgun proteomics of CtB reveals a plethora of protein identities inferred from homology with other Ct serovars

**Fig. 1:**
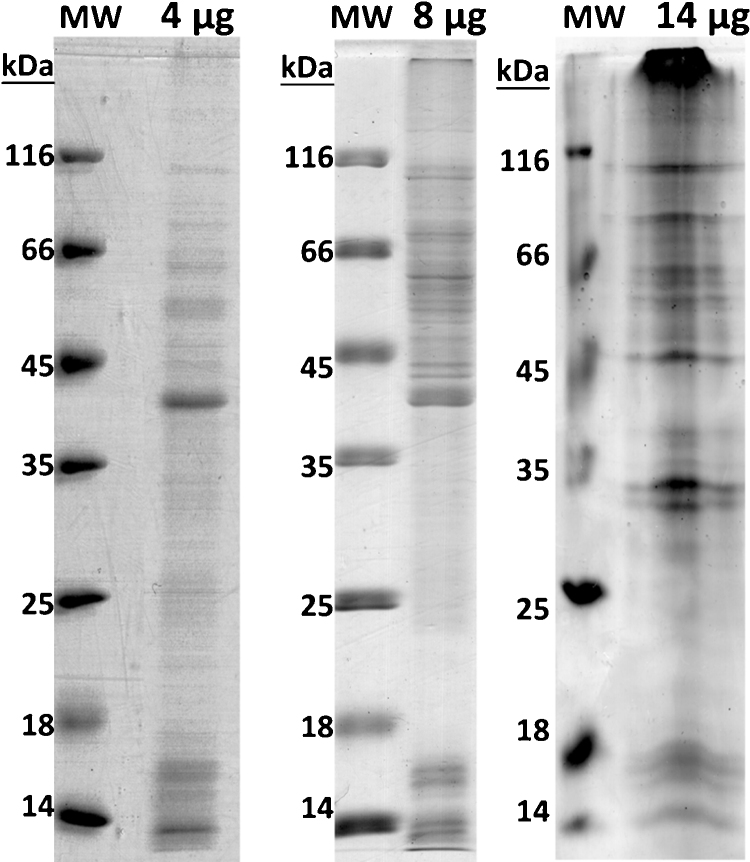
Electrophoretic profile of heat inactivated CtB protein preparations on 12% PAA gel in reducing conditions that were subjected to shotgun proteomics. Each preparation is assigned a label: A-4 μg, B-8 μg, C-14 μg. Abbreviations: MW = molecular weight markers.

CtB grown in McCoy cells (mouse fibroblasts [15]) were harvested between 3rd and 26th passage, EBs were purified and lysates were prepared for protein analysis. Lysates of CtB EB analysed by SDS PAGE showed different protein patterns especially lane C, compared to lanes A and B that share a high degree of similarity (Fig. 1). Dissimilarities in protein profiles, beside different quantities applied, are attributed to variation in protein expression levels depending on cell cycle phase of CtB during purification [16]. All three chlamydial forms (elementary, reticulate and intermediate bodies) are present in different ratios in infected cells [16]. ⿿Contamination⿿ by non-EB proteins could also account for differences in preparations. Contamination due to difficulties in purification of chlamydial proteins is also possible and could be the main reason for differences observed in the profile of lane C. The release of EBs results in rupture of host cells [2] and purifying it from host cellular debris can result in contamination by non-chlamydial proteins, in this case by mouse fibroblast proteins. Indeed, preliminary SwissProt database search (data not shown) on this particular CtB lane revealed minor to moderate presence of mouse fibroblast proteins.

All electrophoretically resolved CtB EB lysates (Fig. 1) were cut into bands (Supplementary data, Fig. E1) and subjected to trypsin digestion prior to mass spectrometry analysis. Upon Uniprot derived Ct database search by SEQUEST, 263 protein hits were matched as chlamydial proteins (Supplementary data, Table E1) with 4 missed cleavages and 5% FDR allowed. PEAKS DB algorithm found 239 hits (Supplementary data, Table E2) under the same conditions. With very strict FDR of 0.1% at peptide level SEQUEST found 125 chlamydial protein hits (Supplementary data, Table E3), while PEAKS DB found 190 hits (Supplementary data, Table E4). We have identified a number of proteins involved in cellular processes ⿿ isomerases, kinases, mutases, chaperone proteins; proteins involved in translation and replication ⿿ 30S and 50S ribosomal proteins, elongation factors (G, P, Ts and Tu), DNA polymerases, helicases and a gyrase; a number of transcription factors and a number of CtB envelope proteins ⿿ MOMP, Pmps and type III secretory system proteins (Supplementary data, Table E1⿿E4). It is interesting to note that great majority of these identifications is based on homology to other serovars as A, L2 and L2b. Only MOMP identification resulted in CtB taxon even though genomes of two strains of CtB were recently sequenced and annotated: strain B/TZ1A828/OT and Jali 20 (http://www.sanger.ac.uk/resources/). The strain we studied is trachoma type B strain HAR-36, and it would be worth undertaking a de novo analysis via PEAKS de novo algorithm to search for sequence differences in homologue proteins between serovars and their possible effects on different aspects of Ct pathobiology.

### 3.2 CtB major trachoma antigens (MOMP, PmpF, cHsp60, EF-G, enolase) and PmpB and PmpE show different lysine acetylation patterns

Out of all identified proteins we have selected 7 proteins to assess for the existence of lysine acetylation PTM within CtB proteome. This selection of lysine acetylated (AcK) CtB candidates, as previously elaborated, was mostly based on the results of our immunoproteomic study (unpublished data) with patients from Sudan and Ethiopia suffering from trachomatous trichiasis and their matched controls. This study revealed 35 major, trachoma relevant antigens (present in more than 50% of patients per group). Out of this antigenic pool influencing diseased and control group segregation, we have selected 5 most impactful major antigens with various functional and localization properties. Additional two proteins (PmpB and PmpE) were selected as major genital Ct antigens [8], [9], [17], since the relevance of Ct acetylated proteins⿿ subject is beyond serovars⿿ differentiation. Beside genomics profiling reports as minor genital antigenic candidate, EF-G [18] and enolase as chlamydial T cell reactive antigen [19], neither has been assigned a major Ct antigen attribute as suggested by our immunoproteomic trachoma study. In addition to being chlamydial antigens, EF-G, enolase and 60 kDa chaperonin are well characterized as acetylated proteins across bacterial species [5].

Proteins we have chosen to investigate for acetylation are either located in the bacterial envelope like MOMP [20] and Pmps [21] or in the cytosol as enolase, EF-G and 60 kDa chaperonin, although enolase could be expressed in the bacterium surface as well [22]. Functionally they cover involvement in housekeeping mechanisms ⿿ enolase [23], protein synthesis ⿿ EF-G [24] or folding⿿60 kDa chaperonine [25]. Pmps are specific outer membrane proteins whose precise functions remain unknown, but they have been implicated in pathogenesis and host cell adherence [17].

No enrichment of acetylated peptides was performed at this stage, so in order to detect as many acetylations as possible (within the chosen 7 proteins) we searched for the PTMs within SEQUEST search results with up to 4 missed cleavages allowed and peptide FDR of 5% (Table 1, Table 2). To confirm the presence of lysine acetylation each MS/MS spectrum was checked for presence of a fragment containing the PTM in question. Every candidate⿿s fragment spectrum was manually curated for lysine acetylation and only those with experimental data confirmation but not theoretical prediction data were accepted as true lysine acetylation sites (representative fragmentation spectra are represented in Supplementary data, Fig. E2⿿E9).

**Table 1:** Sequest identification of 7 acetylated CtB antigens (peptide FDR 5%).

Accession	Protein description	Score	Cov. (%)	# Uniq. Pep.	# Pep.	# PSMs	MW (kDa)	calc. pI	# AC sites	AC positions
P23421	Major outer membrane porin, serovar B OS = *Chlamydia trachomatis* GN = ompA PE = 2 SV = 1 ⿿ [MOMPB_CHLTH]	5588.16	77.92	8	35	3684	42.5	5.34	4	75, 80, 87, 353
Q3KMQ9	60 kDa chaperonin OS = *Chlamydia trachomatis* serovar A (strain ATCC VR-571B/DSM 19440/HAR-13) GN = groL PE = 2 SV = 3 ⿿ [CH60_CHLTA]	2448.06	86.21	30	103	2079	58.1	5.35	13	15, 18, 34, 75, 80, 125, 133, 142, 322, 327, 350, 371, 462
G4NNI1	Elongation factor G OS = *Chlamydia trachomatis* serovar A (strain A2497) GN = fusA PE = 3 SV = 1 ⿿ [G4NNI1_CHLT4]	156.39	79.77	6	74	367	77.4	5.34	12	48, 134, 145, 266, 279, 290, 490, 508, 511, 540, 690, 694
O84591	Enolase OS = *Chlamydia trachomatis* (strain D/UW-3/Cx) GN = eno PE = 3 SV = 1 ⿿ [ENO_CHLTR]	141.08	46.23	6	25	170	45.4	4.73	3	192, 193, 398
Q2TGM5	Polymorphic membrane protein B OS = *Chlamydia trachomatis* GN = pmpB PE = 4 SV = 1 ⿿ [Q2TGM5_CHLTH]	171.94	41.18	7	91	575	182.9	6.02	9	302, 313, 491, 1174, 1175, 1181, 1512, 1518, 1605
Q2TGH5	Polymorphic membrane protein F OS = *Chlamydia trachomatis* GN = pmpF PE = 4 SV = 1 ⿿ [Q2TGH5_CHLTH]	125.85	47.58	6	58	263	112.7	8.43	6	17, 123, 243, 323, 422, 714
Q84FV6	Polymorphic membrane protein E (Fragment) OS = *Chlamydia trachomatis* GN = pmpE PE = 4 SV = 1 ⿿ [Q84FV6_CHLTH]	99.36	40.86	3	39	180	101.7	7.53	6	819, 821, 868, 869, 872, 1003

Abbreviations: Cov.  =  protein sequence coverage, Pep.  =  peptide, # Uniq. Pep.  =  number of unique peptides, AC  =  acetylation, # PSMs  =  number of peptide-spectrum matches.

**Table 2:** Sequest identification of 43 acetylated peptides from 7 CtB antigens (peptide FDR 5%).

Peptide sequence^a^	# PSMs	Modifications	Number of AC sites	οCn	*X*_Corr_	Charge	MH+ [Da]	ο*M* [ppm]	RT [min]	# Missed Cleavage sites	Protein description
VLK*TDVnKEFqMGAKPTTTTGnAVAPSTLTAR	6	K3(Acetyl); N7(Deamidated); Q11(Deamidated); N22(Deamidated)	1	0.000	2.67	4	3392.73	2.21	32.23	2	P23421 Major outer membrane porin, serovar B OS = Chlamydia trachomatis GN = ompA PE = 2 SV = 1 ⿿ [MOMPB_CHLTH]
TDVnK*EFqMGAKPTTTTGNAVAPSTLTAR^b^	71	K5(Acetyl); N4(Deamidated); Q8(Deamidated)	1	0.000	4.01	3	3051.52	8.86	24.12	1	
TDVNKEFQMGAK*PTTTTGnAVAPSTLTAR	5	K12(Acetyl); N19(Deamidated)	1	0.156	1.90	4	3050.51	0.03	31.30	1	
EFQmGAK*PTTTTGNAVAPSTLTAR	3	K7(Acetyl); M4(Oxidation)	1	0.000	1.94	3	2508.24	⿿0.21	24.33	0	
TSAEGQLGDTMQIVSLqLNKMK*SR^b^	3	K22(Acetyl); Q17(Deamidated)	1	0.286	1.82	3	2678.37	9.27	34.18	2	
K*IQK*GVK	3	K1(Acetyl); K4(Acetyl)	2	0.191	1.10	2	884.56	⿿0.87	15.63	2	Q3KMQ9 60 kDa chaperonin OS = Chlamydia trachomatis serovar A (strain ATCC VR-571B/DSM 19440/HAR-13) GN = groL PE = 2 SV = 3 ⿿ [CH60_CHLTA]
VTLGPK*GR	1	K6(Acetyl)	1	0.188	1.08	2	869.52	⿿2.18	27.75	1	
HENmGAQmVK*EVASK*TADK	1	K10(Acetyl); K15(Acetyl); M4(Oxidation); M8(Oxidation);	2	0.290	1.52	3	2190.04	9.85	37.39	2	
GIDKAVK*VVVDqIK^b^	2	K7(Acetyl); Q12(Deamidated)	1	0.175	1.41	2	1554.91	1.17	37.18	2	
AVKVVVDQIK*K	2	K10(Acetyl)	1	0.283	0.43	2	1268.79	⿿3.63	48.10	2	
KISKPVQHHK*	1	K10(Acetyl)	1	0.100	0.45	2	1243.74	6.45	70.29	1	
AKK*VIVSK*	3	K3(Acetyl); K8(Acetyl)	2	0.111	1.12	3	956.61	⿿1.01	22.23	2	
EALEARcESIK*K	14	K11(Acetyl); C7(Carbamidomethyl)	1	0.155	1.42	2	1475.75	⿿3.78	34.00	2	
LAK*LSGGVAVIR	2	K3(Acetyl)	1	0.369	0.53	2	1225.77	2.28	58.62	1	
QIAANAGK*	3	K8(Acetyl)	1	0.435	0.83	2	814.44	⿿0.93	19.21	0	
THK*IGEVHEGGATmDWMEQEQER	3	K3(Acetyl); M14(Oxidation)	1	0.000	0.98	4	2756.18	⿿9.32	19.86	1	G4NNI1 Elongation factor G OS = Chlamydia trachomatis serovar A (strain A2497) GN = fusA PE = 3 SV=1 ⿿ [G4NNI1_CHLT4]
QANK*YGVPR	1	K4(Acetyl)	1	0.179	1.38	3	1074.58	8.57	18.96	1	
IAFVnK*MDR	1	K6(Acetyl); N5(Deamidated)	1	0.062	0.91	3	1136.58	6.19	29.56	1	
KGVIEGK*	2	K7(Acetyl)	1	0.000	1.28	2	772.46	⿿1.38	30.43	1	
NK*GVqqLLDVIVK*WLPSPLDR	3	K2(Acetyl); K13(Acetyl); Q5(Deamidated); Q6(Deamidated)	2	0.360	1.37	3	2504.35	⿿8.95	44.40	2	
VEANVGK*PQVSYK	1	K7(Acetyl)	1	0.161	0.99	2	1460.77	⿿1.81	35.24	0	
TSnSETK*YVK	1	K7(Acetyl); N3(Deamidated)	1	0.230	0.67	2	1199.57	⿿7.04	46.70	1	
YVK*qSGGR	1	K3(Acetyl); Q4(Deamidated)	1	0.000	1.02	2	937.48	1.63	20.35	1	
GnEVVSK*IVGGVIPK ^b^	1	K7(Acetyl); N2(Deamidated)	1	0.110	1.86	2	1538.88	1.29	39.38	1	
ATSTMEPAFFAK*VPqK*IqEEIVK	1	K12(Acetyl); K16(Acetyl); Q15(Deamidated);Q18(Deamidated)	2	0.128	1.23	3	2678.38	7.39	34.41	2	
MGAEVFnALK*K	1	K10(Acetyl); N7(Deamidated)	1	0.312	0.75	2	1250.64	⿿4.25	27.03	1	O84591 Enolase OS = Chlamydia trachomatis (strain D/UW-3/Cx) GN = eno PE = 3 SV = 1 ⿿ [ENO_CHLTR]
mGAEVFNALKK*	5	K11(Acetyl); M1(Oxidation)	1	0.000	1.53	2	1265.64	⿿10.01	47.75	1	
SERIAK*YnR^b^	1	K6(Acetyl); N8(Deamidated)	1	0.210	1.09	2	1179.60	⿿7.31	39.66	2	
ATKDGGAIFA EK*DVSFEnIT SLK*VQTnGAEEK	1	K12(Acetyl); K23(Acetyl); N18(Deamidated); N27(Deamidated)	2	0.248	1.61	3	3483.69	⿿2.70	53.78	3	Q2TGM5 Polymorphic membrane protein B OS = Chlamydia trachomatis GN = pmpB PE = 4 SV = 1 ⿿ [Q2TGM5_CHLTH]
NSSDK*QGGGIYGEDNITLSNLTGKTLFqENTAK	14	K5(Acetyl); Q28(Deamidated)	1	0.000	2.05	3	3543.73	7.21	42.59	2	
GKTISFFDcVHTSTK*K*TGSTQK*VYETLDINK	1	K15(Acetyl); K16(Acetyl); K22(Acetyl); C9(Carbamidomethyl)	3	0.000	1.49	3	3659.82	⿿2.67	39.65	4	
QDFILGAAFSK*MVGKTKAIK*K	2	K11(Acetyl); K20(Acetyl)	2	0.436	1.37	3	2365.34	1.86	41.67	4	
ISMDLK*EPSK^b^	1	K6(Acetyl)	1	0.168	1.74	2	1189.60	⿿9.27	32.75	1	
ESLSnK*ISLTGDTHNLTncYLnNLR	3	K6(Acetyl); N5(Deamidated); N18(Deamidated); C19(Carbamidomethyl); N22(Deamidated)	1	0.328	1.58	4	2922.36	⿿5.13	41.59	1	Q84FV6 Polymorphic membrane protein E (Fragment) OS = Chlamydia trachomatis GN = pmpE PE = 4 SV = 1 ⿿ [Q84FV6_CHLTH]
nPYAAANK*IR	1	K8(Acetyl); N1(Deamidated)	1	0.621	0.77	2	1160.60	⿿4.95	23.62	1	
GNIVFYNnRcFK*	1	K12(Acetyl); N8(Deamidated); C10(Carbamidomethyl)	1	0.310	0.89	3	1574.74	⿿2.04	22.82	1	
GGAIYIDGTSnSK*ISADR^b^	1	K13(Acetyl); N11(Deamidated)	1	0.006	1.65	3	1867.92	7.09	36.69	1	
NLQTK*TPATLTLnHGFLcIEDRAQLAVnR	7	K5(Acetyl); N13(Deamidated); C18(Carbamidomethyl); N28(Deamidated)	1	0.246	2.24	4	3338.75	8.46	37.52	2	
FSQTYTK*LNERYAK	1	K7(Acetyl)	1	0.000	0.67	2	1790.89	⿿7.94	60.57	2	
FLLSFSQSSDKMK*EKETNNR	1	K13(Acetyl)	1	0.316	0.65	2	2431.20	1.46	41.84	3	Q2TGH5 Polymorphic membrane protein F OS = Chlamydia trachomatis GN = pmpF PE = 4 SV = 1 ⿿ [Q2TGH5_CHLTH]
mKEK*ETnnR	2	K4(Acetyl); M1(Oxidation); N7(Deamidated); N8(Deamidated)	1	0.280	0.95	3	1209.54	0.33	12.66	2	
SFYGTK*K*SSK*GK	1	K6(Acetyl); K7(Acetyl); K10(Acetyl)	3	0.069	1.21	2	1485.76	⿿2.11	35.09	3	
GSHSLK*FSHLK^b^	1	K6(Acetyl)	1	0.041	1.42	2	1282.69	⿿0.99	36.13	1	

Abbreviations: AC = acetylation, # PSMs = number of peptide-spectrum matches.

a Acetylated lysine residues are indicated by asterisk (*), while all other AA residue modifications are indicated by lower case letters (m, n, q).

b Spectra figures shown in Supplementary data (Fig. E2⿿E9).

60 kDa chaperonin, EF⿿G and PmpB showed the highest degree of acetylation out of the inspected proteins with 13, 12 and 9 acetylation sites respectively (Table 1). PmpE and PmpF are hexa-acetylated, while MOMP and enolase show only 4 and 3 acetylations, respectively (Table 1).

Observing acetylation patterns at peptide level (Table 2) a total of 2 peptides with 3 acetylated lysines have been detected, which belong to PmpB and PmpF. Out of 43 confirmed acetylated peptides, 7 are diacetylated, while 34 have only one acetylation site (Table 2). Most peptides have a charge of 2 or 3 (22 and 16, respectively), while there are only 5 peptides with a charge of 4 (Table 2).

MOMP is the primary chlamydial serotyping antigen and the most abundant protein in Ct proteome, comprising of up to 30% of its content [11]. This molecule contains antigenic determinants eliciting serovar-, subspecies-, serogroup-, and species-specific antibodies [26], [27]. We have found 4 acetylation sites in MOMP (Table 1, Table 2), and so far there are no published MOMP dynamic acetylation data we could compare our findings to. Worthy to note here is that MOMP represents one of the most impactful CtB antigens responsible for the trachoma patients⿿ differentiation from matched controls according to our unpublished data. Whether MOMP⿿s acetylation plays a role in this remains to be established.

Pmps are chlamydia specific proteins, located in the bacterial envelope involved in chlamydial attachment to human epithelial and endothelial cells. Pmps A⿿I are auto-transporter proteins with an N-terminal part exposed on the surface of the bacteria or secreted, thus being promising vaccine candidates [28]. In connection to that, the polymorphic membrane protein family A⿿I have shown favourable attributes as vaccine components as they are dominant antigenic targets for cellular immune responses [7], [17]. Proteins belonging to this family have been known to undergo infection-dependent post-translational modifications [17]. Similar to MOMP, this is the first study investigating Pmp acetylation. Out of nine members of this family, we have chosen PmpB, PmpE and PmpF for the determination of acetylation sites, and have found 9, 6 and 6 of them respectively. Being at the hotspot of ⿿immunological paradigm⿿ of Ct research and Ct vaccine development, high degree and pattern of Pmps acetylation warrant further, immediate investigation. Recent comprehensive bioinformatic study of Nunes et al. [17] demonstrated that PmpE, PmpG, PmpF and PmpH are the most suitable protective Ct antigenic candidates and that their certain combination in vaccine composition could overcome immune selection of phase variants and low/high polymorphism. PTM such as AcK and in such a high extent as found in PmpE and PmpF, introduces a new level of diversity mimicking ⿿polymorphism⿿ and should be taken into account when examining vaccine immunogenicity, specificity and safety.

Enolase is a well-known, conserved protein, present across prokaryotic and eukaryotic species. It is categorized as a cytosolic metalloenzyme, with a key role in fermentation and glycolysis (catalysis of the reversible conversion of 2-phospho-d-glycerate to phosphoenolpyruvate) [23]. It is one of the most abundantly expressed cytoplasmic proteins in many organisms, being also present on the bacterial surface in many pro- and eukaryotic organisms [22]. Besides enolase⿿s well-studied metabolic function, it moonlights as plasmalogen, fibronectin and laminin binding protein [29] being thus involved in pathogenic and virulence mechanisms [30]. According to published data enolase is not a highly acetylated protein in bacteria, with 1 acetylation site found in *Bacillus subtili*s [31] and *Escherichia coli* [32]. Our study indicates CtB enolase has 3 acetylated lysine residues which is surprising due to the high degree of sequence homology within bacterial enolase isoforms. Sequence similarities between CtB enolase and human counterpart are also relatively high. Aligning identified CtB enolase (Accession: O84591) with human α-enolase isoform (Accession: P06733) in Clustal Omega at www.uniprot.org, showed a sequence similarity of 47% (208 amino acid residues-AA) with additional 123 similar AA positions and identical peptides 11 AA long. This almost definitely precludes enolase as a vaccine candidate, since high sequence homology can be the cause of immunopathology as seen with cHSP60 and Ct infection. Based on this, our finding of triply acetylated enolase speaks in favour of importance of PTM machinery in creating additional diversity.

cHSP60 is a rather controversial antigen, being connected to both, human immunopathology and protection [33], [34]. It has a highly conserved sequence among bacteria and is also involved in human immunopathology in ocular and genital diseases [33], [35]. It was shown by Peeling et al. that cHsp60 is associated with trachomatous scarring in 32% of tested Gambian trachoma patients [36] but there are also studies showing opposite protective effects [37], [38]. Using proteomics tools we have identified 13 acetylation sites which is in accordance with findings for *Thermus thermophilus* (12 sites), *Mycobacterium tuberculosis* and *Pseudomonas aeruginosa* (13 sites each) [5]. Parallel to that, and in accordance with cHSP60 role in both immunopathology and protection, our immunoproteomic data (unpublished), point to cHSP60 being a major antigen that equally well separates all diseased subjects from all healthy controls and all Sudanese from all Ethiopians, being a disease and country specific antigenic marker.

EF-G is a GTP-dependent five domain effector that promotes directional movement of mRNA and tRNAs on the ribosome [39]. EF-G is a well-studied Ct antigen [1] and a universally conserved bacterial GTPase [40]. In our study EF-G stands out as one of the most acetylated proteins, with 12 acetylated lysine residues found. There is a need for further investigation to shed light on this result in connection to the finding that EF-G is a relevant severe trachoma major antigen eliciting solely an IgG response in diseased subjects from both countries and almost none in their matched controls.

### 3.3 Anti-acetyllysine specific antibody immunoblot on CtB proteome reveals many lysine acetylated protein bands and confirms selected trachoma major antigens as acetylated

**Fig. 2:**
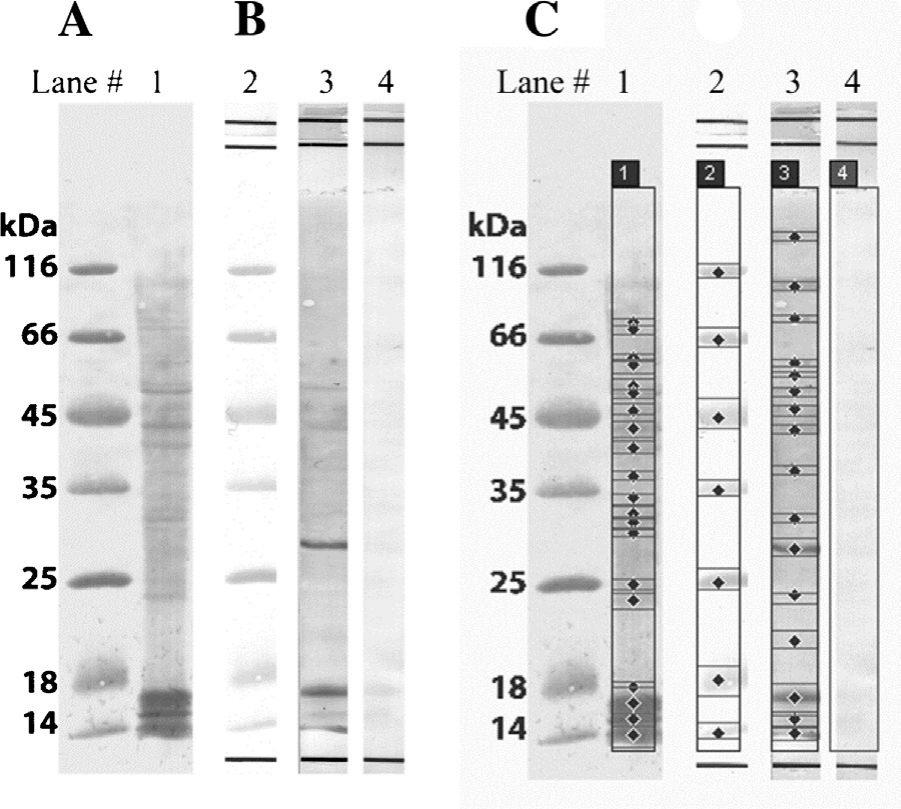
Detection of lysine acetylated proteins from CtB. (A**)** Ponceau S stain of NC membrane showing full CtB lysate; (B) Western blot probed with commercial acetylated lysine-specific primary antibody, and secondary alkaline phosphatase conjugated anti-rabbit antibody. (C.) Acetylated CtB band detection by Image Quant TL software.

Western blot of CtB proteome probed with specific anti-acetylated lysine antibody showed over a dozen of specific lysine acetylated protein bands including also those with molecular masses corresponding to the above mentioned 5 major CtB antigens (Fig. 2 and Supplementary data Fig. E10), with the two most intense bands at 17 and 30 kDa belonging possibly to various ribosomal proteins and urydilate kinase enzyme, as inferred from the immunoproteomics results from our parallel study (unpublished). Immunoblotting studies showed that CtB proteins contain acetylated lysines when grown in McCoy cells (Fig. 2 and Supplementary data Fig. E10) and also demonstrated specificity, since there are no corresponding bands in conjugate controls as analyzed by Image Quant TL software. Sixteen acetylated proteins were detected (Fig. 2. and Supplementary data Fig. E10) that ranged in size from 14 kDa to 130 kDa. This finding besides its confirmatory role for the big part of selected AcK protein candidates, also demonstrates that there are far more acetylated CtB proteins than our initial selection. Regarding the PmpB and PmpE that were not recognized as trachoma relevant antigens in our immunoproteomic study (unpublished) we could not observe their full length protein bands as acetylated and there is a possibility that they are present as fragments as there are faint bands at approximately 130 and 80 kDa [8].

Besides major bands, in anti-acetylated lysine probed blot, there are also many weakly recognized bands showing diversity of lysine acetylation that were below Western blot limit of detection. Both approaches applied in our study aim at detecting the most abundant as well as the most prominently acetylated proteins of CtB. On the other hand, due to regulatory role of lysine acetylation, many proteins undergo this PTM in various stages of bacterium⿿s life cycle. Therefore, for an in depth study of the total CtB acetylation pattern, additional experiments involving enrichment of the acetylated fraction with anti-acetylated lysine antibodies are warranted.

## 4 Conclusions

Here we present for the first time that lysine acetylation is frequent in important CtB antigens. Acetylation of lysine residues could alter the conformation as well as the immunogenicity of antigens, which is of the utmost importance for further understanding of chlamydial biology and vaccine development. It can also be of importance in clarifying yet unresolved mechanisms of Ct pathogenicity. Our data suggest that important Ct antigens could be post-translationally modified by acetylation of lysine residues at multiple sites. For a better understanding of Ct pathology and selection of suitable antigen candidates for Ct vaccine development, total acetylome of Ct should be further investigated by MS-based strategies that also involve enrichment of modified proteins by immunoprecipitation.

## Conflict of interest

The authors declare no conflict of interest.
