# Supplementary material for: Lysine acetylation of major Chlamydia trachomatis antigens
Source: EuPA Open Proteom. 2016 Jan 28;10:63–9. doi: 10.1016/j.euprot.2016.01.007 (PMC5988610; doi:10.1016/j.euprot.2016.01.007)
Supplement: Supplementary file 1 [file mmc1.docx]

# Supplementary data

## Lysine acetylation of major *Chlamydia trachom*atis antigens

Jelena Mihailovic^1*^, Aleksandra Inic-Kanada^2*^, Katarina Smiljanic^1^, Elisabeth Stein^2^, Talin Barisani-Asenbauer^2**^, Tanja Cirkovic Velickovic^1**^

^1^ Center of Excellence for Molecular Food Sciences, University of Belgrade - Faculty of Chemistry, Belgrade, Serbia

^2^ OCUVAC – Center of Ocular Inflammation and Infection; Laura Bassi Centers of Expertise; Center for Pathophysiology, Infectology and Immunology; Medical University of Vienna; Vienna, Austria

## Materials and methods

### Evaluation of  CtB Lysine acetylation westernblot

Imaging and analyses of the westernblot probed with AcK specific antibody was performed by laser Typhoon 7000 series scanner and Image Quant TL 7.0 software (GE Healthcare, USA). In brief, for scanning the BCIP/NBT developed immunomembranes, Typhoon’s fluorescent stage and in-lab designed scanning membrane program were applied according to manufacturer’s general directions. To quantify intensity of the immunologic responses, an un-calibrated mode of pixel inverter function was chosen (linear function defined by Inverted pixel value = Bit-depth-1-pixel value, with Bit-depth being 255 for 8–bit grey scale images), with 16 pixel fixed width band detection, peak slope of 150, noise reduction level 7, minimal size of peak detected at 3% and rolling disc radius of 200 for background subtraction.

### Figures and tables

**Fig. E1**. Excision maps of heat inactivated CtB protein preparations on 4-12% PAA gel in reducing conditions that were subjected to shotgun proteomics. Each preparation is assigned a label: A – 4 µg, B – 8 µg, C – 14 µg. Abbreviations: MW = molecular weight markers [Figure is single column wide, dimensions 9 cm x 10.5cm, 500ppi]


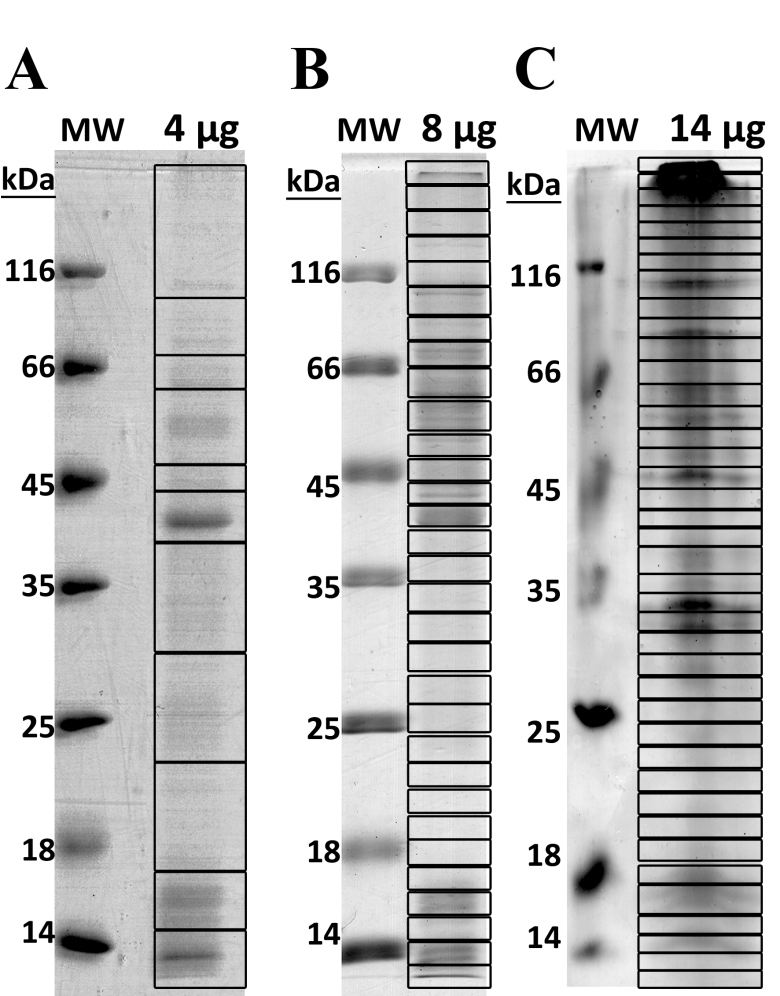


**Fig. E2.** MS/MS spectrum of Major outer membrane porin (P23421) acetylated peptide TDVnK*EFqMGAKPTTTTGNAVAPSTLTAR

[Figure is double column wide, dimensions 19 cm x 9.5 cm, 500ppi]


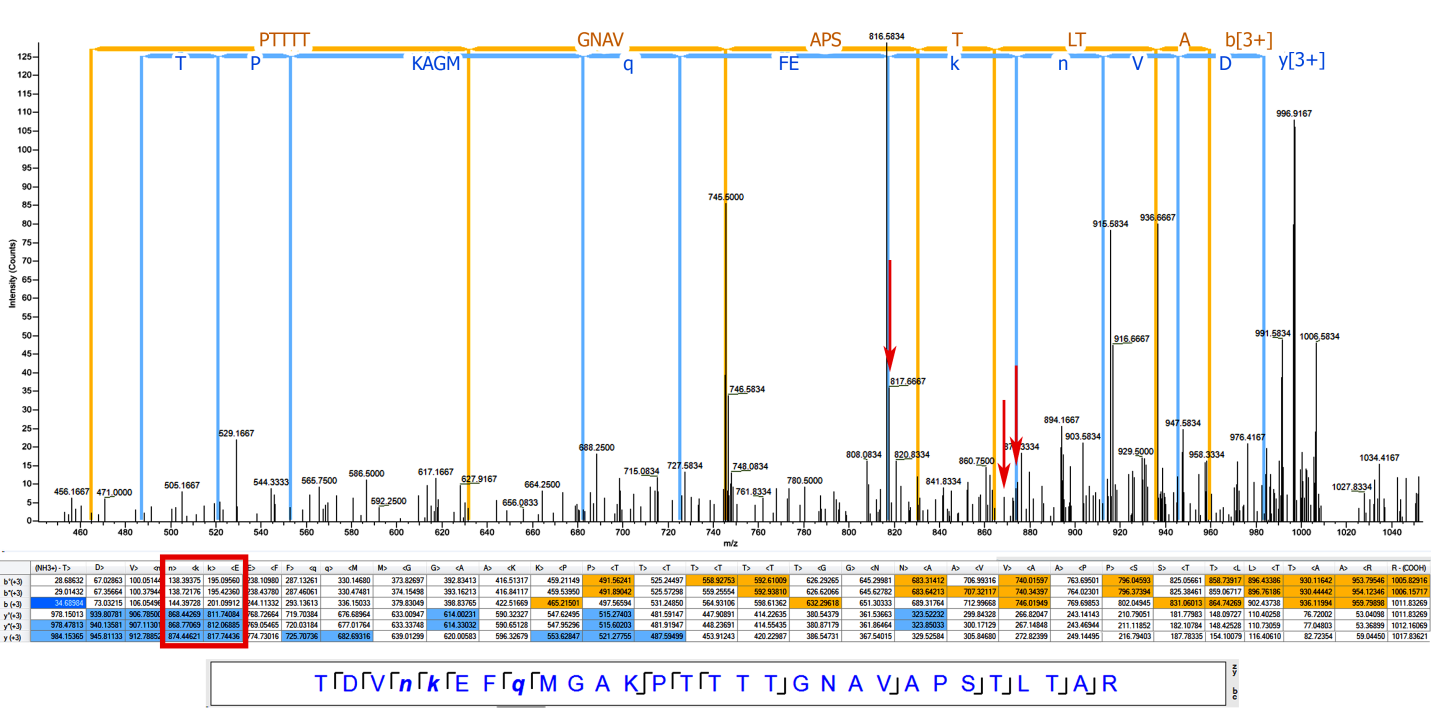


**Fig. E3.** MS/MS spectrum of Major outer membrane porin (P23421) acetylated peptide TSAEGQLGDTMQIVSLqLNKMK*SR

[Figure is double column wide, dimensions 19 cm x 10 cm, 500ppi]


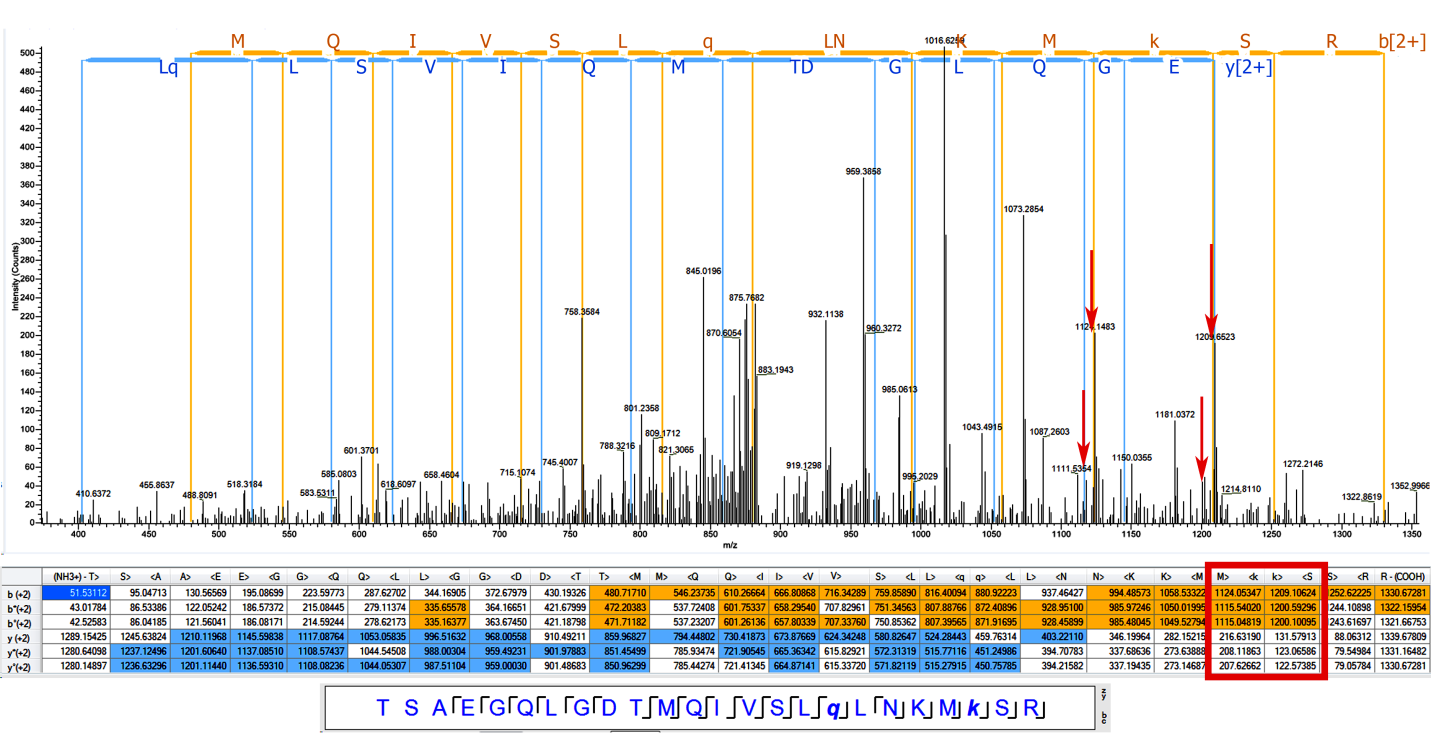


**Fig. E4.** MS/MS spectrum of 60 kDa chaperonin (Q3KMQ9) acetylated peptide GIDKAVK*VVVDqIK

[Figure is double column wide, dimensions 19 cm x 9.1 cm, 500ppi]


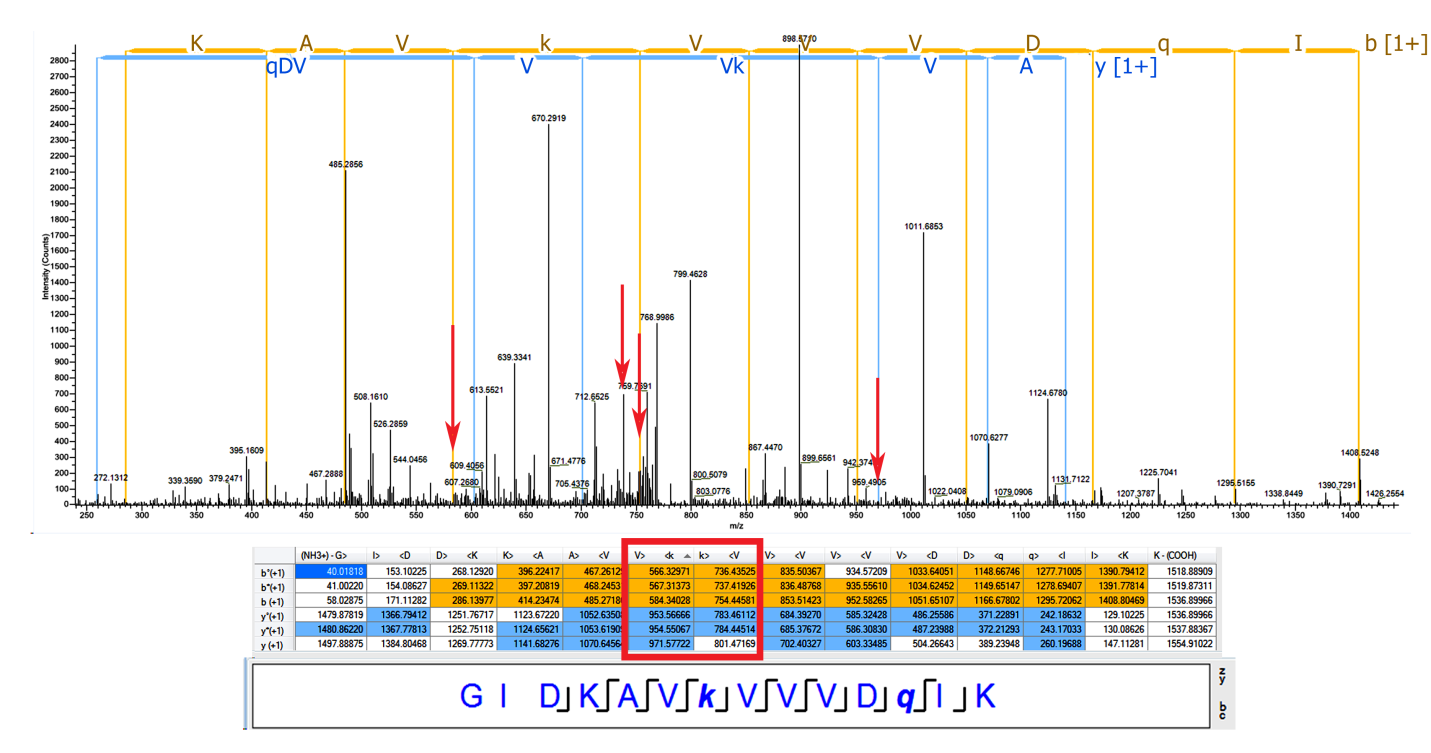


**Fig. E5.** MS/MS spectrum of Elongation factor G (G4NNI1) acetylated peptide GnEVVSK*IVGGVIPK

[Figure is double column wide, dimensions 19 cm x 9 cm, 500ppi]


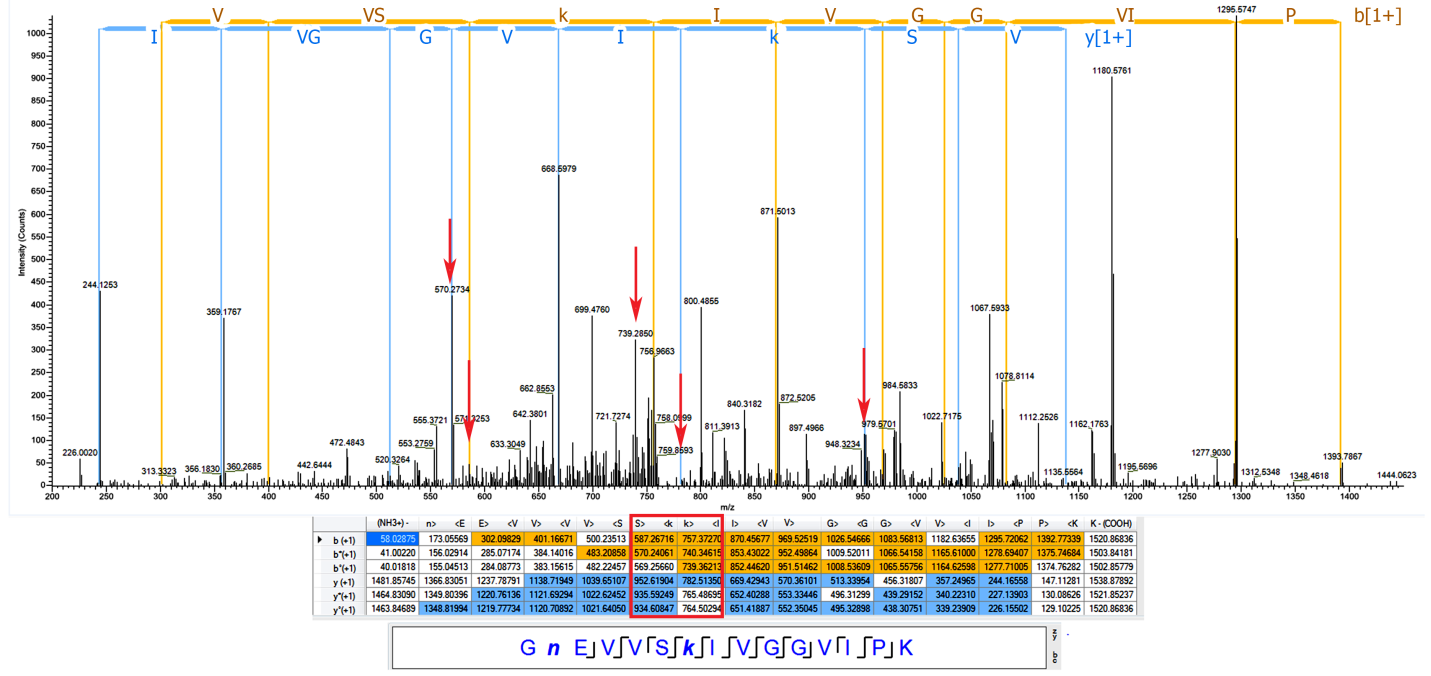


**Fig. E6.** MS/MS spectrum of Enolase (O84591) acetylated peptide SERIAK*YnR

[Figure is double column wide, dimensions 19 cm x 9 cm, 500ppi]


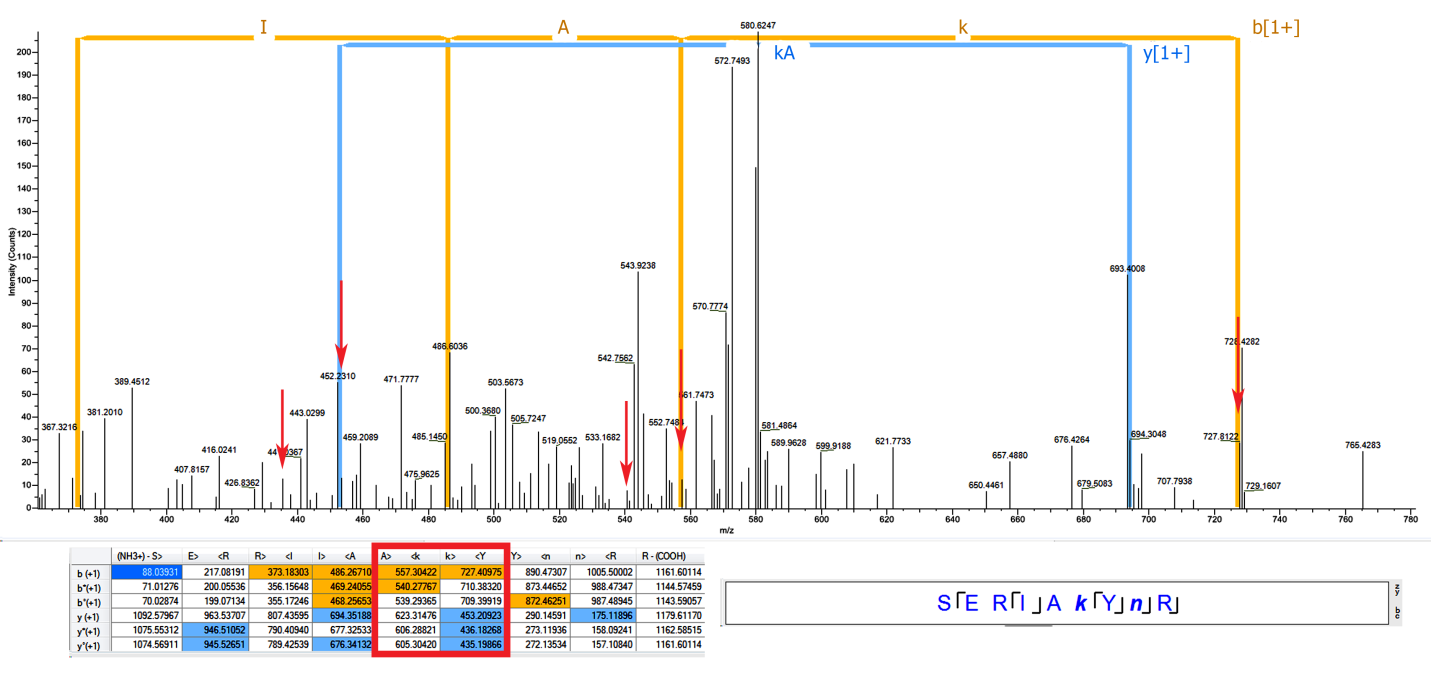


**Fig. E7.** MS/MS spectrum of Polymorphic membrane protein B (Q2TGM5) acetylated peptide ISMDLK*EPSK

[Figure is double column wide, dimensions 19 cm x 11 cm, 500ppi]


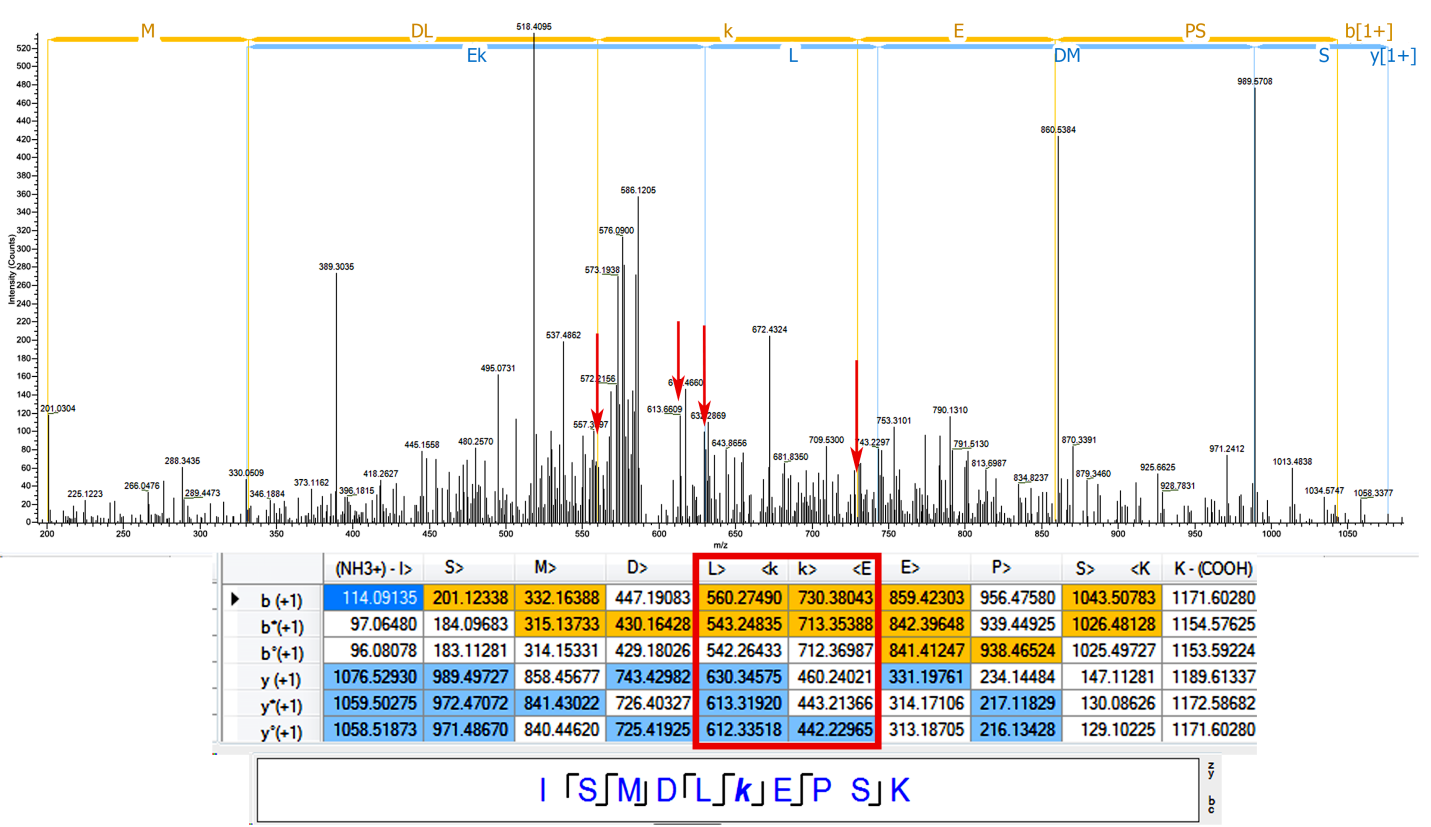


**Fig. E8.** MS/MS spectrum of Polymorphic membrane protein E (Q84FV6) acetylated peptide GGAIYIDGTSnSK*ISADR

[Figure is double column wide, dimensions 19 cm x 9.5 cm, 500ppi]


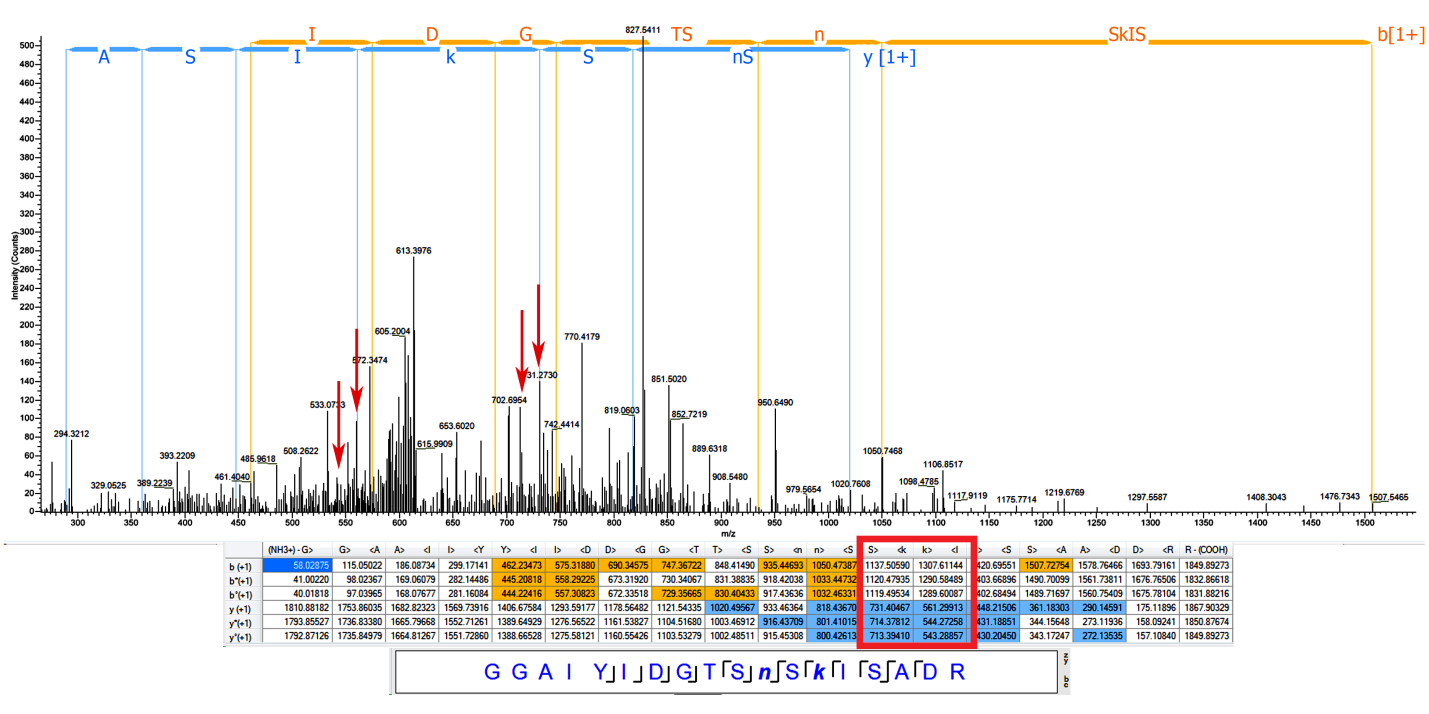


**Fig. E9.** MS/MS spectrum of Polymorphic membrane protein F (Q2TGH5) acetylated peptide GSHSLK*FSHLK

[Figure is double column wide, dimensions 19 cm x 10 cm, 500ppi]


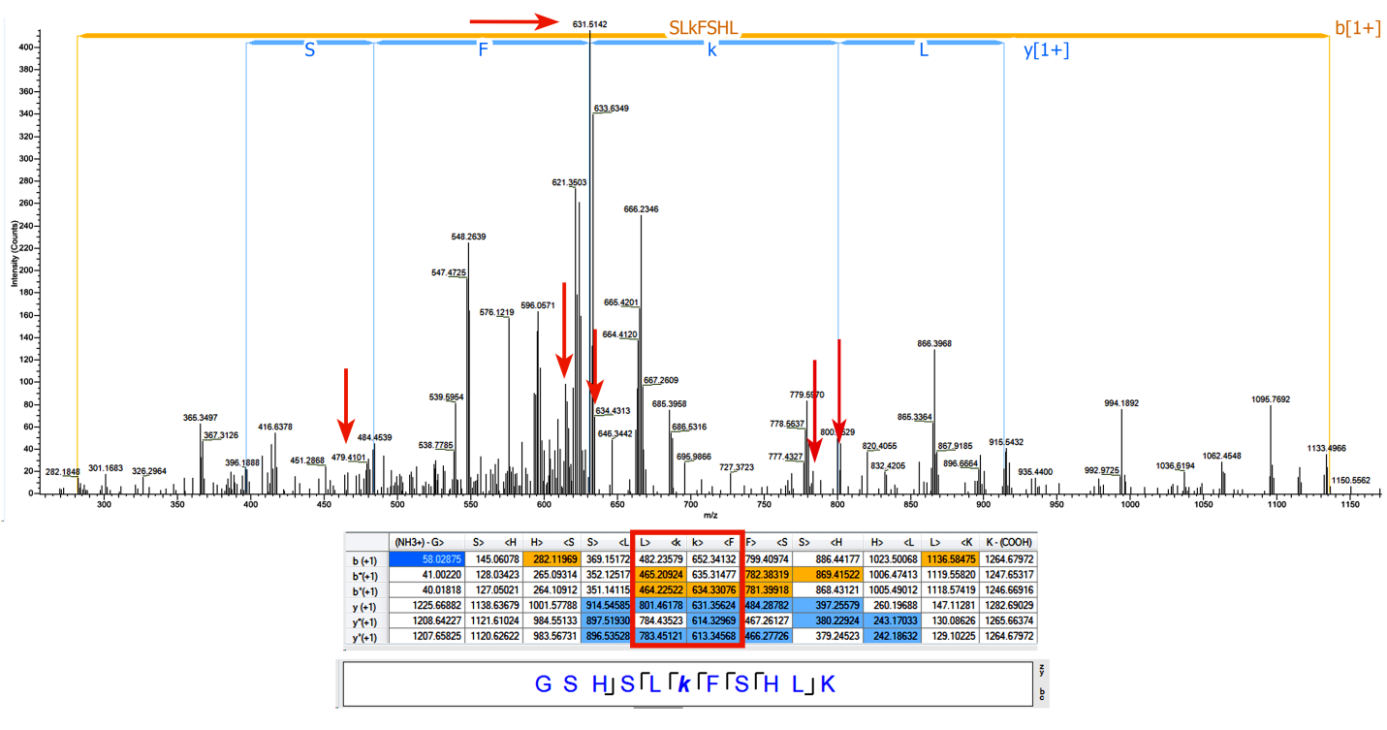


**Fig. E10**. Acetylated CtB band detection, estimation of band intensities and molecular weight. [Figure is single column wide, dimensions 9 cm x 17cm, 500ppi]


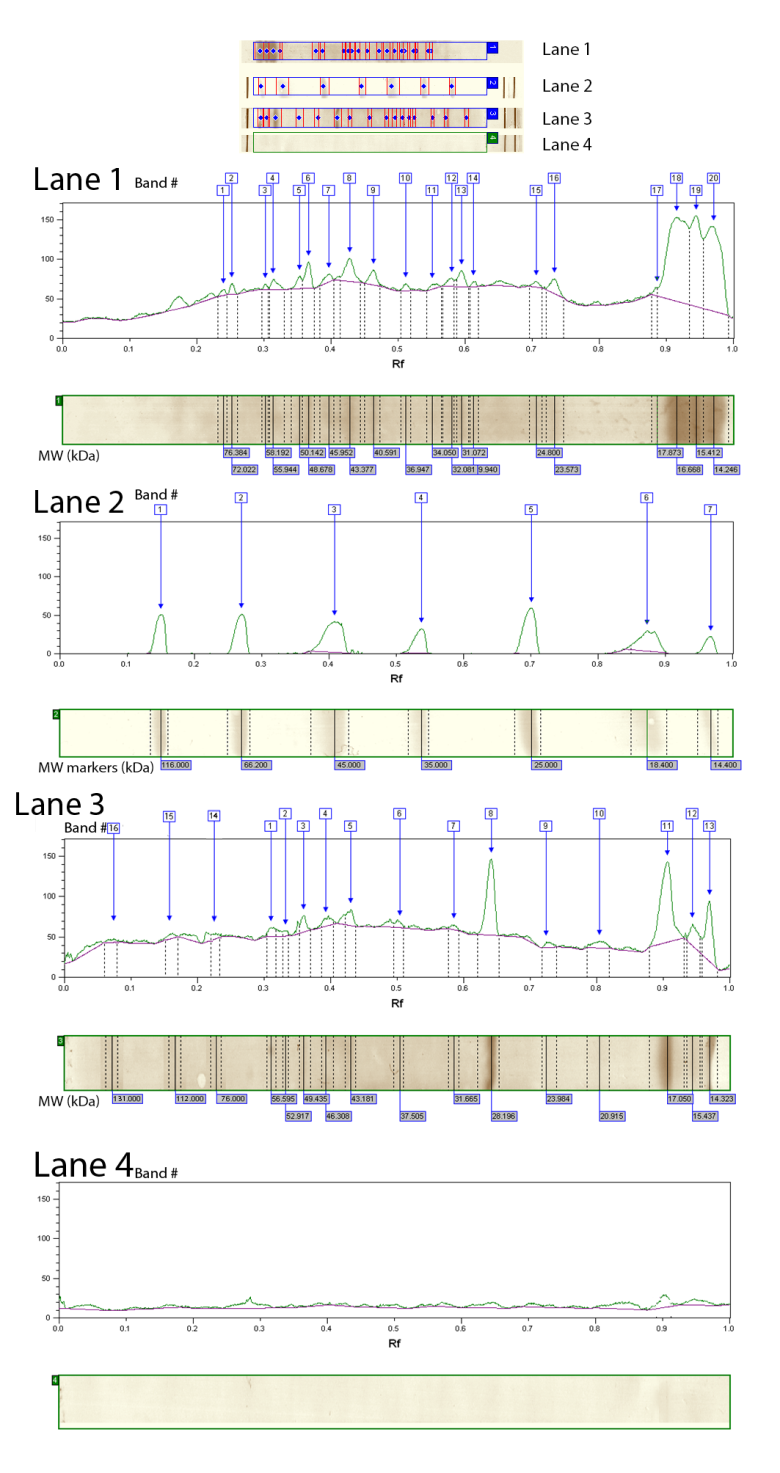


**Table E1**. SEQUEST identification of 263 CtB protein hits with peptide FDR of 5%. Abbreviations: Cov. = protein sequence coverage, # Uniq. Pep. = number of unique peptides, # PSMs = number of peptide-spectrum matches

| **Accession** | **Description** | **Score** | **Cov. [%]** | **# Uniq. Pep.** | **# PSMs** | **MW [kDa]** | **calc. pI** |
| --- | --- | --- | --- | --- | --- | --- | --- |
| P23421 | Major outer membrane porin, serovar B OS=Chlamydia trachomatis GN=ompA PE=2 SV=1 - [MOMPB_CHLTH] | 5588.16 | 77.92 | 8 | 3684 | 42.5 | 5.34 |
| Q3KMQ9 | 60 kDa chaperonin OS=Chlamydia trachomatis serovar A (strain ATCC VR-571B / DSM 19440 / HAR-13) GN=groL PE=2 SV=3 - [CH60_CHLTA] | 2448.06 | 86.21 | 30 | 2079 | 58.1 | 5.35 |
| A0A0E9B231 | Major outer membrane porin OS=Chlamydia trachomatis GN=ompA PE=3 SV=1 - [A0A0E9B231_CHLTH] | 2156.77 | 45.29 | 1 | 1684 | 42.5 | 5.25 |
| Q6U5G5 | Major outer membrane porin (Fragment) OS=Chlamydia trachomatis GN=omp1 PE=3 SV=1 - [Q6U5G5_CHLTH] | 2077.27 | 88.89 | 1 | 1706 | 31.2 | 5.10 |
| A0A0E9GFA8 | FKBP-type peptidyl-prolyl cis-trans isomerase (Trigger factor) OS=Chlamydia trachomatis GN=ERS133248_06388 PE=4 SV=1 - [A0A0E9GFA8_CHLTH] | 1147.64 | 88.00 | 1 | 5641 | 25.8 | 9.06 |
| Q6JBB5 | Major outer membrane porin (Fragment) OS=Chlamydia trachomatis GN=ompA PE=3 SV=1 - [Q6JBB5_CHLTH] | 882.47 | 46.69 | 1 | 847 | 27.5 | 5.00 |
| G4NMG9 | Elongation factor Tu OS=Chlamydia trachomatis serovar A (strain A2497) GN=tuf PE=3 SV=1 - [G4NMG9_CHLT4] | 838.85 | 100.00 | 20 | 813 | 43.9 | 5.54 |
| O84631 | 30S ribosomal protein S4 OS=Chlamydia trachomatis (strain D/UW-3/Cx) GN=rpsD PE=3 SV=1 - [RS4_CHLTR] | 717.65 | 79.90 | 1 | 1114 | 23.7 | 9.99 |
| A1X2C1 | Major outer membrane porin OS=Chlamydia trachomatis GN=ompA PE=3 SV=1 - [A1X2C1_CHLTH] | 564.44 | 22.28 | 1 | 668 | 42.5 | 5.43 |
| G4NNI8 | Outer membrane protein OS=Chlamydia trachomatis serovar A (strain A2497) GN=CTO_0483 PE=4 SV=1 - [G4NNI8_CHLT4] | 479.85 | 61.84 | 12 | 447 | 59.3 | 7.21 |
| G4NMY4 | Chaperone protein DnaK OS=Chlamydia trachomatis serovar A (strain A2497) GN=dnaK PE=2 SV=1 - [G4NMY4_CHLT4] | 472.07 | 86.12 | 14 | 781 | 71.2 | 5.14 |
| Q2TV29 | Polymorphic membrane protein D OS=Chlamydia trachomatis GN=pmpD PE=4 SV=1 - [Q2TV29_CHLTH] | 397.14 | 58.39 | 9 | 542 | 160.6 | 4.86 |
| A0A0E9FG31 | Uncharacterized membrane-bound protein conserved in bacteria OS=Chlamydia trachomatis GN=ERS095036_13607 PE=4 SV=1 - [A0A0E9FG31_CHLTH] | 393.45 | 65.33 | 1 | 278 | 25.3 | 9.45 |
| A0A0E9CLT4 | ATP synthase subunit beta OS=Chlamydia trachomatis GN=sctN_2 PE=3 SV=1 - [A0A0E9CLT4_CHLTH] | 359.81 | 67.36 | 3 | 311 | 53.6 | 6.49 |
| Q2TCJ4 | Polymorphic membrane protein G OS=Chlamydia trachomatis GN=pmpG PE=4 SV=1 - [Q2TCJ4_CHLTH] | 355.73 | 44.32 | 10 | 450 | 107.3 | 5.60 |
| A0A0E9AUH9 | Uncharacterised protein OS=Chlamydia trachomatis GN=ERS066953_01257 PE=4 SV=1 - [A0A0E9AUH9_CHLTH] | 335.20 | 77.64 | 1 | 350 | 18.6 | 5.25 |
| A0A0E9DIU3 | Gag gene protein p24 (Core nucleocapsid protein) OS=Chlamydia trachomatis GN=ERS082929_01031 PE=4 SV=1 - [A0A0E9DIU3_CHLTH] | 324.94 | 78.57 | 6 | 546 | 65.7 | 8.78 |
| G4NM38 | Manganese-binding protein OS=Chlamydia trachomatis serovar A (strain A2497) GN=CTO_0072 PE=3 SV=1 - [G4NM38_CHLT4] | 320.50 | 92.00 | 9 | 306 | 39.8 | 5.90 |
| G4NMM0 | Uncharacterized protein OS=Chlamydia trachomatis serovar A (strain A2497) GN=CTO_0629 PE=4 SV=1 - [G4NMM0_CHLT4] | 315.38 | 79.73 | 8 | 343 | 44.0 | 9.23 |
| A0A0E9CQD9 | CHLPN 76 kD protein-like OS=Chlamydia trachomatis GN=ERS075195_00406 PE=4 SV=1 - [A0A0E9CQD9_CHLTH] | 283.82 | 85.43 | 11 | 324 | 50.0 | 8.18 |
| A0A0E9DC36 | Gag gene protein p24 (Core nucleocapsid protein) OS=Chlamydia trachomatis GN=ERS082928_02139 PE=4 SV=1 - [A0A0E9DC36_CHLTH] | 282.46 | 82.87 | 1 | 262 | 31.6 | 8.91 |
| A0A0E9B124 | Transaldolase OS=Chlamydia trachomatis GN=talA PE=3 SV=1 - [A0A0E9B124_CHLTH] | 216.87 | 70.95 | 7 | 307 | 36.1 | 5.03 |
| A0A0E9GER6 | Sensor histidine kinase graS OS=Chlamydia trachomatis GN=graS_2 PE=4 SV=1 - [A0A0E9GER6_CHLTH] | 208.60 | 72.34 | 1 | 424 | 32.6 | 9.31 |
| O84681 | Probable Yop proteins translocation protein C/general secretion pathway protein OS=Chlamydia trachomatis (strain D/UW-3/Cx) GN=yscC PE=3 SV=1 - [O84681_CHLTR] | 185.56 | 52.12 | 8 | 696 | 100.5 | 5.77 |
| G4NP64 | Outer membrane protein OS=Chlamydia trachomatis serovar A (strain A2497) GN=CTO_0263 PE=4 SV=1 - [G4NP64_CHLT4] | 183.78 | 66.29 | 6 | 451 | 88.7 | 9.03 |
| A0A0E9AZX3 | Uncharacterised protein OS=Chlamydia trachomatis GN=ERS066953_00091 PE=4 SV=1 - [A0A0E9AZX3_CHLTH] | 181.93 | 62.99 | 2 | 571 | 41.8 | 9.79 |
| A0A0E9DHV0 | Transcription termination factor Rho OS=Chlamydia trachomatis GN=rho PE=3 SV=1 - [A0A0E9DHV0_CHLTH] | 180.72 | 76.08 | 7 | 336 | 51.7 | 7.36 |
| A0A0E9CRR3 | Putative nucleotide transport protein OS=Chlamydia trachomatis GN=tlcA_1 PE=4 SV=1 - [A0A0E9CRR3_CHLTH] | 178.42 | 22.83 | 1 | 211 | 24.4 | 9.95 |
| A0A0H2X3D0 | DO serine protease OS=Chlamydia trachomatis serovar A (strain ATCC VR-571B / DSM 19440 / HAR-13) GN=htrA PE=4 SV=1 - [A0A0H2X3D0_CHLTA] | 175.01 | 44.67 | 4 | 269 | 53.3 | 7.02 |
| G4NMP7 | Thioredoxin peroxidase OS=Chlamydia trachomatis serovar A (strain A2497) GN=CTO_0654 PE=4 SV=1 - [G4NMP7_CHLT4] | 173.43 | 90.15 | 7 | 162 | 22.7 | 5.01 |
| A0A0E9EQQ0 | Uncharacterized protein involved in outer membrane biogenesis OS=Chlamydia trachomatis GN=ERS095036_05921 PE=4 SV=1 - [A0A0E9EQQ0_CHLTH] | 172.61 | 69.47 | 1 | 1193 | 165.2 | 6.83 |
| O84588 | Protein CT_584 OS=Chlamydia trachomatis (strain D/UW-3/Cx) GN=CT_584 PE=3 SV=1 - [Y584_CHLTR] | 171.18 | 95.63 | 6 | 215 | 21.1 | 5.87 |
| G4NMJ8 | Type III secretion cytoplasmic membrane protein SctJ OS=Chlamydia trachomatis serovar A (strain A2497) GN=CTO_0609 PE=4 SV=1 - [G4NMJ8_CHLT4] | 170.62 | 66.47 | 5 | 402 | 37.0 | 6.24 |
| A0A0H2X1M5 | Phosphopeptide binding protein OS=Chlamydia trachomatis serovar A (strain ATCC VR-571B / DSM 19440 / HAR-13) GN=CTA_0721 PE=4 SV=1 - [A0A0H2X1M5_CHLTA] | 168.83 | 48.25 | 8 | 214 | 89.6 | 4.60 |
| B0B8W9 | Histone H1-like protein Hc1 OS=Chlamydia trachomatis serovar L2 (strain 434/Bu / ATCC VR-902B) GN=hctA PE=1 SV=1 - [HCT1_CHLT2] | 160.66 | 91.20 | 3 | 512 | 13.7 | 10.70 |
| A0A0E9CZH0 | Putative membrane transport protein OS=Chlamydia trachomatis GN=invA PE=4 SV=1 - [A0A0E9CZH0_CHLTH] | 159.20 | 62.01 | 3 | 381 | 77.9 | 8.07 |
| G4NNI1 | Elongation factor G OS=Chlamydia trachomatis serovar A (strain A2497) GN=fusA PE=3 SV=1 - [G4NNI1_CHLT4] | 156.39 | 79.77 | 6 | 367 | 77.4 | 5.34 |
| A0A0E9FQ09 | ATP synthase subunit alpha OS=Chlamydia trachomatis GN=sctN_2 PE=3 SV=1 - [A0A0E9FQ09_CHLTH] | 153.28 | 57.34 | 1 | 244 | 55.4 | 5.12 |
| Q3KMX6 | Probable cytosol aminopeptidase OS=Chlamydia trachomatis serovar A (strain ATCC VR-571B / DSM 19440 / HAR-13) GN=pepA PE=3 SV=1 - [AMPA_CHLTA] | 153.21 | 76.15 | 9 | 211 | 54.0 | 6.00 |
| A0A0E9EUP2 | ATP synthase subunit alpha OS=Chlamydia trachomatis GN=sctN_2 PE=3 SV=1 - [A0A0E9EUP2_CHLTH] | 148.70 | 35.91 | 1 | 186 | 56.1 | 5.05 |
| Q2TGM5 | Polymorphic membrane protein B OS=Chlamydia trachomatis GN=pmpB PE=4 SV=1 - [Q2TGM5_CHLTH] | 147.60 | 40.61 | 8 | 480 | 182.9 | 6.02 |
| G4NPG0 | Peptidyl-prolyl cis-trans isomerase OS=Chlamydia trachomatis serovar A (strain A2497) GN=CTO_0591 PE=4 SV=1 - [G4NPG0_CHLT4] | 146.09 | 98.00 | 6 | 193 | 27.6 | 5.55 |
| Q6JBC5 | Major outer membrane porin (Fragment) OS=Chlamydia trachomatis GN=ompA PE=3 SV=1 - [Q6JBC5_CHLTH] | 143.13 | 23.35 | 1 | 100 | 27.6 | 5.43 |
| A0A0E9CZX7 | Type III secretion system ATPase OS=Chlamydia trachomatis GN=sctN PE=4 SV=1 - [A0A0E9CZX7_CHLTH] | 142.61 | 76.24 | 8 | 223 | 48.2 | 5.81 |
| O84591 | Enolase OS=Chlamydia trachomatis (strain D/UW-3/Cx) GN=eno PE=3 SV=1 - [ENO_CHLTR] | 141.08 | 46.23 | 6 | 170 | 45.4 | 4.73 |
| A0A0E9D6X4 | Putative membrane associated protein OS=Chlamydia trachomatis GN=ERS075185_00609 PE=4 SV=1 - [A0A0E9D6X4_CHLTH] | 140.57 | 87.78 | 1 | 42 | 9.8 | 8.60 |
| Q3KMQ8 | 10 kDa chaperonin OS=Chlamydia trachomatis serovar A (strain ATCC VR-571B / DSM 19440 / HAR-13) GN=groS PE=3 SV=1 - [CH10_CHLTA] | 133.53 | 100.00 | 2 | 164 | 11.2 | 4.94 |
| P0CE08 | DNA-directed RNA polymerase subunit alpha OS=Chlamydia trachomatis (strain D/UW-3/Cx) GN=rpoA PE=3 SV=1 - [RPOA_CHLTR] | 133.17 | 75.33 | 5 | 876 | 41.8 | 5.47 |
| G4NM27 | Polymorphic outer membrane protein OS=Chlamydia trachomatis serovar A (strain A2497) GN=CTO_0952 PE=4 SV=1 - [G4NM27_CHLT4] | 132.64 | 49.66 | 7 | 219 | 108.7 | 6.77 |
| O84805 | 50S ribosomal protein L25 OS=Chlamydia trachomatis (strain D/UW-3/Cx) GN=rplY PE=3 SV=1 - [RL25_CHLTR] | 131.93 | 83.78 | 3 | 149 | 20.4 | 8.76 |
| P0CE13 | Glyceraldehyde-3-phosphate dehydrogenase OS=Chlamydia trachomatis (strain D/UW-3/Cx) GN=gap PE=3 SV=1 - [G3P_CHLTR] | 131.45 | 60.48 | 5 | 160 | 36.3 | 5.90 |
| A0A0E9D9R5 | Conjugal transfer ATP-binding protein TraC OS=Chlamydia trachomatis GN=ERS082928_01152 PE=4 SV=1 - [A0A0E9D9R5_CHLTH] | 130.40 | 100.00 | 1 | 851 | 18.6 | 9.07 |
| A0A0E9FFW1 | Sigma-54 dependent response regulator OS=Chlamydia trachomatis GN=arlR_5 PE=4 SV=1 - [A0A0E9FFW1_CHLTH] | 130.19 | 64.98 | 1 | 128 | 24.9 | 5.24 |
| Q2TGH5 | Polymorphic membrane protein F OS=Chlamydia trachomatis GN=pmpF PE=4 SV=1 - [Q2TGH5_CHLTH] | 125.11 | 51.55 | 7 | 342 | 112.7 | 8.43 |
| G4NMA4 | 30S ribosomal protein S9 OS=Chlamydia trachomatis serovar A (strain A2497) GN=rpsI PE=3 SV=1 - [G4NMA4_CHLT4] | 123.69 | 92.48 | 2 | 243 | 15.0 | 11.03 |
| B0B8Q5 | Elongation factor Ts OS=Chlamydia trachomatis serovar L2 (strain 434/Bu / ATCC VR-902B) GN=tsf PE=3 SV=1 - [EFTS_CHLT2] | 120.65 | 90.78 | 5 | 188 | 30.9 | 5.90 |
| A0A0E9GBH4 | Fimbrial subunit type 1 OS=Chlamydia trachomatis GN=ERS133248_04370 PE=4 SV=1 - [A0A0E9GBH4_CHLTH] | 119.62 | 85.60 | 1 | 1099 | 53.7 | 7.68 |
| A0A0E9FTZ2 | Plasmid recombination enzyme OS=Chlamydia trachomatis GN=ERS133249_01967 PE=4 SV=1 - [A0A0E9FTZ2_CHLTH] | 118.23 | 85.42 | 1 | 668 | 56.4 | 8.63 |
| G4NN42 | UPF0109 protein CTO_0716 OS=Chlamydia trachomatis serovar A (strain A2497) GN=CTO_0716 PE=3 SV=1 - [G4NN42_CHLT4] | 113.93 | 100.00 | 6 | 114 | 9.2 | 9.04 |
| Q83U05 | Polymorphic membrane protein E (Fragment) OS=Chlamydia trachomatis GN=pmpE PE=4 SV=1 - [Q83U05_CHLTH] | 113.56 | 44.92 | 5 | 236 | 101.6 | 7.21 |
| A1X5G7 | 30S ribosomal protein S2 OS=Chlamydia trachomatis GN=rs2 PE=3 SV=1 - [A1X5G7_CHLTH] | 113.18 | 79.79 | 7 | 209 | 31.1 | 6.70 |
| A0A0E9DGI7 | 30S ribosomal protein S3 OS=Chlamydia trachomatis GN=rpsC PE=3 SV=1 - [A0A0E9DGI7_CHLTH] | 108.61 | 95.54 | 2 | 301 | 24.3 | 10.02 |
| O84849 | Polyribonucleotide nucleotidyltransferase OS=Chlamydia trachomatis (strain D/UW-3/Cx) GN=pnp PE=3 SV=1 - [PNP_CHLTR] | 104.78 | 57.84 | 6 | 600 | 75.5 | 5.78 |
| Q58HD1 | Major outer membrane porin (Fragment) OS=Chlamydia trachomatis GN=omp1 PE=3 SV=1 - [Q58HD1_CHLTH] | 103.49 | 24.60 | 1 | 65 | 33.9 | 4.88 |
| A0A0E9FSE8 | 60 kDa chaperonin OS=Chlamydia trachomatis GN=groEL_2 PE=3 SV=1 - [A0A0E9FSE8_CHLTH] | 100.29 | 81.66 | 1 | 164 | 24.6 | 5.54 |
| A0A0E9AUP2 | ATPase associated with various cellular activities family protein OS=Chlamydia trachomatis GN=clpB_3 PE=4 SV=1 - [A0A0E9AUP2_CHLTH] | 99.90 | 78.72 | 1 | 358 | 21.4 | 7.47 |
| B0B882 | 30S ribosomal protein S11 OS=Chlamydia trachomatis serovar L2 (strain 434/Bu / ATCC VR-902B) GN=rpsK PE=3 SV=1 - [RS11_CHLT2] | 99.66 | 81.82 | 4 | 152 | 13.8 | 11.25 |
| G4NNU8 | Uncharacterized protein OS=Chlamydia trachomatis serovar A (strain A2497) GN=CTO_0001 PE=4 SV=1 - [G4NNU8_CHLT4] | 99.24 | 68.08 | 3 | 655 | 66.0 | 7.34 |
| A0A0E9G5H8 | Maltodextrin-binding protein mdxE OS=Chlamydia trachomatis GN=mdxE_1 PE=4 SV=1 - [A0A0E9G5H8_CHLTH] | 94.52 | 49.89 | 1 | 179 | 45.7 | 4.96 |
| A0A0E9B081 | ATP-dependent Clp protease%2C subunit B OS=Chlamydia trachomatis GN=clpB PE=3 SV=1 - [A0A0E9B081_CHLTH] | 89.17 | 78.20 | 6 | 476 | 96.6 | 5.47 |
| B0B9D5 | 50S ribosomal protein L19 OS=Chlamydia trachomatis serovar L2 (strain 434/Bu / ATCC VR-902B) GN=rplS PE=3 SV=1 - [RL19_CHLT2] | 87.62 | 88.43 | 1 | 188 | 13.1 | 9.94 |
| B2XRL5 | TarP OS=Chlamydia trachomatis GN=tarP PE=4 SV=1 - [B2XRL5_CHLTH] | 87.09 | 39.96 | 5 | 133 | 111.7 | 4.44 |
| P38002 | ATP-dependent Clp protease proteolytic subunit 1 OS=Chlamydia trachomatis (strain D/UW-3/Cx) GN=clpP1 PE=3 SV=3 - [CLPP1_CHLTR] | 86.87 | 78.65 | 2 | 102 | 21.1 | 5.59 |
| G4NM69 | Transcription termination/antitermination protein NusA OS=Chlamydia trachomatis serovar A (strain A2497) GN=nusA PE=3 SV=1 - [G4NM69_CHLT4] | 84.11 | 79.95 | 4 | 170 | 48.9 | 5.14 |
| B0BCD9 | Nucleoside diphosphate kinase OS=Chlamydia trachomatis serovar L2b (strain UCH-1/proctitis) GN=ndk PE=3 SV=1 - [NDK_CHLTB] | 83.56 | 51.06 | 2 | 118 | 15.3 | 5.49 |
| A0A0E9DPP4 | Putative protease OS=Chlamydia trachomatis GN=ERS095037_01179 PE=4 SV=1 - [A0A0E9DPP4_CHLTH] | 82.35 | 63.88 | 5 | 255 | 65.9 | 6.58 |
| A0A0E9DN19 | Putative type III secretion system membrane protein OS=Chlamydia trachomatis GN=copB PE=4 SV=1 - [A0A0E9DN19_CHLTH] | 77.73 | 56.26 | 1 | 999 | 50.3 | 9.13 |
| K0GGT0 | Ribosome-recycling factor OS=Chlamydia trachomatis GN=frr PE=3 SV=1 - [K0GGT0_CHLTH] | 74.70 | 90.50 | 3 | 186 | 20.0 | 8.56 |
| O84217 | Probable fructose-bisphosphate aldolase class 1 OS=Chlamydia trachomatis (strain D/UW-3/Cx) GN=fbaB PE=3 SV=1 - [ALF1_CHLTR] | 73.61 | 63.51 | 5 | 166 | 38.0 | 6.79 |
| A0A0E9D869 | Protein disulfide isomerase OS=Chlamydia trachomatis GN=dsbH_2 PE=4 SV=1 - [A0A0E9D869_CHLTH] | 73.37 | 84.76 | 3 | 83 | 18.5 | 8.32 |
| Q3KM43 | 50S ribosomal protein L11 OS=Chlamydia trachomatis serovar A (strain ATCC VR-571B / DSM 19440 / HAR-13) GN=rplK PE=3 SV=1 - [RL11_CHLTA] | 72.34 | 100.00 | 3 | 169 | 15.1 | 9.67 |
| Q3KLI8 | 30S ribosomal protein S13 OS=Chlamydia trachomatis serovar A (strain ATCC VR-571B / DSM 19440 / HAR-13) GN=rpsM PE=3 SV=1 - [RS13_CHLTA] | 69.99 | 94.26 | 3 | 202 | 13.9 | 11.02 |
| O84100 | 30S ribosomal protein S1 OS=Chlamydia trachomatis (strain D/UW-3/Cx) GN=rpsA PE=3 SV=1 - [RS1_CHLTR] | 69.90 | 62.74 | 5 | 189 | 63.5 | 5.27 |
| A0A0E9G077 | Putative UDP-N-acetylglucosamine--N-acetylmuramyl-(Pentapeptide) pyrophosphoryl-undecaprenol N-acetylglucosamine transferase OS=Chlamydia trachomatis GN=murG_2 PE=4 SV=1 - [A0A0E9G077_CHLTH] | 69.88 | 54.44 | 1 | 217 | 37.5 | 9.54 |
| B0B895 | 50S ribosomal protein L16 OS=Chlamydia trachomatis serovar L2 (strain 434/Bu / ATCC VR-902B) GN=rplP PE=3 SV=1 - [RL16_CHLT2] | 69.18 | 88.41 | 2 | 185 | 15.8 | 11.30 |
| H6V6T8 | DNA-directed RNA polymerase subunit beta OS=Chlamydia trachomatis (strain L2c) GN=rpoB PE=3 SV=1 - [H6V6T8_CHLTC] | 68.86 | 69.73 | 2 | 403 | 140.0 | 5.91 |
| Q6HA48 | PmpC OS=Chlamydia trachomatis GN=pmpC PE=4 SV=1 - [Q6HA48_CHLTH] | 68.61 | 38.85 | 4 | 531 | 187.9 | 4.61 |
| Q83TT3 | Major outer membrane protein OS=Chlamydia trachomatis GN=porB PE=4 SV=1 - [Q83TT3_CHLTH] | 68.26 | 63.53 | 2 | 126 | 37.3 | 5.39 |
| B0B930 | 50S ribosomal protein L9 OS=Chlamydia trachomatis serovar L2 (strain 434/Bu / ATCC VR-902B) GN=rplI PE=3 SV=1 - [RL9_CHLT2] | 67.72 | 92.81 | 1 | 153 | 18.4 | 6.55 |
| A0A0E9AR22 | Uncharacterized protein OS=Chlamydia trachomatis GN=greA_1 PE=3 SV=1 - [A0A0E9AR22_CHLTH] | 67.31 | 66.71 | 7 | 496 | 80.9 | 5.38 |
| P0A4C8 | 30S ribosomal protein S5 OS=Chlamydia trachomatis (strain D/UW-3/Cx) GN=rpsE PE=3 SV=1 - [RS5_CHLTR] | 65.70 | 66.06 | 3 | 244 | 17.8 | 9.85 |
| Q3KM48 | DNA-directed RNA polymerase subunit beta' OS=Chlamydia trachomatis serovar A (strain ATCC VR-571B / DSM 19440 / HAR-13) GN=rpoC PE=3 SV=1 - [RPOC_CHLTA] | 65.61 | 63.97 | 5 | 794 | 154.8 | 7.44 |
| Q84FU2 | Polymorphic membrane protein I (Fragment) OS=Chlamydia trachomatis GN=pmpI PE=4 SV=1 - [Q84FU2_CHLTH] | 65.56 | 45.63 | 2 | 184 | 92.0 | 6.60 |
| A4IET9 | Cation efflux system protein OS=Chlamydia trachomatis GN=ERS066953_00279 PE=4 SV=1 - [A4IET9_CHLTH] | 63.89 | 80.15 | 4 | 201 | 60.9 | 6.21 |
| A0A0E9CC48 | Enoyl-ACP reductase OS=Chlamydia trachomatis GN=fabI PE=4 SV=1 - [A0A0E9CC48_CHLTH] | 63.04 | 42.28 | 3 | 92 | 31.9 | 5.66 |
| B0B8T1 | ATP-dependent Clp protease ATP-binding subunit ClpX OS=Chlamydia trachomatis serovar L2 (strain 434/Bu / ATCC VR-902B) GN=clpX PE=3 SV=1 - [CLPX_CHLT2] | 62.51 | 89.50 | 5 | 193 | 46.2 | 5.72 |
| B0B899 | 50S ribosomal protein L2 OS=Chlamydia trachomatis serovar L2 (strain 434/Bu / ATCC VR-902B) GN=rplB PE=3 SV=1 - [RL2_CHLT2] | 62.02 | 91.20 | 5 | 156 | 31.5 | 10.55 |
| A0A0E9CU90 | ATP-dependent zinc metalloprotease FtsH OS=Chlamydia trachomatis GN=ftsH PE=3 SV=1 - [A0A0E9CU90_CHLTH] | 62.01 | 68.89 | 3 | 441 | 101.6 | 6.19 |
| G4NMV6 | Uncharacterized protein OS=Chlamydia trachomatis serovar A (strain A2497) GN=CTO_0404 PE=4 SV=1 - [G4NMV6_CHLT4] | 59.78 | 57.94 | 3 | 103 | 52.0 | 8.95 |
| O84066 | 6-phosphogluconate dehydrogenase, decarboxylating OS=Chlamydia trachomatis (strain D/UW-3/Cx) GN=gnd PE=3 SV=1 - [6PGD_CHLTR] | 59.62 | 58.54 | 5 | 97 | 52.6 | 5.44 |
| B0B7Z0 | 50S ribosomal protein L21 OS=Chlamydia trachomatis serovar L2 (strain 434/Bu / ATCC VR-902B) GN=rplU PE=3 SV=1 - [RL21_CHLT2] | 59.11 | 96.26 | 2 | 89 | 12.2 | 9.28 |
| A0A0H2X0S9 | 60 kDa inner membrane protein OS=Chlamydia trachomatis serovar A (strain ATCC VR-571B / DSM 19440 / HAR-13) GN=CTA_0273 PE=4 SV=1 - [A0A0H2X0S9_CHLTA] | 58.71 | 46.89 | 1 | 347 | 88.0 | 8.81 |
| B0B928 | 30S ribosomal protein S6 OS=Chlamydia trachomatis serovar L2 (strain 434/Bu / ATCC VR-902B) GN=rpsF PE=3 SV=1 - [RS6_CHLT2] | 58.62 | 57.14 | 2 | 68 | 12.9 | 8.81 |
| A0A0E9CT27 | Integration host factor beta-subunit OS=Chlamydia trachomatis GN=hupA PE=3 SV=1 - [A0A0E9CT27_CHLTH] | 58.48 | 93.24 | 1 | 478 | 17.0 | 10.61 |
| A0A0E9G085 | Leucine dehydrogenase OS=Chlamydia trachomatis GN=ldh PE=3 SV=1 - [A0A0E9G085_CHLTH] | 58.37 | 61.04 | 5 | 86 | 33.0 | 5.29 |
| A0A0E9EH39 | Uncharacterised protein OS=Chlamydia trachomatis GN=ERS095036_03703 PE=4 SV=1 - [A0A0E9EH39_CHLTH] | 56.73 | 64.52 | 1 | 189 | 24.3 | 6.38 |
| Q3KLI3 | 50S ribosomal protein L6 OS=Chlamydia trachomatis serovar A (strain ATCC VR-571B / DSM 19440 / HAR-13) GN=rplF PE=3 SV=1 - [RL6_CHLTA] | 56.69 | 96.17 | 4 | 125 | 19.9 | 9.96 |
| A0A0E9CKW5 | Putative type III secretion system chaperone OS=Chlamydia trachomatis GN=ERS075193_00737 PE=4 SV=1 - [A0A0E9CKW5_CHLTH] | 56.54 | 80.84 | 1 | 414 | 18.4 | 5.16 |
| A0A0E9EVW0 | Septal ring factor OS=Chlamydia trachomatis GN=envC_1 PE=4 SV=1 - [A0A0E9EVW0_CHLTH] | 55.76 | 63.82 | 1 | 387 | 45.1 | 8.84 |
| A0A0E9DFJ6 | Protein of uncharacterised function%2C DUF582 OS=Chlamydia trachomatis GN=ERS082930_00129 PE=4 SV=1 - [A0A0E9DFJ6_CHLTH] | 55.22 | 47.84 | 1 | 283 | 92.6 | 5.01 |
| A0A0E9AS56 | Uncharacterised protein OS=Chlamydia trachomatis GN=ERS066953_00847 PE=4 SV=1 - [A0A0E9AS56_CHLTH] | 54.05 | 95.00 | 1 | 260 | 9.2 | 8.35 |
| G4NMD9 | Superoxide dismutase OS=Chlamydia trachomatis serovar A (strain A2497) GN=CTO_0316 PE=3 SV=1 - [G4NMD9_CHLT4] | 53.49 | 74.31 | 1 | 101 | 24.9 | 6.73 |
| A0A0E9GCU2 | Dihydroxyacetone kinase OS=Chlamydia trachomatis GN=ERS133248_04181 PE=4 SV=1 - [A0A0E9GCU2_CHLTH] | 53.06 | 52.44 | 1 | 339 | 59.8 | 4.64 |
| A0A0E9CYI8 | 50S ribosomal protein L10 OS=Chlamydia trachomatis GN=rplJ PE=3 SV=1 - [A0A0E9CYI8_CHLTH] | 52.54 | 75.00 | 3 | 120 | 18.8 | 6.34 |
| A0A0E9FV45 | Amino acid ABC transporter substrate-binding protein OS=Chlamydia trachomatis GN=fliY PE=4 SV=1 - [A0A0E9FV45_CHLTH] | 51.14 | 53.26 | 3 | 74 | 29.0 | 7.37 |
| A0A0E9E9K2 | Protein translocase subunit OS=Chlamydia trachomatis GN=ERS066953_01398 PE=4 SV=1 - [A0A0E9E9K2_CHLTH] | 50.57 | 51.75 | 1 | 140 | 12.8 | 9.20 |
| A0A0E9DQU8 | Chaperone protein DnaK OS=Chlamydia trachomatis GN=dnaK_1 PE=2 SV=1 - [A0A0E9DQU8_CHLTH] | 50.42 | 69.86 | 1 | 358 | 68.3 | 4.92 |
| A0A0E9FZX9 | DNA strand transferase OS=Chlamydia trachomatis GN=mobA PE=4 SV=1 - [A0A0E9FZX9_CHLTH] | 49.48 | 83.57 | 1 | 473 | 66.4 | 7.39 |
| A0A0E9DJ74 | 30S ribosomal protein S16 OS=Chlamydia trachomatis GN=rpsP PE=3 SV=1 - [A0A0E9DJ74_CHLTH] | 49.14 | 100.00 | 2 | 97 | 13.4 | 10.37 |
| A0A0E9CB95 | Alpha-1,4 glucan phosphorylase OS=Chlamydia trachomatis GN=glgP PE=3 SV=1 - [A0A0E9CB95_CHLTH] | 47.59 | 71.38 | 2 | 254 | 92.8 | 5.96 |
| G4NNS7 | 6,7-dimethyl-8-ribityllumazine synthase OS=Chlamydia trachomatis serovar A (strain A2497) GN=ribH PE=3 SV=1 - [G4NNS7_CHLT4] | 47.57 | 53.37 | 3 | 53 | 17.2 | 7.75 |
| A0A0E9FM37 | Uncharacterised protein OS=Chlamydia trachomatis GN=ERS095036_16868 PE=4 SV=1 - [A0A0E9FM37_CHLTH] | 47.18 | 86.36 | 1 | 77 | 7.5 | 8.34 |
| A0A0E9DXC5 | Chaperone protein HtpG OS=Chlamydia trachomatis GN=htpG PE=3 SV=1 - [A0A0E9DXC5_CHLTH] | 47.02 | 61.24 | 1 | 153 | 76.4 | 5.07 |
| Q3KLX6 | Glucose-6-phosphate isomerase OS=Chlamydia trachomatis serovar A (strain ATCC VR-571B / DSM 19440 / HAR-13) GN=pgi PE=3 SV=2 - [G6PI_CHLTA] | 45.54 | 53.63 | 8 | 53 | 57.6 | 6.06 |
| A0A0E9EQM9 | ECM-binding protein homolog OS=Chlamydia trachomatis GN=ebh PE=4 SV=1 - [A0A0E9EQM9_CHLTH] | 45.52 | 83.71 | 1 | 915 | 111.8 | 5.39 |
| A0A0E9CCK9 | Transcription termination/antitermination protein NusG OS=Chlamydia trachomatis GN=nusG PE=3 SV=1 - [A0A0E9CCK9_CHLTH] | 43.92 | 100.00 | 6 | 130 | 20.7 | 5.31 |
| A0A0E9G4T4 | Uncharacterised protein OS=Chlamydia trachomatis GN=ERS133250_01363 PE=4 SV=1 - [A0A0E9G4T4_CHLTH] | 43.86 | 93.62 | 1 | 109 | 5.3 | 9.63 |
| A0A0E9FU79 | Type III secretion chaperone OS=Chlamydia trachomatis GN=scc1 PE=4 SV=1 - [A0A0E9FU79_CHLTH] | 42.74 | 100.00 | 1 | 62 | 10.4 | 9.11 |
| A0A0E9EQ15 | Internalin-A OS=Chlamydia trachomatis GN=inlA_3 PE=4 SV=1 - [A0A0E9EQ15_CHLTH] | 42.72 | 74.45 | 1 | 314 | 50.6 | 5.16 |
| B0B891 | 50S ribosomal protein L24 OS=Chlamydia trachomatis serovar L2 (strain 434/Bu / ATCC VR-902B) GN=rplX PE=3 SV=1 - [RL24_CHLT2] | 42.14 | 90.09 | 1 | 308 | 12.6 | 10.43 |
| O84357 | Peptide deformylase OS=Chlamydia trachomatis (strain D/UW-3/Cx) GN=def PE=3 SV=1 - [DEF_CHLTR] | 41.90 | 88.95 | 4 | 64 | 20.5 | 5.96 |
| G4NNH5 | Serine hydroxymethyltransferase OS=Chlamydia trachomatis serovar A (strain A2497) GN=glyA PE=3 SV=1 - [G4NNH5_CHLT4] | 40.83 | 68.05 | 4 | 174 | 55.2 | 6.71 |
| B0B9B0 | Glutamyl-tRNA(Gln) amidotransferase subunit A OS=Chlamydia trachomatis serovar L2 (strain 434/Bu / ATCC VR-902B) GN=gatA PE=3 SV=1 - [GATA_CHLT2] | 40.32 | 61.30 | 4 | 246 | 53.6 | 6.23 |
| O84712 | ATP-dependent Clp protease proteolytic subunit 2 OS=Chlamydia trachomatis (strain D/UW-3/Cx) GN=clpP2 PE=3 SV=1 - [CLPP2_CHLTR] | 40.21 | 56.65 | 1 | 80 | 22.0 | 5.25 |
| A0A0E9D2R6 | MotA/TolQ/ExbB proton channel family protein OS=Chlamydia trachomatis GN=exbB PE=3 SV=1 - [A0A0E9D2R6_CHLTH] | 40.19 | 28.88 | 2 | 54 | 25.9 | 9.17 |
| G4NMW2 | Malate dehydrogenase OS=Chlamydia trachomatis serovar A (strain A2497) GN=mdh PE=3 SV=1 - [G4NMW2_CHLT4] | 39.56 | 61.13 | 1 | 153 | 37.1 | 7.87 |
| G4NND8 | DNA gyrase subunit A OS=Chlamydia trachomatis serovar A (strain A2497) GN=gyrA PE=3 SV=1 - [G4NND8_CHLT4] | 39.08 | 74.64 | 1 | 373 | 94.1 | 6.68 |
| A0A0E9GG53 | Galactoside transport system permease protein mglC OS=Chlamydia trachomatis GN=mglC PE=4 SV=1 - [A0A0E9GG53_CHLTH] | 38.80 | 17.54 | 1 | 60 | 30.1 | 8.85 |
| G4NPN0 | Ribonuclease E OS=Chlamydia trachomatis serovar A (strain A2497) GN=CTO_0880 PE=4 SV=1 - [G4NPN0_CHLT4] | 37.37 | 63.62 | 2 | 342 | 60.9 | 7.55 |
| A0A0E9DYC9 | Uncharacterized vancomycin resistance protein OS=Chlamydia trachomatis GN=ERS133246_02510 PE=4 SV=1 - [A0A0E9DYC9_CHLTH] | 37.10 | 60.87 | 1 | 463 | 64.8 | 8.31 |
| K0GB74 | CHLPN 76kDa-like protein OS=Chlamydia trachomatis GN=CT622 PE=4 SV=1 - [K0GB74_CHLTH] | 36.94 | 78.98 | 1 | 317 | 68.5 | 4.92 |
| A0A0E9C7V3 | 50S ribosomal protein L17 OS=Chlamydia trachomatis GN=rplQ PE=3 SV=1 - [A0A0E9C7V3_CHLTH] | 36.14 | 86.52 | 2 | 224 | 16.2 | 11.37 |
| B0B8V5 | Serine--tRNA ligase OS=Chlamydia trachomatis serovar L2 (strain 434/Bu / ATCC VR-902B) GN=serS PE=3 SV=1 - [SYS_CHLT2] | 35.77 | 49.30 | 1 | 228 | 48.4 | 6.09 |
| B0BAX0 | 4-hydroxy-3-methylbut-2-enyl diphosphate reductase OS=Chlamydia trachomatis serovar L2b (strain UCH-1/proctitis) GN=ispH PE=3 SV=1 - [ISPH_CHLTB] | 34.62 | 52.44 | 2 | 106 | 34.2 | 6.37 |
| A0A0E9AR09 | Thioredoxin reductase OS=Chlamydia trachomatis GN=trxB PE=3 SV=1 - [A0A0E9AR09_CHLTH] | 34.38 | 72.36 | 4 | 72 | 37.8 | 7.61 |
| A0A0E9CZR0 | Type III secretion system protein OS=Chlamydia trachomatis GN=sctQ PE=4 SV=1 - [A0A0E9CZR0_CHLTH] | 34.20 | 54.69 | 2 | 48 | 41.1 | 4.64 |
| B0B9B1 | Aspartyl/glutamyl-tRNA(Asn/Gln) amidotransferase subunit B OS=Chlamydia trachomatis serovar L2 (strain 434/Bu / ATCC VR-902B) GN=gatB PE=3 SV=1 - [GATB_CHLT2] | 34.14 | 53.28 | 3 | 127 | 54.9 | 6.32 |
| O84713 | Trigger factor OS=Chlamydia trachomatis (strain D/UW-3/Cx) GN=tig PE=3 SV=1 - [TIG_CHLTR] | 34.13 | 74.66 | 1 | 220 | 50.1 | 5.08 |
| A0A0E9DIS5 | 3-oxoacyl-[acyl-carrier-protein] synthase 2 OS=Chlamydia trachomatis GN=fabF PE=3 SV=1 - [A0A0E9DIS5_CHLTH] | 33.74 | 42.34 | 2 | 135 | 44.8 | 5.59 |
| A0A0E9C8B1 | 50S ribosomal protein L13 OS=Chlamydia trachomatis GN=rplM PE=3 SV=1 - [A0A0E9C8B1_CHLTH] | 33.62 | 100.00 | 1 | 129 | 16.8 | 10.13 |
| G4NMZ7 | DnaK suppressor protein OS=Chlamydia trachomatis serovar A (strain A2497) GN=CTO_0442 PE=4 SV=1 - [G4NMZ7_CHLT4] | 33.53 | 95.56 | 3 | 79 | 15.3 | 5.60 |
| G4NM99 | Elongation factor P OS=Chlamydia trachomatis serovar A (strain A2497) GN=efp PE=3 SV=1 - [G4NM99_CHLT4] | 30.91 | 87.57 | 1 | 115 | 20.5 | 5.25 |
| A0A0E9E330 | Anti-sigma factor antagonist OS=Chlamydia trachomatis GN=btrV PE=3 SV=1 - [A0A0E9E330_CHLTH] | 30.67 | 97.41 | 3 | 57 | 12.5 | 5.39 |
| K0G9B2 | Uridylate kinase OS=Chlamydia trachomatis GN=pyrH PE=3 SV=1 - [K0G9B2_CHLTH] | 30.39 | 68.16 | 2 | 56 | 26.1 | 5.48 |
| A0A0E9ERX5 | Uncharacterised protein OS=Chlamydia trachomatis GN=ERS095036_06689 PE=4 SV=1 - [A0A0E9ERX5_CHLTH] | 30.39 | 84.00 | 1 | 608 | 43.5 | 9.09 |
| A0A0E9EYP0 | Mevalonate kinase OS=Chlamydia trachomatis GN=ERS095036_08052 PE=4 SV=1 - [A0A0E9EYP0_CHLTH] | 30.30 | 36.76 | 1 | 44 | 40.5 | 6.35 |
| W8VNJ2 | Major outer membrane porin (Fragment) OS=Chlamydia trachomatis GN=ompA PE=3 SV=1 - [W8VNJ2_CHLTH] | 28.81 | 44.44 | 1 | 77 | 36.8 | 4.98 |
| B0B7Q0 | Pyruvate kinase OS=Chlamydia trachomatis serovar L2 (strain 434/Bu / ATCC VR-902B) GN=pyk PE=3 SV=1 - [KPYK_CHLT2] | 28.27 | 78.97 | 2 | 135 | 53.6 | 6.40 |
| G4NM39 | Manganese transport system ATP-binding protein OS=Chlamydia trachomatis serovar A (strain A2497) GN=CTO_0073 PE=3 SV=1 - [G4NM39_CHLT4] | 27.99 | 59.15 | 1 | 88 | 31.7 | 7.15 |
| K0GBL9 | Uncharacterized protein OS=Chlamydia trachomatis GN=CT694 PE=4 SV=1 - [K0GBL9_CHLTH] | 27.77 | 60.99 | 1 | 113 | 34.6 | 5.17 |
| B0B810 | 30S ribosomal protein S7 OS=Chlamydia trachomatis serovar L2 (strain 434/Bu / ATCC VR-902B) GN=rpsG PE=3 SV=1 - [RS7_CHLT2] | 27.67 | 98.09 | 1 | 183 | 17.8 | 9.83 |
| G4NN30 | Protein RecA OS=Chlamydia trachomatis serovar A (strain A2497) GN=recA PE=3 SV=1 - [G4NN30_CHLT4] | 27.40 | 63.47 | 3 | 130 | 41.9 | 9.04 |
| B0B889 | 30S ribosomal protein S8 OS=Chlamydia trachomatis serovar L2 (strain 434/Bu / ATCC VR-902B) GN=rpsH PE=3 SV=1 - [RS8_CHLT2] | 27.23 | 100.00 | 1 | 88 | 15.1 | 10.27 |
| O84125 | Biotin carboxyl carrier protein of acetyl-CoA carboxylase OS=Chlamydia trachomatis (strain D/UW-3/Cx) GN=accB PE=3 SV=1 - [BCCP_CHLTR] | 26.54 | 68.90 | 3 | 81 | 18.2 | 5.12 |
| A0A0E9ASB8 | 2-oxoglutarate dehydrogenase E1 component OS=Chlamydia trachomatis GN=sucA PE=4 SV=1 - [A0A0E9ASB8_CHLTH] | 26.22 | 49.39 | 1 | 243 | 102.5 | 5.48 |
| A0A0E9FMW1 | Putative phosphoenolpyruvate-protein phosphotransferase OS=Chlamydia trachomatis GN=ppdK PE=4 SV=1 - [A0A0E9FMW1_CHLTH] | 26.18 | 51.37 | 1 | 233 | 97.4 | 5.11 |
| O84757 | Elongation factor P 2 OS=Chlamydia trachomatis (strain D/UW-3/Cx) GN=efp2 PE=3 SV=1 - [EFP2_CHLTR] | 26.00 | 60.53 | 2 | 94 | 21.5 | 5.08 |
| G4NME9 | V-type sodium ATP synthase subunit K OS=Chlamydia trachomatis serovar A (strain A2497) GN=CTO_0326 PE=4 SV=1 - [G4NME9_CHLT4] | 25.66 | 91.22 | 1 | 19 | 15.2 | 8.31 |
| Q3KLH9 | 50S ribosomal protein L14 OS=Chlamydia trachomatis serovar A (strain ATCC VR-571B / DSM 19440 / HAR-13) GN=rplN PE=3 SV=1 - [RL14_CHLTA] | 25.17 | 82.79 | 1 | 89 | 13.4 | 9.80 |
| A0A0E9CH94 | Chaperone protein DnaJ OS=Chlamydia trachomatis GN=dnaJ PE=3 SV=1 - [A0A0E9CH94_CHLTH] | 25.16 | 73.21 | 1 | 146 | 41.9 | 7.62 |
| A0A0E9D3Q4 | Outer membrane protein OS=Chlamydia trachomatis GN=ompH PE=4 SV=1 - [A0A0E9D3Q4_CHLTH] | 23.56 | 63.58 | 1 | 394 | 19.4 | 4.88 |
| A0A0E9F7I6 | Sensor histidine kinase DcuS OS=Chlamydia trachomatis GN=dcuS_2 PE=4 SV=1 - [A0A0E9F7I6_CHLTH] | 23.55 | 58.23 | 1 | 138 | 55.8 | 5.11 |
| A0A0E9C8R9 | 50S ribosomal protein L18 OS=Chlamydia trachomatis GN=rplR PE=3 SV=1 - [A0A0E9C8R9_CHLTH] | 23.34 | 90.24 | 1 | 86 | 13.4 | 10.32 |
| O84294 | Deoxyuridine 5'-triphosphate nucleotidohydrolase OS=Chlamydia trachomatis (strain D/UW-3/Cx) GN=dut PE=3 SV=1 - [DUT_CHLTR] | 23.20 | 77.24 | 2 | 21 | 15.3 | 5.22 |
| A0A0E9FTP1 | Cation transporter E1-E2 family ATPase OS=Chlamydia trachomatis GN=zosA PE=3 SV=1 - [A0A0E9FTP1_CHLTH] | 23.17 | 47.76 | 1 | 205 | 45.7 | 5.29 |
| A0A0E9AYD3 | Proline dipeptidase OS=Chlamydia trachomatis GN=pepP PE=4 SV=1 - [A0A0E9AYD3_CHLTH] | 22.83 | 51.40 | 1 | 102 | 39.3 | 5.59 |
| A0A0H2X383 | Uncharacterized protein OS=Chlamydia trachomatis serovar A (strain ATCC VR-571B / DSM 19440 / HAR-13) GN=CTA_0913 PE=4 SV=1 - [A0A0H2X383_CHLTA] | 22.63 | 55.62 | 3 | 290 | 76.3 | 6.14 |
| A0A0E9ATU0 | Oligoendopeptidase F OS=Chlamydia trachomatis GN=pepF PE=4 SV=1 - [A0A0E9ATU0_CHLTH] | 21.79 | 49.01 | 2 | 90 | 69.0 | 5.95 |
| A0A0E9B0S2 | DNA-invertase hin OS=Chlamydia trachomatis GN=hin_2 PE=4 SV=1 - [A0A0E9B0S2_CHLTH] | 21.77 | 87.21 | 1 | 564 | 60.5 | 9.22 |
| A0A0E9G967 | Translation initiation factor IF-3 OS=Chlamydia trachomatis GN=infC_3 PE=3 SV=1 - [A0A0E9G967_CHLTH] | 21.75 | 78.47 | 1 | 237 | 23.5 | 9.51 |
| Q3KM44 | 50S ribosomal protein L1 OS=Chlamydia trachomatis serovar A (strain ATCC VR-571B / DSM 19440 / HAR-13) GN=rplA PE=3 SV=1 - [RL1_CHLTA] | 21.39 | 78.45 | 1 | 131 | 24.7 | 8.92 |
| O84220 | 5'-nucleotidase SurE OS=Chlamydia trachomatis (strain D/UW-3/Cx) GN=surE PE=3 SV=2 - [SURE_CHLTR] | 21.34 | 51.24 | 5 | 36 | 31.5 | 5.05 |
| G4NPE4 | 50S ribosomal protein L4 OS=Chlamydia trachomatis serovar A (strain A2497) GN=rplD PE=3 SV=1 - [G4NPE4_CHLT4] | 21.31 | 96.10 | 1 | 171 | 25.6 | 9.82 |
| A0A0E9FZA9 | Lon protease OS=Chlamydia trachomatis GN=lon1 PE=2 SV=1 - [A0A0E9FZA9_CHLTH] | 21.07 | 73.50 | 1 | 475 | 91.9 | 7.39 |
| A0A0E9DTU0 | 2,3-bisphosphoglycerate-dependent phosphoglycerate mutase OS=Chlamydia trachomatis GN=gpmA_1 PE=3 SV=1 - [A0A0E9DTU0_CHLTH] | 20.37 | 61.66 | 1 | 133 | 29.0 | 6.07 |
| A0A0E9C3X0 | Succinyl-CoA synthetase subunit alpha OS=Chlamydia trachomatis GN=sucD PE=3 SV=1 - [A0A0E9C3X0_CHLTH] | 20.34 | 58.76 | 2 | 60 | 30.2 | 5.31 |
| A0A0E9CWD9 | Malonyl CoA-acyl carrier protein transacylase OS=Chlamydia trachomatis GN=fabD PE=3 SV=1 - [A0A0E9CWD9_CHLTH] | 20.17 | 41.88 | 2 | 133 | 33.5 | 4.92 |
| A0A0E9CZ71 | Dihydrolipoamide succinyltransferase OS=Chlamydia trachomatis GN=sucB_2 PE=3 SV=1 - [A0A0E9CZ71_CHLTH] | 20.16 | 66.58 | 1 | 98 | 40.3 | 5.31 |
| A0A0E9CFX5 | HAD superfamily hydrolase/phosphatase OS=Chlamydia trachomatis GN=ERS075185_00712 PE=4 SV=1 - [A0A0E9CFX5_CHLTH] | 19.99 | 64.77 | 3 | 37 | 34.2 | 5.22 |
| A0A0E9ATY1 | Putative cytosolic protein OS=Chlamydia trachomatis GN=ERS066953_00424 PE=4 SV=1 - [A0A0E9ATY1_CHLTH] | 19.78 | 61.59 | 1 | 48 | 18.4 | 4.61 |
| A0A0E9CKN6 | MAC/perforin family protein OS=Chlamydia trachomatis GN=ERS075193_00650 PE=4 SV=1 - [A0A0E9CKN6_CHLTH] | 19.72 | 49.14 | 1 | 209 | 90.8 | 6.47 |
| G4NM37 | Uncharacterized protein OS=Chlamydia trachomatis serovar A (strain A2497) GN=CTO_0071 PE=4 SV=1 - [G4NM37_CHLT4] | 19.54 | 85.64 | 1 | 192 | 20.6 | 10.07 |
| Q3KL01 | Phosphoglycerate kinase OS=Chlamydia trachomatis serovar A (strain ATCC VR-571B / DSM 19440 / HAR-13) GN=pgk PE=3 SV=1 - [PGK_CHLTA] | 18.98 | 52.61 | 1 | 199 | 43.0 | 6.00 |
| O84600 | Thio:disulfide Interchange Protein OS=Chlamydia trachomatis (strain D/UW-3/Cx) GN=dsbD PE=4 SV=1 - [O84600_CHLTR] | 18.85 | 37.72 | 1 | 121 | 76.1 | 6.73 |
| A0A0E9CNE8 | Anti-sigma factor antagonist OS=Chlamydia trachomatis GN=rbsV PE=3 SV=1 - [A0A0E9CNE8_CHLTH] | 17.45 | 97.27 | 1 | 128 | 12.4 | 7.85 |
| G4NMX4 | MYG1 protein OS=Chlamydia trachomatis serovar A (strain A2497) GN=CTO_0421 PE=4 SV=1 - [G4NMX4_CHLT4] | 17.28 | 43.09 | 1 | 166 | 34.9 | 6.05 |
| A0A0E9EEQ7 | Peptidoglycan associated lipoprotein OS=Chlamydia trachomatis GN=pal PE=3 SV=1 - [A0A0E9EEQ7_CHLTH] | 17.19 | 47.03 | 1 | 54 | 23.1 | 8.65 |
| A0A0E9FSX3 | DNA polymerase I OS=Chlamydia trachomatis GN=polA PE=4 SV=1 - [A0A0E9FSX3_CHLTH] | 16.59 | 75.36 | 1 | 145 | 8.0 | 6.28 |
| G4NM46 | DNA polymerase III subunit beta OS=Chlamydia trachomatis serovar A (strain A2497) GN=CTO_0080 PE=4 SV=1 - [G4NM46_CHLT4] | 16.43 | 71.26 | 2 | 118 | 47.9 | 6.42 |
| A0A0E9CBL1 | Phosphoglucosamine mutase OS=Chlamydia trachomatis GN=glmM PE=3 SV=1 - [A0A0E9CBL1_CHLTH] | 16.38 | 74.89 | 1 | 122 | 49.4 | 6.48 |
| A0A0E9CFY7 | 3-ketoacyl-ACP reductase OS=Chlamydia trachomatis GN=fabG PE=3 SV=1 - [A0A0E9CFY7_CHLTH] | 16.09 | 71.37 | 2 | 39 | 26.0 | 7.93 |
| A0A0E9CFX6 | Protein RsbW OS=Chlamydia trachomatis GN=btrW PE=4 SV=1 - [A0A0E9CFX6_CHLTH] | 16.02 | 69.86 | 1 | 41 | 16.4 | 6.16 |
| G4NNY3 | Methionine--tRNA ligase OS=Chlamydia trachomatis serovar A (strain A2497) GN=metG PE=3 SV=1 - [G4NNY3_CHLT4] | 16.01 | 63.64 | 2 | 115 | 62.7 | 5.25 |
| A0A0E9FZH4 | Protein of uncharacterised function (DUF1207) OS=Chlamydia trachomatis GN=ERS066954_00213 PE=4 SV=1 - [A0A0E9FZH4_CHLTH] | 15.96 | 30.96 | 2 | 50 | 46.9 | 5.82 |
| Q3KKK4 | 50S ribosomal protein L20 OS=Chlamydia trachomatis serovar A (strain ATCC VR-571B / DSM 19440 / HAR-13) GN=rplT PE=3 SV=1 - [RL20_CHLTA] | 15.04 | 90.24 | 1 | 146 | 13.9 | 11.91 |
| K0G1P9 | Uncharacterized protein OS=Chlamydia trachomatis GN=CT214 PE=4 SV=1 - [K0G1P9_CHLTH] | 15.00 | 44.16 | 1 | 223 | 60.1 | 8.92 |
| K0G334 | Uncharacterized protein OS=Chlamydia trachomatis GN=CT288 PE=4 SV=1 - [K0G334_CHLTH] | 14.82 | 50.44 | 1 | 238 | 63.1 | 8.59 |
| A0A0E9DFS3 | Integrase DNA binding domain OS=Chlamydia trachomatis GN=ERS082928_02137 PE=4 SV=1 - [A0A0E9DFS3_CHLTH] | 14.59 | 59.56 | 1 | 250 | 108.2 | 8.79 |
| A0A0E9CCI4 | Dihydrolipoyl dehydrogenase OS=Chlamydia trachomatis GN=pdhD PE=4 SV=1 - [A0A0E9CCI4_CHLTH] | 14.41 | 62.37 | 1 | 135 | 49.4 | 6.80 |
| A0A0E9G272 | Thioredoxin OS=Chlamydia trachomatis GN=trxA PE=4 SV=1 - [A0A0E9G272_CHLTH] | 14.21 | 100.00 | 1 | 16 | 7.7 | 6.54 |
| A0A0E9ATV2 | Putative metallo-phosphoesterase OS=Chlamydia trachomatis GN=ERS066954_00132 PE=4 SV=1 - [A0A0E9ATV2_CHLTH] | 14.20 | 50.15 | 1 | 69 | 36.5 | 9.10 |
| G4NND5 | 6-phosphogluconolactonase OS=Chlamydia trachomatis serovar A (strain A2497) GN=CTO_0204 PE=4 SV=1 - [G4NND5_CHLT4] | 14.17 | 63.36 | 1 | 69 | 29.8 | 5.40 |
| A0A0E9DPU3 | Protein of uncharacterised function (DUF2709) OS=Chlamydia trachomatis GN=ERS066953_00763 PE=4 SV=1 - [A0A0E9DPU3_CHLTH] | 13.96 | 71.43 | 1 | 112 | 27.5 | 5.39 |
| O84820 | Uncharacterized protein OS=Chlamydia trachomatis (strain D/UW-3/Cx) GN=CT_814 PE=4 SV=1 - [O84820_CHLTR] | 13.95 | 96.24 | 1 | 100 | 15.6 | 10.80 |
| O84640 | Protein CT_635 OS=Chlamydia trachomatis (strain D/UW-3/Cx) GN=CT_635 PE=3 SV=1 - [Y635_CHLTR] | 13.48 | 100.00 | 1 | 127 | 16.8 | 6.58 |
| B0BBG8 | Thymidylate kinase OS=Chlamydia trachomatis serovar L2b (strain UCH-1/proctitis) GN=tmk PE=3 SV=1 - [KTHY_CHLTB] | 13.26 | 56.65 | 2 | 35 | 22.4 | 7.03 |
| Q3KLH4 | 50S ribosomal protein L22 OS=Chlamydia trachomatis serovar A (strain ATCC VR-571B / DSM 19440 / HAR-13) GN=rplV PE=3 SV=1 - [RL22_CHLTA] | 13.04 | 98.20 | 1 | 113 | 12.4 | 11.34 |
| A0A0E9D067 | SH3 domain-containing protein OS=Chlamydia trachomatis GN=ERS075185_00625 PE=4 SV=1 - [A0A0E9D067_CHLTH] | 12.89 | 71.14 | 1 | 146 | 48.5 | 7.74 |
| A0A0E9GA42 | DNA topoisomerase 1 OS=Chlamydia trachomatis GN=topA_5 PE=3 SV=1 - [A0A0E9GA42_CHLTH] | 12.55 | 88.38 | 1 | 612 | 79.2 | 8.98 |
| A0A0E9ASF7 | Putative lipoprotein OS=Chlamydia trachomatis GN=ERS066953_00128 PE=4 SV=1 - [A0A0E9ASF7_CHLTH] | 12.52 | 46.05 | 1 | 33 | 24.0 | 8.98 |
| A0A0E9C683 | Insulinase family metalloprotease OS=Chlamydia trachomatis GN=ERS075185_00552 PE=3 SV=1 - [A0A0E9C683_CHLTH] | 12.35 | 53.29 | 2 | 224 | 109.3 | 5.44 |
| B7SBR8 | DppF OS=Chlamydia trachomatis GN=dppF PE=3 SV=1 - [B7SBR8_CHLTH] | 12.30 | 63.04 | 1 | 59 | 31.0 | 9.54 |
| A0A0H2X0H9 | Uncharacterized protein OS=Chlamydia trachomatis serovar A (strain ATCC VR-571B / DSM 19440 / HAR-13) GN=CTA_0012 PE=4 SV=1 - [A0A0H2X0H9_CHLTA] | 12.20 | 70.33 | 1 | 197 | 48.0 | 7.61 |
| G4NPB1 | Protease IV OS=Chlamydia trachomatis serovar A (strain A2497) GN=CTO_0541 PE=4 SV=1 - [G4NPB1_CHLT4] | 11.63 | 47.40 | 2 | 158 | 37.5 | 8.47 |
| Q3KLQ4 | Glutamate--tRNA ligase OS=Chlamydia trachomatis serovar A (strain ATCC VR-571B / DSM 19440 / HAR-13) GN=gltX PE=3 SV=1 - [SYE_CHLTA] | 11.44 | 64.82 | 1 | 164 | 58.5 | 6.57 |
| B1Q047 | Major outer membrane porin (Fragment) OS=Chlamydia trachomatis GN=omp1 PE=3 SV=1 - [B1Q047_CHLTH] | 10.94 | 34.10 | 1 | 21 | 28.3 | 4.79 |
| A0A0E9D018 | Glycerol-3-phosphate dehydrogenase [NAD(P)+] OS=Chlamydia trachomatis GN=gpsA PE=3 SV=1 - [A0A0E9D018_CHLTH] | 10.94 | 26.65 | 1 | 61 | 36.1 | 7.81 |
| A0A0E9B1N6 | Replicative DNA helicase OS=Chlamydia trachomatis GN=dnaB_2 PE=4 SV=1 - [A0A0E9B1N6_CHLTH] | 10.84 | 59.17 | 1 | 133 | 67.2 | 5.31 |
| A0A0E9DDT9 | Fructose-bisphosphate aldolase OS=Chlamydia trachomatis GN=fba_1 PE=4 SV=1 - [A0A0E9DDT9_CHLTH] | 10.72 | 100.00 | 1 | 58 | 10.3 | 8.43 |
| A0A0E9CPU5 | Putative outer membrane protein OS=Chlamydia trachomatis GN=ERS075195_00212 PE=4 SV=1 - [A0A0E9CPU5_CHLTH] | 10.65 | 70.93 | 1 | 178 | 32.6 | 9.10 |
| B0B7F9 | UDP-3-O-acylglucosamine N-acyltransferase OS=Chlamydia trachomatis serovar L2 (strain 434/Bu / ATCC VR-902B) GN=lpxD PE=3 SV=1 - [LPXD_CHLT2] | 10.55 | 39.55 | 1 | 93 | 38.4 | 7.69 |
| A0A0E9C884 | AMP nucleosidase OS=Chlamydia trachomatis GN=amn PE=4 SV=1 - [A0A0E9C884_CHLTH] | 10.42 | 42.21 | 1 | 41 | 32.0 | 7.05 |
| B0B9C6 | Isoleucine--tRNA ligase OS=Chlamydia trachomatis serovar L2 (strain 434/Bu / ATCC VR-902B) GN=ileS PE=3 SV=1 - [SYI_CHLT2] | 9.89 | 45.95 | 1 | 690 | 118.7 | 5.53 |
| Q3KLT4 | 50S ribosomal protein L27 OS=Chlamydia trachomatis serovar A (strain ATCC VR-571B / DSM 19440 / HAR-13) GN=rpmA PE=3 SV=1 - [RL27_CHLTA] | 9.70 | 97.59 | 1 | 35 | 8.9 | 11.03 |
| G4NM49 | Bifunctional protein FolD OS=Chlamydia trachomatis serovar A (strain A2497) GN=folD PE=3 SV=1 - [G4NM49_CHLT4] | 9.32 | 55.71 | 2 | 124 | 31.1 | 7.58 |
| A0A0E9B1G0 | MutT/Nudix family protein OS=Chlamydia trachomatis GN=ERS066953_01175 PE=3 SV=1 - [A0A0E9B1G0_CHLTH] | 9.24 | 57.33 | 1 | 37 | 17.4 | 5.12 |
| A0A0E9FST2 | Cysteine--tRNA ligase OS=Chlamydia trachomatis GN=cysS_1 PE=3 SV=1 - [A0A0E9FST2_CHLTH] | 9.21 | 45.60 | 1 | 112 | 57.5 | 6.46 |
| A0A0E9CES6 | Methyltransferase OS=Chlamydia trachomatis GN=rsmD PE=4 SV=1 - [A0A0E9CES6_CHLTH] | 9.02 | 49.47 | 2 | 63 | 20.9 | 8.44 |
| A0A0E9G6Y0 | GTPase Obg OS=Chlamydia trachomatis GN=obg_2 PE=3 SV=1 - [A0A0E9G6Y0_CHLTH] | 8.80 | 64.90 | 1 | 225 | 47.8 | 5.27 |
| A0A0H2X2J0 | Uncharacterized protein OS=Chlamydia trachomatis serovar A (strain ATCC VR-571B / DSM 19440 / HAR-13) GN=CTA_0578 PE=4 SV=1 - [A0A0H2X2J0_CHLTA] | 8.71 | 71.48 | 1 | 135 | 31.0 | 9.04 |
| B0B7M2 | V-type ATP synthase subunit D OS=Chlamydia trachomatis serovar L2 (strain 434/Bu / ATCC VR-902B) GN=atpD PE=3 SV=1 - [VATD_CHLT2] | 8.50 | 70.94 | 1 | 165 | 23.2 | 8.98 |
| A0A0H2X241 | Fumarate hydratase OS=Chlamydia trachomatis serovar A (strain ATCC VR-571B / DSM 19440 / HAR-13) GN=fumC PE=4 SV=1 - [A0A0H2X241_CHLTA] | 8.46 | 39.74 | 1 | 195 | 50.3 | 6.52 |
| A0A0E9DXH9 | Domain of Uncharacterised Function (DUF349) OS=Chlamydia trachomatis GN=ERS133246_02573 PE=4 SV=1 - [A0A0E9DXH9_CHLTH] | 8.34 | 67.39 | 1 | 199 | 51.8 | 6.01 |
| A0A0E9ATW3 | Long chain fatty acid--ACP ligase OS=Chlamydia trachomatis GN=aas PE=4 SV=1 - [A0A0E9ATW3_CHLTH] | 7.95 | 40.04 | 1 | 134 | 59.4 | 7.40 |
| A0A0E9FIX0 | Uncharacterized conserved protein OS=Chlamydia trachomatis GN=ERS095036_15907 PE=4 SV=1 - [A0A0E9FIX0_CHLTH] | 7.78 | 60.00 | 1 | 19 | 8.6 | 6.57 |
| A0A0E9DER9 | Chlamydia protein associating with death domains OS=Chlamydia trachomatis GN=ERS075193_00154 PE=4 SV=1 - [A0A0E9DER9_CHLTH] | 7.45 | 48.23 | 1 | 56 | 26.1 | 5.05 |
| A0A0E9C7D8 | Type III secretion system protein OS=Chlamydia trachomatis GN=fliF PE=4 SV=1 - [A0A0E9C7D8_CHLTH] | 7.27 | 49.70 | 1 | 121 | 37.1 | 6.98 |
| A0A0E9DI70 | 1-deoxy-D-xylulose 5-phosphate reductoisomerase OS=Chlamydia trachomatis GN=dxr PE=3 SV=1 - [A0A0E9DI70_CHLTH] | 7.19 | 46.97 | 1 | 314 | 41.7 | 6.06 |
| O84463 | Probable transcriptional regulatory protein CT_457 OS=Chlamydia trachomatis (strain D/UW-3/Cx) GN=CT_457 PE=3 SV=1 - [Y457_CHLTR] | 7.11 | 70.59 | 1 | 173 | 26.5 | 5.90 |
| A0A0E9C5V1 | Aromatic amino acid aminotransferase OS=Chlamydia trachomatis GN=aspC_1 PE=3 SV=1 - [A0A0E9C5V1_CHLTH] | 6.98 | 63.00 | 1 | 63 | 44.6 | 5.60 |
| A0A0E9CTJ7 | 50S ribosomal protein L15 OS=Chlamydia trachomatis GN=rplO PE=3 SV=1 - [A0A0E9CTJ7_CHLTH] | 6.81 | 68.67 | 1 | 75 | 18.6 | 10.23 |
| G4NPL7 | Glycine--tRNA ligase OS=Chlamydia trachomatis serovar A (strain A2497) GN=CTO_0867 PE=3 SV=1 - [G4NPL7_CHLT4] | 6.45 | 39.96 | 1 | 173 | 115.9 | 6.42 |
| A0A0H2X242 | HflX OS=Chlamydia trachomatis serovar A (strain ATCC VR-571B / DSM 19440 / HAR-13) GN=hlfX PE=4 SV=1 - [A0A0H2X242_CHLTA] | 6.44 | 70.47 | 1 | 161 | 50.8 | 7.42 |
| G4NNL4 | Histidine kinase OS=Chlamydia trachomatis serovar A (strain A2497) GN=CTO_0511 PE=4 SV=1 - [G4NNL4_CHLT4] | 6.20 | 76.14 | 1 | 55 | 39.8 | 5.88 |
| A0A0E9D121 | SwiB complex protein OS=Chlamydia trachomatis GN=ERS075185_00164 PE=4 SV=1 - [A0A0E9D121_CHLTH] | 6.17 | 94.19 | 1 | 117 | 9.7 | 9.70 |
| B0B925 | Glycogen synthase OS=Chlamydia trachomatis serovar L2 (strain 434/Bu / ATCC VR-902B) GN=glgA PE=3 SV=1 - [GLGA_CHLT2] | 5.31 | 46.62 | 1 | 75 | 53.4 | 5.94 |
| A0A0E9GJ50 | Tryptophanyl-tRNA synthetase OS=Chlamydia trachomatis GN=trpS_6 PE=4 SV=1 - [A0A0E9GJ50_CHLTH] | 5.22 | 100.00 | 1 | 37 | 8.7 | 7.25 |
| A0A0E9EK24 | VanZ like family OS=Chlamydia trachomatis GN=ERS095036_05267 PE=4 SV=1 - [A0A0E9EK24_CHLTH] | 4.90 | 56.40 | 1 | 25 | 19.8 | 8.92 |
| B0RK54 | Major outer membrane porin (Fragment) OS=Chlamydia trachomatis GN=omp1 PE=3 SV=1 - [B0RK54_CHLTH] | 4.90 | 34.95 | 1 | 152 | 33.1 | 5.30 |
| A0A0E9D3A0 | Uncharacterised protein OS=Chlamydia trachomatis GN=ERS075185_00879 PE=4 SV=1 - [A0A0E9D3A0_CHLTH] | 4.48 | 44.39 | 1 | 60 | 21.9 | 5.59 |
| O84859 | Methionine aminopeptidase OS=Chlamydia trachomatis (strain D/UW-3/Cx) GN=map PE=3 SV=1 - [MAP1_CHLTR] | 4.45 | 54.30 | 1 | 50 | 32.6 | 6.51 |
| K0GAB8 | Uncharacterized protein OS=Chlamydia trachomatis GN=CT229 PE=4 SV=1 - [K0GAB8_CHLTH] | 3.20 | 63.26 | 1 | 82 | 23.4 | 8.38 |
| O84309 | V-type ATP synthase beta chain OS=Chlamydia trachomatis (strain D/UW-3/Cx) GN=atpB PE=3 SV=1 - [VATB_CHLTR] | 2.81 | 47.72 | 1 | 187 | 48.7 | 5.99 |

**Table E2.** PEAKS DB identification of 239 CtB protein hits with FDR of 5% (on peptide level) and 0% (on protein level). Abbreviations: Cov. = protein sequence coverage, # Uniq. Pep. = number of unique peptides # Pep. = number of peptides

| **Accession** | **Description** | **Score**  **(-10lgP)** | **Cov**  **[%]** | **# Pep.** | **# Uniq.**  **Pep** | **MW (kDa)** |
| --- | --- | --- | --- | --- | --- | --- |
| G4NM84\|G4NM84_CHLT4 | 60 kDa chaperonin OS=Chlamydia trachomatis serovar A (strain A2497) GN=groL PE=3 SV=1 | 361.4 | 82 | 73 | 4 | 58.1 |
| sp\|Q3KMQ9\|CH60_CHLTA | 60 kDa chaperonin OS=Chlamydia trachomatis serovar A (strain ATCC VR-571B / DSM 19440 / HAR-13) GN=groL PE=2 SV=3 | 361.4 | 82 | 73 | 4 | 58.1 |
| G4NMP7\|G4NMP7_CHLT4 | Thioredoxin peroxidase OS=Chlamydia trachomatis serovar A (strain A2497) GN=CTO_0654 PE=4 SV=1 | 268.5 | 70 | 17 | 17 | 22.7 |
| A0A0H2X1C0\|A0A0H2X1C0_CHLTA | Thioredoxin peroxidase OS=Chlamydia trachomatis serovar A (strain ATCC VR-571B / DSM 19440 / HAR-13) GN=ahpC PE=4 SV=1 | 268.5 | 70 | 17 | 17 | 22.7 |
| B6EE12\|B6EE12_CHLTH | Major outer membrane porin (Fragment) OS=Chlamydia trachomatis GN=omp1 PE=3 SV=1 | 263.8 | 64 | 59 | 2 | 39.4 |
| sp\|Q3KMX6\|AMPA_CHLTA | Probable cytosol aminopeptidase OS=Chlamydia trachomatis serovar A (strain ATCC VR-571B / DSM 19440 / HAR-13) GN=pepA PE=3 SV=1 | 263.4 | 43 | 17 | 3 | 54.1 |
| A0A0E9DIU3\|A0A0E9DIU3_CHLTH | Gag gene protein p24 (Core nucleocapsid protein) OS=Chlamydia trachomatis GN=ERS082929_01031 PE=4 SV=1 | 260.6 | 34 | 19 | 4 | 65.7 |
| A0A0E9CT34\|A0A0E9CT34_CHLTH | Elongation factor Tu OS=Chlamydia trachomatis GN=tuf PE=3 SV=1 | 260.0 | 81 | 39 | 3 | 43.3 |
| sp\|Q3KM40\|EFTU_CHLTA | Elongation factor Tu OS=Chlamydia trachomatis serovar A (strain ATCC VR-571B / DSM 19440 / HAR-13) GN=tuf PE=3 SV=1 | 260.0 | 81 | 39 | 3 | 43.3 |
| Q2TV41\|Q2TV41_CHLTH | Polymorphic membrane protein D OS=Chlamydia trachomatis GN=pmpD PE=4 SV=1 | 254.0 | 18 | 25 | 2 | 160.7 |
| A1X3B2\|A1X3B2_CHLTH | 30S ribosomal protein S2 OS=Chlamydia trachomatis GN=rs2 PE=3 SV=1 | 243.3 | 53 | 14 | 2 | 30.9 |
| A0A0H2X1N0\|A0A0H2X1N0_CHLTA | SctN OS=Chlamydia trachomatis serovar A (strain ATCC VR-571B / DSM 19440 / HAR-13) GN=sctN PE=4 SV=1 | 242.0 | 42 | 16 | 2 | 48.2 |
| G4NN56\|G4NN56_CHLT4 | SctN OS=Chlamydia trachomatis serovar A (strain A2497) GN=CTO_0726 PE=4 SV=1 | 242.0 | 42 | 16 | 2 | 48.2 |
| A0A0H2X2P3\|A0A0H2X2P3_CHLTA | Glyceraldehyde 3-phosphate dehydrogenase OS=Chlamydia trachomatis serovar A (strain ATCC VR-571B / DSM 19440 / HAR-13) GN=gapA PE=4 SV=1 | 221.7 | 45 | 14 | 2 | 36.3 |
| A0A0E9AUE0\|A0A0E9AUE0_CHLTH | Uncharacterised protein OS=Chlamydia trachomatis GN=ERS066953_01368 PE=4 SV=1 | 213.1 | 68 | 10 | 10 | 21.1 |
| sp\|O84588\|Y584_CHLTR | Protein CT_584 OS=Chlamydia trachomatis (strain D/UW-3/Cx) GN=CT_584 PE=3 SV=1 | 213.1 | 68 | 10 | 10 | 21.1 |
| A0A0H2X1F6\|A0A0H2X1F6_CHLTA | Uncharacterized protein OS=Chlamydia trachomatis serovar A (strain ATCC VR-571B / DSM 19440 / HAR-13) GN=CTA_0634 PE=4 SV=1 | 213.1 | 68 | 10 | 10 | 21.1 |
| M9UIQ4\|M9UIQ4_CHLTH | Uncharacterized protein OS=Chlamydia trachomatis L2/434/Bu(f) GN=CTLFINAL_04420 PE=4 SV=1 | 213.1 | 68 | 10 | 10 | 21.1 |
| G4NMM5\|G4NMM5_CHLT4 | Uncharacterized protein OS=Chlamydia trachomatis serovar A (strain A2497) GN=CTO_0634 PE=4 SV=1 | 213.1 | 68 | 10 | 10 | 21.1 |
| A0A0H3MCR9\|A0A0H3MCR9_CHLT2 | Uncharacterized protein OS=Chlamydia trachomatis serovar L2 (strain 434/Bu / ATCC VR-902B) GN=CTL0847 PE=4 SV=1 | 213.1 | 68 | 10 | 10 | 21.1 |
| K0GGT0\|K0GGT0_CHLTH | Ribosome-recycling factor OS=Chlamydia trachomatis GN=frr PE=3 SV=1 | 208.6 | 55 | 10 | 4 | 20.1 |
| G4NN64\|G4NN64_CHLT4 | Ribosome-recycling factor OS=Chlamydia trachomatis serovar A (strain A2497) GN=frr PE=3 SV=1 | 208.6 | 55 | 10 | 4 | 20.1 |
| A0A0H2X292\|A0A0H2X292_CHLTA | Anti-sigma F factor antagonist OS=Chlamydia trachomatis serovar A (strain ATCC VR-571B / DSM 19440 / HAR-13) GN=rsbV PE=4 SV=1 | 206.6 | 58 | 6 | 6 | 12.5 |
| M9UM43\|M9UM43_CHLTH | Anti-sigma factor antagonist OS=Chlamydia trachomatis L2/434/Bu(f) GN=CTLFINAL_03560 PE=3 SV=1 | 206.6 | 58 | 6 | 6 | 12.5 |
| O84431\|O84431_CHLTR | Anti-sigma factor antagonist OS=Chlamydia trachomatis (strain D/UW-3/Cx) GN=rsbV_1 PE=3 SV=1 | 206.6 | 58 | 6 | 6 | 12.5 |
| A0A0E9CCK9\|A0A0E9CCK9_CHLTH | Transcription termination/antitermination protein NusG OS=Chlamydia trachomatis GN=nusG PE=3 SV=1 | 205.7 | 54 | 8 | 2 | 20.8 |
| A0A0H3MCF1\|A0A0H3MCF1_CHLT2 | Uncharacterized protein OS=Chlamydia trachomatis serovar L2 (strain 434/Bu / ATCC VR-902B) GN=CTL0028 PE=4 SV=1 | 204.7 | 88 | 10 | 10 | 8.8 |
| A0A0E9D2L0\|A0A0E9D2L0_CHLTH | UPF0109 protein ERS066953_00249 OS=Chlamydia trachomatis GN=ERS066953_00249 PE=3 SV=1 | 204.7 | 88 | 10 | 10 | 8.8 |
| M9UG13\|M9UG13_CHLTH | UPF0109 protein CTLFINAL_00155 OS=Chlamydia trachomatis L2/434/Bu(f) GN=CTLFINAL_00155 PE=3 SV=1 | 204.7 | 88 | 10 | 10 | 8.8 |
| A0A0H2X2Z8\|A0A0H2X2Z8_CHLTA | RNA binding protein OS=Chlamydia trachomatis serovar A (strain ATCC VR-571B / DSM 19440 / HAR-13) GN=CTA_0716 PE=4 SV=1 | 204.7 | 88 | 10 | 10 | 8.8 |
| sp\|O84666\|Y659_CHLTR | UPF0109 protein CT_659 OS=Chlamydia trachomatis (strain D/UW-3/Cx) GN=CT_659 PE=3 SV=1 | 204.7 | 88 | 10 | 10 | 8.8 |
| Q83TT3\|Q83TT3_CHLTH | Major outer membrane protein OS=Chlamydia trachomatis GN=porB PE=4 SV=1 | 200.2 | 26 | 8 | 2 | 37.3 |
| sp\|B0B899\|RL2_CHLT2 | 50S ribosomal protein L2 OS=Chlamydia trachomatis serovar L2 (strain 434/Bu / ATCC VR-902B) GN=rplB PE=3 SV=1 | 193.3 | 31 | 10 | 8 | 31.5 |
| sp\|O84530\|RL2_CHLTR | 50S ribosomal protein L2 OS=Chlamydia trachomatis (strain D/UW-3/Cx) GN=rplB PE=3 SV=1 | 193.3 | 31 | 10 | 8 | 31.5 |
| A0A0E9AXH1\|A0A0E9AXH1_CHLTH | 50S ribosomal protein L2 OS=Chlamydia trachomatis GN=rplB PE=3 SV=1 | 193.3 | 31 | 10 | 8 | 31.5 |
| G4NNS7\|G4NNS7_CHLT4 | 6 7-dimethyl-8-ribityllumazine synthase OS=Chlamydia trachomatis serovar A (strain A2497) GN=ribH PE=3 SV=1 | 191.7 | 41 | 7 | 7 | 17.2 |
| sp\|O84805\|RL25_CHLTR | 50S ribosomal protein L25 OS=Chlamydia trachomatis (strain D/UW-3/Cx) GN=rplY PE=3 SV=1 | 189.7 | 64 | 10 | 10 | 20.4 |
| sp\|O84217\|ALF1_CHLTR | Probable fructose-bisphosphate aldolase class 1 OS=Chlamydia trachomatis (strain D/UW-3/Cx) GN=fbaB PE=3 SV=1 | 185.3 | 45 | 13 | 3 | 38.0 |
| sp\|O84007\|GATB_CHLTR | Aspartyl/glutamyl-tRNA(Asn/Gln) amidotransferase subunit B OS=Chlamydia trachomatis (strain D/UW-3/Cx) GN=gatB PE=3 SV=1 | 181.4 | 17 | 5 | 5 | 55.0 |
| M9UE49\|M9UE49_CHLTH | Aspartyl/glutamyl-tRNA(Asn/Gln) amidotransferase subunit B OS=Chlamydia trachomatis L2/434/Bu(f) GN=gatB PE=3 SV=1 | 181.4 | 17 | 5 | 5 | 55.0 |
| A0A0E9CFY0\|A0A0E9CFY0_CHLTH | 30S ribosomal protein S1 OS=Chlamydia trachomatis GN=rpsA PE=4 SV=1 | 177.9 | 20 | 14 | 14 | 63.6 |
| sp\|O84100\|RS1_CHLTR | 30S ribosomal protein S1 OS=Chlamydia trachomatis (strain D/UW-3/Cx) GN=rpsA PE=3 SV=1 | 177.9 | 20 | 14 | 14 | 63.6 |
| sp\|B0B7S2\|DEF_CHLT2 | Peptide deformylase OS=Chlamydia trachomatis serovar L2 (strain 434/Bu / ATCC VR-902B) GN=def PE=3 SV=1 | 177.1 | 49 | 8 | 7 | 20.5 |
| sp\|Q3KM05\|DEF_CHLTA | Peptide deformylase OS=Chlamydia trachomatis serovar A (strain ATCC VR-571B / DSM 19440 / HAR-13) GN=def PE=3 SV=1 | 177.1 | 49 | 8 | 7 | 20.5 |
| G4NLX2\|G4NLX2_CHLT4 | DegP OS=Chlamydia trachomatis serovar A (strain A2497) GN=CTO_0897 PE=4 SV=1 | 169.7 | 12 | 6 | 2 | 53.3 |
| sp\|O84031\|RL19_CHLTR | 50S ribosomal protein L19 OS=Chlamydia trachomatis (strain D/UW-3/Cx) GN=rplS PE=3 SV=1 | 164.9 | 49 | 8 | 8 | 13.1 |
| sp\|B0BB14\|RL19_CHLTB | 50S ribosomal protein L19 OS=Chlamydia trachomatis serovar L2b (strain UCH-1/proctitis) GN=rplS PE=3 SV=1 | 164.9 | 49 | 8 | 8 | 13.1 |
| A0A0E9CXI8\|A0A0E9CXI8_CHLTH | Malate dehydrogenase OS=Chlamydia trachomatis GN=mdh PE=3 SV=1 | 162.9 | 26 | 9 | 2 | 35.7 |
| sp\|O84381\|MDH_CHLTR | Malate dehydrogenase OS=Chlamydia trachomatis (strain D/UW-3/Cx) GN=mdh PE=3 SV=1 | 162.9 | 26 | 9 | 2 | 35.7 |
| A0A0E9CQH4\|A0A0E9CQH4_CHLTH | Zn binding protein OS=Chlamydia trachomatis GN=ERS075193_00385 PE=4 SV=1 | 157.3 | 24 | 8 | 2 | 29.6 |
| G4NMY6\|G4NMY6_CHLT4 | Zn binding protein OS=Chlamydia trachomatis serovar A (strain A2497) GN=CTO_0433 PE=4 SV=1 | 157.3 | 24 | 8 | 2 | 29.6 |
| A0A0H2X145\|A0A0H2X145_CHLTA | Zn binding protein OS=Chlamydia trachomatis serovar A (strain ATCC VR-571B / DSM 19440 / HAR-13) GN=CTA_0433 PE=4 SV=1 | 157.3 | 24 | 8 | 2 | 29.6 |
| A0A0E9F3I9\|A0A0E9F3I9_CHLTH | ATP synthase subunit beta OS=Chlamydia trachomatis GN=sctN_4 PE=3 SV=1 | 153.7 | 11 | 4 | 3 | 50.7 |
| sp\|B0BC75\|RS7_CHLTB | 30S ribosomal protein S7 OS=Chlamydia trachomatis serovar L2b (strain UCH-1/proctitis) GN=rpsG PE=3 SV=1 | 153.2 | 36 | 6 | 6 | 17.8 |
| G4NNI2\|G4NNI2_CHLT4 | 30S ribosomal protein S7 OS=Chlamydia trachomatis serovar A (strain A2497) GN=rpsG PE=3 SV=1 | 153.2 | 36 | 6 | 6 | 17.8 |
| A0A0H2X1S7\|A0A0H2X1S7_CHLTA | HAD superfamily hydrolase/phosphatase OS=Chlamydia trachomatis serovar A (strain ATCC VR-571B / DSM 19440 / HAR-13) GN=CTA_0109 PE=4 SV=1 | 152.9 | 19 | 4 | 4 | 34.3 |
| A0A0E9CFX5\|A0A0E9CFX5_CHLTH | HAD superfamily hydrolase/phosphatase OS=Chlamydia trachomatis GN=ERS075185_00712 PE=4 SV=1 | 152.9 | 19 | 4 | 4 | 34.3 |
| A0A0H3MLD3\|A0A0H3MLD3_CHLT2 | SSU ribosomal protein S5P OS=Chlamydia trachomatis serovar L2 (strain 434/Bu / ATCC VR-902B) GN=rpsE PE=4 SV=1 | 151.5 | 30 | 7 | 7 | 17.8 |
| sp\|Q3KLI5\|RS5_CHLTA | 30S ribosomal protein S5 OS=Chlamydia trachomatis serovar A (strain ATCC VR-571B / DSM 19440 / HAR-13) GN=rpsE PE=3 SV=1 | 151.5 | 30 | 7 | 7 | 17.8 |
| A0A0E9D3E4\|A0A0E9D3E4_CHLTH | 30S ribosomal protein S5 OS=Chlamydia trachomatis GN=rpsE PE=3 SV=1 | 151.5 | 30 | 7 | 7 | 17.8 |
| M9UIB3\|M9UIB3_CHLTH | 30S ribosomal protein S5 OS=Chlamydia trachomatis L2/434/Bu(f) GN=rpsE PE=3 SV=1 | 151.5 | 30 | 7 | 7 | 17.8 |
| G4NNG0\|G4NNG0_CHLT4 | 50S ribosomal protein L21 OS=Chlamydia trachomatis serovar A (strain A2497) GN=rplU PE=3 SV=1 | 151.5 | 50 | 5 | 5 | 12.3 |
| sp\|Q3KLH6\|RL16_CHLTA | 50S ribosomal protein L16 OS=Chlamydia trachomatis serovar A (strain ATCC VR-571B / DSM 19440 / HAR-13) GN=rplP PE=3 SV=1 | 148.6 | 25 | 3 | 3 | 15.8 |
| sp\|B0BCG0\|RL16_CHLTB | 50S ribosomal protein L16 OS=Chlamydia trachomatis serovar L2b (strain UCH-1/proctitis) GN=rplP PE=3 SV=1 | 148.6 | 25 | 3 | 3 | 15.8 |
| A0A0E9CFD6\|A0A0E9CFD6_CHLTH | 50S ribosomal protein L16 OS=Chlamydia trachomatis GN=rplP_1 PE=3 SV=1 | 148.6 | 25 | 3 | 3 | 15.8 |
| Q6LCD5\|Q6LCD5_CHLTH | 10 kDa chaperonin (Fragment) OS=Chlamydia trachomatis GN=hypA PE=3 SV=1 | 148.0 | 64 | 9 | 9 | 10.7 |
| A0A0E9CAW6\|A0A0E9CAW6_CHLTH | 10 kDa chaperonin OS=Chlamydia trachomatis GN=groES PE=3 SV=1 | 148.0 | 61 | 9 | 9 | 11.2 |
| A0A0E9G3M0\|A0A0E9G3M0_CHLTH | Transporter OS=Chlamydia trachomatis GN=ERS066954_00215 PE=4 SV=1 | 143.0 | 14 | 7 | 7 | 77.1 |
| A0A0H3MH86\|A0A0H3MH86_CHLT2 | Uncharacterized protein OS=Chlamydia trachomatis serovar L2 (strain 434/Bu / ATCC VR-902B) GN=CTL0643 PE=4 SV=1 | 143.0 | 14 | 7 | 7 | 77.1 |
| M9UEF8\|M9UEF8_CHLTH | Low calcium response locus protein D OS=Chlamydia trachomatis L2/434/Bu(f) GN=CTLFINAL_01820 PE=4 SV=1 | 143.0 | 19 | 10 | 10 | 78.0 |
| G4NM61\|G4NM61_CHLT4 | Type III secretion inner membrane protein SctV OS=Chlamydia trachomatis serovar A (strain A2497) GN=CTO_0095 PE=4 SV=1 | 143.0 | 19 | 10 | 10 | 78.0 |
| O84092\|O84092_CHLTR | Low Calcium Response D OS=Chlamydia trachomatis (strain D/UW-3/Cx) GN=lcrD PE=4 SV=1 | 143.0 | 19 | 10 | 10 | 78.0 |
| A0A0H3MCY4\|A0A0H3MCY4_CHLT2 | Low calcium response protein D (Predicted to be part of the TTSS apparatus) OS=Chlamydia trachomatis serovar L2 (strain 434/Bu / ATCC VR-902B) GN=lcrD PE=4 SV=1 | 143.0 | 19 | 10 | 10 | 78.0 |
| A0A0H2X0L8\|A0A0H2X0L8_CHLTA | Low calcium response protein D OS=Chlamydia trachomatis serovar A (strain ATCC VR-571B / DSM 19440 / HAR-13) GN=lcrD PE=4 SV=1 | 143.0 | 19 | 10 | 10 | 78.0 |
| A0A0E9DLE6\|A0A0E9DLE6_CHLTH | Putative membrane transport protein OS=Chlamydia trachomatis GN=invA PE=4 SV=1 | 143.0 | 19 | 10 | 10 | 78.0 |
| A0A0E9CZH0\|A0A0E9CZH0_CHLTH | Putative membrane transport protein OS=Chlamydia trachomatis GN=invA PE=4 SV=1 | 143.0 | 19 | 10 | 10 | 78.0 |
| A0A0H2X1D2\|A0A0H2X1D2_CHLTA | Methyltransferase OS=Chlamydia trachomatis serovar A (strain ATCC VR-571B / DSM 19440 / HAR-13) GN=yhhF PE=4 SV=1 | 140.8 | 26 | 3 | 3 | 20.9 |
| O84494\|O84494_CHLTR | Methylase OS=Chlamydia trachomatis (strain D/UW-3/Cx) GN=yhhF PE=4 SV=1 | 140.8 | 26 | 3 | 3 | 20.9 |
| A0A0H3MDP4\|A0A0H3MDP4_CHLT2 | Methyltransferase OS=Chlamydia trachomatis serovar L2 (strain 434/Bu / ATCC VR-902B) GN=CTL0748 PE=4 SV=1 | 140.8 | 26 | 3 | 3 | 20.9 |
| sp\|Q3KKP0\|RS6_CHLTA | 30S ribosomal protein S6 OS=Chlamydia trachomatis serovar A (strain ATCC VR-571B / DSM 19440 / HAR-13) GN=rpsF PE=3 SV=1 | 139.2 | 35 | 5 | 4 | 12.9 |
| sp\|B0B928\|RS6_CHLT2 | 30S ribosomal protein S6 OS=Chlamydia trachomatis serovar L2 (strain 434/Bu / ATCC VR-902B) GN=rpsF PE=3 SV=1 | 139.2 | 35 | 5 | 4 | 12.9 |
| A0A0E9DS10\|A0A0E9DS10_CHLTH | ABC transporter ATPase OS=Chlamydia trachomatis GN=dppF PE=3 SV=1 | 134.2 | 21 | 5 | 3 | 31.0 |
| O84412\|O84412_CHLTR | DnaK Suppressor OS=Chlamydia trachomatis (strain D/UW-3/Cx) GN=dksA PE=4 SV=1 | 129.8 | 54 | 6 | 6 | 13.9 |
| A0A0H3MCC6\|A0A0H3MCC6_CHLT2 | DnaK suppressor protein OS=Chlamydia trachomatis serovar L2 (strain 434/Bu / ATCC VR-902B) GN=dksA PE=4 SV=1 | 129.8 | 54 | 6 | 6 | 13.9 |
| A0A0E9FXT1\|A0A0E9FXT1_CHLTH | DnaK suppressor protein OS=Chlamydia trachomatis GN=yocK PE=4 SV=1 | 129.8 | 54 | 6 | 6 | 13.9 |
| M9UHY5\|M9UHY5_CHLTH | Molecular chaperone DnaK OS=Chlamydia trachomatis L2/434/Bu(f) GN=CTLFINAL_03465 PE=4 SV=2 | 129.8 | 50 | 6 | 6 | 15.3 |
| A0A0E9C6W0\|A0A0E9C6W0_CHLTH | Trigger factor OS=Chlamydia trachomatis GN=tig PE=3 SV=1 | 127.8 | 12 | 4 | 4 | 50.1 |
| sp\|Q3KKY7\|TIG_CHLTA | Trigger factor OS=Chlamydia trachomatis serovar A (strain ATCC VR-571B / DSM 19440 / HAR-13) GN=tig PE=3 SV=1 | 127.8 | 12 | 4 | 4 | 50.1 |
| M9UHS2\|M9UHS2_CHLTH | Deoxyuridine 5'-triphosphate nucleotidohydrolase OS=Chlamydia trachomatis L2/434/Bu(f) GN=dut PE=3 SV=1 | 127.5 | 37 | 3 | 3 | 15.3 |
| A0A0E9DSY4\|A0A0E9DSY4_CHLTH | Deoxyuridine 5'-triphosphate nucleotidohydrolase OS=Chlamydia trachomatis GN=dut_2 PE=3 SV=1 | 127.5 | 37 | 3 | 3 | 15.3 |
| sp\|B0BBS3\|DUT_CHLTB | Deoxyuridine 5'-triphosphate nucleotidohydrolase OS=Chlamydia trachomatis serovar L2b (strain UCH-1/proctitis) GN=dut PE=3 SV=1 | 127.5 | 37 | 3 | 3 | 15.3 |
| sp\|O84377\|AAXA_CHLTR | Porin AaxA OS=Chlamydia trachomatis (strain D/UW-3/Cx) GN=aaxA PE=3 SV=2 | 125.5 | 20 | 8 | 8 | 51.5 |
| sp\|Q3KLY4\|AAXA_CHLTA | Porin AaxA OS=Chlamydia trachomatis serovar A (strain ATCC VR-571B / DSM 19440 / HAR-13) GN=aaxA PE=3 SV=2 | 125.5 | 20 | 8 | 8 | 51.5 |
| A0A0E9CU42\|A0A0E9CU42_CHLTH | Carbohydrate-selective porin%2C OprB family protein OS=Chlamydia trachomatis GN=aaxA PE=4 SV=1 | 125.5 | 20 | 8 | 8 | 51.5 |
| G4NMV6\|G4NMV6_CHLT4 | Uncharacterized protein OS=Chlamydia trachomatis serovar A (strain A2497) GN=CTO_0404 PE=4 SV=1 | 125.5 | 20 | 8 | 8 | 52.0 |
| sp\|B0B930\|RL9_CHLT2 | 50S ribosomal protein L9 OS=Chlamydia trachomatis serovar L2 (strain 434/Bu / ATCC VR-902B) GN=rplI PE=3 SV=1 | 125.2 | 43 | 5 | 5 | 18.4 |
| M9ULS5\|M9ULS5_CHLTH | 50S ribosomal protein L9 OS=Chlamydia trachomatis L2/434/Bu(f) GN=rplI PE=3 SV=1 | 125.2 | 43 | 5 | 5 | 18.4 |
| G4NPM5\|G4NPM5_CHLT4 | 50S ribosomal protein L9 OS=Chlamydia trachomatis serovar A (strain A2497) GN=rplI PE=3 SV=1 | 125.2 | 43 | 5 | 5 | 18.4 |
| sp\|Q3KLH4\|RL22_CHLTA | 50S ribosomal protein L22 OS=Chlamydia trachomatis serovar A (strain ATCC VR-571B / DSM 19440 / HAR-13) GN=rplV PE=3 SV=1 | 124.7 | 42 | 5 | 3 | 12.5 |
| A0A0E9CJJ3\|A0A0E9CJJ3_CHLTH | 50S ribosomal protein L22 OS=Chlamydia trachomatis GN=rplV_1 PE=3 SV=1 | 124.7 | 42 | 5 | 3 | 12.5 |
| G4NP91\|G4NP91_CHLT4 | Integration host factor beta-subunit OS=Chlamydia trachomatis serovar A (strain A2497) GN=CTO_0289 PE=3 SV=1 | 124.5 | 27 | 4 | 4 | 17.0 |
| M9UF21\|M9UF21_CHLTH | Integration host factor OS=Chlamydia trachomatis L2/434/Bu(f) GN=CTLFINAL_04880 PE=3 SV=2 | 124.5 | 27 | 4 | 4 | 17.0 |
| A0A0H2X182\|A0A0H2X182_CHLTA | Hypothetical membrane associated protein OS=Chlamydia trachomatis serovar A (strain ATCC VR-571B / DSM 19440 / HAR-13) GN=CTA_0002 PE=4 SV=1 | 122.7 | 40 | 2 | 2 | 9.8 |
| A0A0H3MCS7\|A0A0H3MCS7_CHLT2 | Putative membrane protein OS=Chlamydia trachomatis serovar L2 (strain 434/Bu / ATCC VR-902B) GN=CTL0256 PE=4 SV=1 | 122.7 | 40 | 2 | 2 | 9.8 |
| M9UKV6\|M9UKV6_CHLTH | Membrane protein OS=Chlamydia trachomatis L2/434/Bu(f) GN=CTLFINAL_01355 PE=4 SV=1 | 122.7 | 40 | 2 | 2 | 9.8 |
| G4NNU9\|G4NNU9_CHLT4 | Putative membrane associated protein OS=Chlamydia trachomatis serovar A (strain A2497) GN=CTO_0002 PE=4 SV=1 | 122.7 | 40 | 2 | 2 | 9.8 |
| M9UM80\|M9UM80_CHLTH | Type III secretion system chaperone OS=Chlamydia trachomatis L2/434/Bu(f) GN=CTLFINAL_01575 PE=4 SV=1 | 121.9 | 38 | 6 | 6 | 18.4 |
| A0A0H3MBJ5\|A0A0H3MBJ5_CHLT2 | Putative type III secretion system chaperone OS=Chlamydia trachomatis serovar L2 (strain 434/Bu / ATCC VR-902B) GN=CTL0299 PE=4 SV=1 | 121.9 | 38 | 6 | 6 | 18.4 |
| G4NNK4\|G4NNK4_CHLT4 | Probable transcriptional regulatory protein CTO_0499 OS=Chlamydia trachomatis serovar A (strain A2497) GN=CTO_0499 PE=3 SV=1 | 120.0 | 10 | 2 | 2 | 26.5 |
| sp\|B0B830\|Y717_CHLT2 | Probable transcriptional regulatory protein CTL0717 OS=Chlamydia trachomatis serovar L2 (strain 434/Bu / ATCC VR-902B) GN=CTL0717 PE=3 SV=1 | 120.0 | 10 | 2 | 2 | 26.5 |
| A0A0E9EDI2\|A0A0E9EDI2_CHLTH | Serine--tRNA ligase OS=Chlamydia trachomatis GN=serS_1 PE=3 SV=1 | 118.6 | 16 | 5 | 5 | 48.4 |
| sp\|Q3KKW5\|SYS_CHLTA | Serine--tRNA ligase OS=Chlamydia trachomatis serovar A (strain ATCC VR-571B / DSM 19440 / HAR-13) GN=serS PE=3 SV=1 | 118.6 | 16 | 5 | 5 | 48.3 |
| G4NPH1\|G4NPH1_CHLT4 | Elongation factor P OS=Chlamydia trachomatis serovar A (strain A2497) GN=efp PE=3 SV=1 | 117.5 | 28 | 5 | 3 | 21.5 |
| sp\|O84757\|EFP2_CHLTR | Elongation factor P 2 OS=Chlamydia trachomatis (strain D/UW-3/Cx) GN=efp2 PE=3 SV=1 | 117.5 | 28 | 5 | 3 | 21.5 |
| A0A0H2X366\|A0A0H2X366_CHLTA | Translation elongation factor P OS=Chlamydia trachomatis serovar A (strain ATCC VR-571B / DSM 19440 / HAR-13) GN=efp_2 PE=4 SV=1 | 117.5 | 28 | 5 | 3 | 21.5 |
| G4NPM8\|G4NPM8_CHLT4 | Insulin-degrading enzyme OS=Chlamydia trachomatis serovar A (strain A2497) GN=CTO_0878 PE=4 SV=1 | 115.3 | 3 | 3 | 2 | 108.4 |
| O84776\|O84776_CHLTR | Hydrolase/phosphatase homolog OS=Chlamydia trachomatis (strain D/UW-3/Cx) GN=CT_771 PE=3 SV=1 | 114.0 | 25 | 3 | 3 | 17.4 |
| A0A0H2X385\|A0A0H2X385_CHLTA | Phosphohydrolase (MutT/nudix family protein) OS=Chlamydia trachomatis serovar A (strain ATCC VR-571B / DSM 19440 / HAR-13) GN=CTA_0841 PE=4 SV=1 | 114.0 | 25 | 3 | 3 | 17.4 |
| G4NPJ0\|G4NPJ0_CHLT4 | Phosphohydrolase (MutT/nudix family protein) OS=Chlamydia trachomatis serovar A (strain A2497) GN=CTO_0841 PE=4 SV=1 | 114.0 | 25 | 3 | 3 | 17.4 |
| A0A0E9DK93\|A0A0E9DK93_CHLTH | MutT/Nudix family protein OS=Chlamydia trachomatis GN=ERS075193_00895 PE=3 SV=1 | 114.0 | 25 | 3 | 3 | 17.4 |
| sp\|Q3KMZ0\|SYM_CHLTA | Methionine--tRNA ligase OS=Chlamydia trachomatis serovar A (strain ATCC VR-571B / DSM 19440 / HAR-13) GN=metG PE=3 SV=1 | 113.6 | 12 | 5 | 5 | 62.7 |
| A0A0E9DB98\|A0A0E9DB98_CHLTH | Methionine--tRNA ligase OS=Chlamydia trachomatis GN=metG_1 PE=3 SV=1 | 113.6 | 12 | 5 | 5 | 62.7 |
| A0A0E9D4E7\|A0A0E9D4E7_CHLTH | Glutamate--tRNA ligase OS=Chlamydia trachomatis GN=gltX PE=3 SV=1 | 112.9 | 7 | 3 | 3 | 58.6 |
| G4NNJ1\|G4NNJ1_CHLT4 | Glutamate--tRNA ligase OS=Chlamydia trachomatis serovar A (strain A2497) GN=gltX PE=3 SV=1 | 112.9 | 7 | 3 | 3 | 58.5 |
| M9UI50\|M9UI50_CHLTH | Metallophosphoesterase OS=Chlamydia trachomatis L2/434/Bu(f) GN=CTLFINAL_03765 PE=4 SV=1 | 110.8 | 17 | 4 | 4 | 37.1 |
| sp\|Q3KKY8\|CLPP2_CHLTA | ATP-dependent Clp protease proteolytic subunit 2 OS=Chlamydia trachomatis serovar A (strain ATCC VR-571B / DSM 19440 / HAR-13) GN=clpP2 PE=3 SV=1 | 108.1 | 26 | 4 | 4 | 22.0 |
| M9ULH7\|M9ULH7_CHLTH | ATP-dependent Clp protease proteolytic subunit OS=Chlamydia trachomatis L2/434/Bu(f) GN=clpP PE=3 SV=1 | 108.1 | 26 | 4 | 4 | 22.0 |
| A0A0E9C8U7\|A0A0E9C8U7_CHLTH | Ribonuclease E OS=Chlamydia trachomatis GN=rng PE=4 SV=1 | 108.1 | 14 | 5 | 5 | 59.4 |
| A0A0H2X210\|A0A0H2X210_CHLTA | Ribonuclease E OS=Chlamydia trachomatis serovar A (strain ATCC VR-571B / DSM 19440 / HAR-13) GN=cafE PE=4 SV=1 | 108.1 | 14 | 5 | 5 | 59.3 |
| M9UKL6\|M9UKL6_CHLTH | Ribonuclease G OS=Chlamydia trachomatis L2/434/Bu(f) GN=CTLFINAL_00950 PE=4 SV=1 | 108.1 | 14 | 5 | 5 | 59.4 |
| A0A0H3MBC8\|A0A0H3MBC8_CHLT2 | Ribonuclease E OS=Chlamydia trachomatis serovar L2 (strain 434/Bu / ATCC VR-902B) GN=cafE PE=4 SV=1 | 108.1 | 14 | 5 | 5 | 59.4 |
| O84814\|O84814_CHLTR | Axial Filament Protein OS=Chlamydia trachomatis (strain D/UW-3/Cx) GN=cafE PE=4 SV=1 | 108.1 | 14 | 5 | 5 | 59.3 |
| A0A0E9DHL4\|A0A0E9DHL4_CHLTH | Ribonuclease E OS=Chlamydia trachomatis GN=rng PE=4 SV=1 | 108.1 | 14 | 5 | 5 | 59.3 |
| G4NPN0\|G4NPN0_CHLT4 | Ribonuclease E OS=Chlamydia trachomatis serovar A (strain A2497) GN=CTO_0880 PE=4 SV=1 | 108.1 | 14 | 5 | 5 | 60.9 |
| A0A0E9CMC9\|A0A0E9CMC9_CHLTH | Elongation factor P OS=Chlamydia trachomatis GN=efp-2 PE=3 SV=1 | 108.1 | 24 | 3 | 3 | 19.5 |
| A0A0H2X1G3\|A0A0H2X1G3_CHLTA | Translation elongation factor P OS=Chlamydia trachomatis serovar A (strain ATCC VR-571B / DSM 19440 / HAR-13) GN=efp_1 PE=4 SV=1 | 108.1 | 23 | 3 | 3 | 20.5 |
| sp\|O84124\|EFP1_CHLTR | Elongation factor P 1 OS=Chlamydia trachomatis (strain D/UW-3/Cx) GN=efp1 PE=3 SV=1 | 108.1 | 23 | 3 | 3 | 20.5 |
| A0A0E9D1J8\|A0A0E9D1J8_CHLTH | Protein RsbW OS=Chlamydia trachomatis GN=btrW PE=4 SV=1 | 107.7 | 41 | 5 | 5 | 16.4 |
| O84553\|O84553_CHLTR | Sigma regulatory factor-histidine kinase OS=Chlamydia trachomatis (strain D/UW-3/Cx) GN=rsbW PE=4 SV=1 | 107.7 | 41 | 5 | 5 | 16.4 |
| A0A0E9CFX6\|A0A0E9CFX6_CHLTH | Protein RsbW OS=Chlamydia trachomatis GN=btrW PE=4 SV=1 | 107.7 | 41 | 5 | 5 | 16.4 |
| M9UMJ4\|M9UMJ4_CHLTH | Histidine kinase OS=Chlamydia trachomatis L2/434/Bu(f) GN=CTLFINAL_04235 PE=4 SV=1 | 107.7 | 41 | 5 | 5 | 16.4 |
| A0A0H3MLE8\|A0A0H3MLE8_CHLT2 | Sigma regulatory factor-histidine kinase OS=Chlamydia trachomatis serovar L2 (strain 434/Bu / ATCC VR-902B) GN=rsbW PE=4 SV=1 | 107.7 | 41 | 5 | 5 | 16.4 |
| G4NPG9\|G4NPG9_CHLT4 | RsbW OS=Chlamydia trachomatis serovar A (strain A2497) GN=CTO_0599 PE=4 SV=1 | 107.7 | 39 | 5 | 5 | 17.3 |
| A0A0H3MGR8\|A0A0H3MGR8_CHLT2 | Biotin carboxyl carrier protein of acetyl-CoA carboxylase OS=Chlamydia trachomatis serovar L2 (strain 434/Bu / ATCC VR-902B) GN=accB PE=4 SV=1 | 107.6 | 34 | 3 | 3 | 18.2 |
| A0A0E9D783\|A0A0E9D783_CHLTH | Acetyl-CoA carboxylase biotin carboxyl carrier protein subunit OS=Chlamydia trachomatis GN=accB PE=4 SV=1 | 107.6 | 34 | 3 | 3 | 18.2 |
| A0A0E9FWY4\|A0A0E9FWY4_CHLTH | MotA/TolQ/ExbB proton channel family protein OS=Chlamydia trachomatis GN=exbB PE=3 SV=1 | 107.2 | 14 | 2 | 2 | 25.9 |
| M9UFX8\|M9UFX8_CHLTH | Dihydrolipoyl dehydrogenase OS=Chlamydia trachomatis L2/434/Bu(f) GN=CTLFINAL_04275 PE=4 SV=1 | 105.3 | 12 | 5 | 5 | 49.5 |
| A0A0H2X2R2\|A0A0H2X2R2_CHLTA | Dihydrolipoamide dehydrogenase OS=Chlamydia trachomatis serovar A (strain ATCC VR-571B / DSM 19440 / HAR-13) GN=lpdA PE=4 SV=1 | 105.3 | 12 | 5 | 5 | 49.5 |
| A0A0E9CS98\|A0A0E9CS98_CHLTH | Dihydrolipoyl dehydrogenase OS=Chlamydia trachomatis GN=pdhD PE=4 SV=1 | 105.3 | 12 | 5 | 5 | 49.5 |
| A0A0H3MHJ2\|A0A0H3MHJ2_CHLT2 | Dihydrolipoamide dehydrogenase OS=Chlamydia trachomatis serovar L2 (strain 434/Bu / ATCC VR-902B) GN=lpdA PE=4 SV=1 | 105.3 | 12 | 5 | 5 | 49.5 |
| A0A0E9CCI4\|A0A0E9CCI4_CHLTH | Dihydrolipoyl dehydrogenase OS=Chlamydia trachomatis GN=pdhD PE=4 SV=1 | 105.3 | 12 | 5 | 5 | 49.5 |
| M9UI72\|M9UI72_CHLTH | Oxidoreductase OS=Chlamydia trachomatis L2/434/Bu(f) GN=CTLFINAL_03620 PE=4 SV=1 | 102.1 | 11 | 4 | 4 | 39.9 |
| A0A0H2X2I2\|A0A0H2X2I2_CHLTA | NADPH-cytochrome P450 reductase OS=Chlamydia trachomatis serovar A (strain ATCC VR-571B / DSM 19440 / HAR-13) GN=cysJ PE=4 SV=1 | 102.1 | 11 | 4 | 4 | 39.9 |
| G4NNH9\|G4NNH9_CHLT4 | NADPH-cytochrome P450 reductase OS=Chlamydia trachomatis serovar A (strain A2497) GN=CTO_0475 PE=4 SV=1 | 102.1 | 11 | 4 | 4 | 39.9 |
| O84442\|O84442_CHLTR | Sulfite Reductase OS=Chlamydia trachomatis (strain D/UW-3/Cx) GN=cysJ PE=4 SV=1 | 102.1 | 11 | 4 | 4 | 39.9 |
| sp\|B0B9I6\|FOLD_CHLT2 | Bifunctional protein FolD OS=Chlamydia trachomatis serovar L2 (strain 434/Bu / ATCC VR-902B) GN=folD PE=3 SV=1 | 101.2 | 23 | 4 | 4 | 30.9 |
| sp\|B0BB65\|FOLD_CHLTB | Bifunctional protein FolD OS=Chlamydia trachomatis serovar L2b (strain UCH-1/proctitis) GN=folD PE=3 SV=1 | 101.2 | 23 | 4 | 4 | 30.9 |
| A0A0H3MCJ3\|A0A0H3MCJ3_CHLT2 | Anti-sigma F factor antagonist OS=Chlamydia trachomatis serovar L2 (strain 434/Bu / ATCC VR-902B) GN=rbsV PE=4 SV=1 | 98.5 | 16 | 2 | 2 | 12.4 |
| O84770\|O84770_CHLTR | Anti-sigma factor antagonist OS=Chlamydia trachomatis (strain D/UW-3/Cx) GN=rsbV_2 PE=3 SV=1 | 98.5 | 16 | 2 | 2 | 12.4 |
| sp\|O84586\|PARA_CHLTR | ParA family protein CT_582 OS=Chlamydia trachomatis (strain D/UW-3/Cx) GN=CT_582 PE=3 SV=1 | 97.7 | 24 | 4 | 4 | 28.2 |
| A0A0H3MDT7\|A0A0H3MDT7_CHLT2 | Chromosome partitioning ATPase (ParA family) OS=Chlamydia trachomatis serovar L2 (strain 434/Bu / ATCC VR-902B) GN=minD PE=4 SV=1 | 97.7 | 24 | 4 | 4 | 28.2 |
| A0A0E9DFM9\|A0A0E9DFM9_CHLTH | ATPase%2C ParA FAMILY OS=Chlamydia trachomatis GN=soj_1 PE=4 SV=1 | 97.7 | 24 | 4 | 4 | 28.2 |
| A0A0E9D3V4\|A0A0E9D3V4_CHLTH | ATPase%2C ParA FAMILY OS=Chlamydia trachomatis GN=soj_1 PE=4 SV=1 | 97.7 | 24 | 4 | 4 | 28.2 |
| M9UMM8\|M9UMM8_CHLTH | Chromosome partitioning protein ParA OS=Chlamydia trachomatis L2/434/Bu(f) GN=CTLFINAL_04410 PE=4 SV=1 | 97.7 | 24 | 4 | 4 | 28.2 |
| G4NMM3\|G4NMM3_CHLT4 | ATPase ParA FAMILY OS=Chlamydia trachomatis serovar A (strain A2497) GN=CTO_0632 PE=4 SV=1 | 97.7 | 24 | 4 | 4 | 28.6 |
| A0A0E9DV71\|A0A0E9DV71_CHLTH | ATPase%2C ParA FAMILY OS=Chlamydia trachomatis GN=soj_4 PE=4 SV=1 | 97.7 | 24 | 4 | 4 | 28.6 |
| sp\|B0B929\|RS18_CHLT2 | 30S ribosomal protein S18 OS=Chlamydia trachomatis serovar L2 (strain 434/Bu / ATCC VR-902B) GN=rpsR PE=3 SV=1 | 97.2 | 42 | 4 | 4 | 9.4 |
| sp\|Q3KKN9\|RS18_CHLTA | 30S ribosomal protein S18 OS=Chlamydia trachomatis serovar A (strain ATCC VR-571B / DSM 19440 / HAR-13) GN=rpsR PE=3 SV=1 | 97.2 | 42 | 4 | 4 | 9.4 |
| A0A0E9DK62\|A0A0E9DK62_CHLTH | 30S ribosomal protein S18 OS=Chlamydia trachomatis GN=rpsR PE=3 SV=1 | 97.2 | 42 | 4 | 4 | 9.4 |
| A0A0E9DH16\|A0A0E9DH16_CHLTH | GTPase HflX OS=Chlamydia trachomatis GN=hflX PE=3 SV=1 | 94.8 | 5 | 2 | 2 | 50.9 |
| G4NPI2\|G4NPI2_CHLT4 | RNA binding protein OS=Chlamydia trachomatis serovar A (strain A2497) GN=CTO_0833 PE=3 SV=1 | 92.1 | 24 | 3 | 3 | 15.5 |
| A0A0E9CW55\|A0A0E9CW55_CHLTH | Peptidoglycan associated lipoprotein OS=Chlamydia trachomatis GN=pal PE=3 SV=1 | 91.7 | 11 | 2 | 2 | 21.5 |
| M9UNW8\|M9UNW8_CHLTH | Membrane protein OS=Chlamydia trachomatis L2/434/Bu(f) GN=CTLFINAL_04505 PE=3 SV=1 | 91.7 | 11 | 2 | 2 | 21.5 |
| A0A0H2X2P6\|A0A0H2X2P6_CHLTA | Peptidoglycan-associated lipoprotein OS=Chlamydia trachomatis serovar A (strain ATCC VR-571B / DSM 19440 / HAR-13) GN=pal PE=4 SV=1 | 91.7 | 11 | 2 | 2 | 21.5 |
| A0A0H3MDU9\|A0A0H3MDU9_CHLT2 | Peptidoglycan-associated lipoprotein OS=Chlamydia trachomatis serovar L2 (strain 434/Bu / ATCC VR-902B) GN=pal PE=4 SV=1 | 91.7 | 11 | 2 | 2 | 21.5 |
| A0A0H3MB37\|A0A0H3MB37_CHLT2 | Cytochrome d ubiquinol oxidase subunit I OS=Chlamydia trachomatis serovar L2 (strain 434/Bu / ATCC VR-902B) GN=cydA PE=4 SV=1 | 91.2 | 6 | 3 | 2 | 50.2 |
| A0A0H2X0C7\|A0A0H2X0C7_CHLTA | Cytochrome d ubiquinol oxidase subunit I OS=Chlamydia trachomatis serovar A (strain ATCC VR-571B / DSM 19440 / HAR-13) GN=cydA PE=4 SV=1 | 91.2 | 6 | 3 | 2 | 50.2 |
| O84016\|O84016_CHLTR | Cytochrome Oxidase Subunit I OS=Chlamydia trachomatis (strain D/UW-3/Cx) GN=cydA PE=4 SV=1 | 91.2 | 6 | 3 | 2 | 50.2 |
| A0A0E9CQK7\|A0A0E9CQK7_CHLTH | Uncharacterized conserved protein OS=Chlamydia trachomatis GN=ERS075194_01595 PE=4 SV=1 | 90.9 | 26 | 3 | 3 | 18.3 |
| sp\|P66123\|RL27_CHLTR | 50S ribosomal protein L27 OS=Chlamydia trachomatis (strain D/UW-3/Cx) GN=rpmA PE=3 SV=1 | 90.3 | 33 | 2 | 2 | 8.9 |
| A0A0E9C7I7\|A0A0E9C7I7_CHLTH | 50S ribosomal protein L27 OS=Chlamydia trachomatis GN=rpmA PE=3 SV=1 | 90.3 | 33 | 2 | 2 | 8.9 |
| A0A0E9D620\|A0A0E9D620_CHLTH | Cysteine--tRNA ligase OS=Chlamydia trachomatis GN=cysS PE=3 SV=1 | 86.7 | 4 | 2 | 2 | 57.2 |
| A0A0E9DWF3\|A0A0E9DWF3_CHLTH | Pyruvate dehydrogenase E1 component subunit alpha OS=Chlamydia trachomatis GN=pdhA_1 PE=4 SV=1 | 86.2 | 10 | 3 | 2 | 37.2 |
| G4NP68\|G4NP68_CHLT4 | Pyruvate dehydrogenase E1 component subunit alpha OS=Chlamydia trachomatis serovar A (strain A2497) GN=pdhA PE=4 SV=1 | 86.2 | 10 | 3 | 2 | 37.2 |
| A0A0E9DI70\|A0A0E9DI70_CHLTH | 1-deoxy-D-xylulose 5-phosphate reductoisomerase OS=Chlamydia trachomatis GN=dxr PE=3 SV=1 | 85.1 | 6 | 2 | 2 | 41.8 |
| A0A0E9CXM5\|A0A0E9CXM5_CHLTH | 1-deoxy-D-xylulose 5-phosphate reductoisomerase OS=Chlamydia trachomatis GN=dxr PE=3 SV=1 | 85.1 | 6 | 2 | 2 | 41.7 |
| A0A0E9CJH7\|A0A0E9CJH7_CHLTH | Protein of uncharacterised function (DUF2709) OS=Chlamydia trachomatis GN=ERS075193_00230 PE=4 SV=1 | 84.9 | 12 | 3 | 2 | 27.4 |
| Q9S6V3\|Q9S6V3_CHLTH | ADP%2C ATP carrier protein OS=Chlamydia trachomatis GN=npt1 PE=4 SV=1 | 84.6 | 6 | 3 | 3 | 58.1 |
| A0A0H3MGN2\|A0A0H3MGN2_CHLT2 | ADP ATP carrier protein OS=Chlamydia trachomatis serovar L2 (strain 434/Bu / ATCC VR-902B) GN=CTL0321 PE=4 SV=1 | 84.6 | 6 | 3 | 3 | 58.1 |
| M9UEC9\|M9UEC9_CHLTH | ATPase AAA OS=Chlamydia trachomatis L2/434/Bu(f) GN=CTLFINAL_01695 PE=4 SV=1 | 84.6 | 6 | 3 | 3 | 58.1 |
| sp\|O84068\|TLC1_CHLTR | ADP ATP carrier protein 1 OS=Chlamydia trachomatis (strain D/UW-3/Cx) GN=tlcA PE=3 SV=1 | 84.6 | 6 | 3 | 3 | 58.1 |
| O84248\|O84248_CHLTR | Pyruvate Dehydrogenase Beta OS=Chlamydia trachomatis (strain D/UW-3/Cx) GN=pdhB PE=4 SV=1 | 84.4 | 14 | 4 | 4 | 36.2 |
| A0A0E9DSX9\|A0A0E9DSX9_CHLTH | Thymidylate kinase OS=Chlamydia trachomatis GN=tmk_2 PE=3 SV=1 | 83.9 | 17 | 2 | 2 | 22.5 |
| sp\|B0B9T8\|KTHY_CHLT2 | Thymidylate kinase OS=Chlamydia trachomatis serovar L2 (strain 434/Bu / ATCC VR-902B) GN=tmk PE=3 SV=1 | 83.9 | 17 | 2 | 2 | 22.5 |
| sp\|O84804\|GLGA_CHLTR | Glycogen synthase OS=Chlamydia trachomatis (strain D/UW-3/Cx) GN=glgA PE=3 SV=1 | 82.1 | 5 | 2 | 2 | 53.4 |
| A0A0E9CL94\|A0A0E9CL94_CHLTH | Glycogen synthase OS=Chlamydia trachomatis GN=glgA2 PE=3 SV=1 | 82.1 | 5 | 2 | 2 | 53.4 |
| A0A0E9DHF9\|A0A0E9DHF9_CHLTH | Na(+)-translocating NADH-quinone reductase subunit A OS=Chlamydia trachomatis GN=nqrA PE=3 SV=1 | 82.1 | 12 | 3 | 3 | 51.8 |
| sp\|O84639\|NQRA_CHLTR | Na(+)-translocating NADH-quinone reductase subunit A OS=Chlamydia trachomatis (strain D/UW-3/Cx) GN=nqrA PE=3 SV=1 | 82.1 | 12 | 3 | 3 | 51.8 |
| A0A0E9AS89\|A0A0E9AS89_CHLTH | 50S ribosomal protein L28 OS=Chlamydia trachomatis GN=rpmB PE=3 SV=1 | 80.2 | 22 | 3 | 2 | 10.1 |
| sp\|O84088\|RL28_CHLTR | 50S ribosomal protein L28 OS=Chlamydia trachomatis (strain D/UW-3/Cx) GN=rpmB PE=3 SV=1 | 80.2 | 22 | 3 | 2 | 10.1 |
| sp\|B0B9J4\|RL28_CHLT2 | 50S ribosomal protein L28 OS=Chlamydia trachomatis serovar L2 (strain 434/Bu / ATCC VR-902B) GN=rpmB PE=3 SV=1 | 80.2 | 22 | 3 | 2 | 10.1 |
| G4NP38\|G4NP38_CHLT4 | Putative membrane spanning protein OS=Chlamydia trachomatis serovar A (strain A2497) GN=CTO_0241 PE=4 SV=1 | 80.1 | 6 | 2 | 2 | 33.0 |
| A0A0H3MBQ9\|A0A0H3MBQ9_CHLT2 | Putative exported protein OS=Chlamydia trachomatis serovar L2 (strain 434/Bu / ATCC VR-902B) GN=CTL0473 PE=4 SV=1 | 80.1 | 6 | 2 | 2 | 33.1 |
| A0A0E9DL21\|A0A0E9DL21_CHLTH | Putative membrane spanning protein OS=Chlamydia trachomatis GN=ERS066953_00163 PE=4 SV=1 | 80.1 | 6 | 2 | 2 | 33.1 |
| A0A0E9CP53\|A0A0E9CP53_CHLTH | Type III secretion chaperone OS=Chlamydia trachomatis GN=scc1 PE=4 SV=1 | 79.6 | 21 | 3 | 3 | 16.2 |
| A0A0H3MHE0\|A0A0H3MHE0_CHLT2 | 1-acyl-sn-glycerol-3-phosphate acyltransferase OS=Chlamydia trachomatis serovar L2 (strain 434/Bu / ATCC VR-902B) GN=plsC PE=4 SV=1 | 79.0 | 13 | 3 | 2 | 23.8 |
| A0A0E9DER9\|A0A0E9DER9_CHLTH | Chlamydia protein associating with death domains OS=Chlamydia trachomatis GN=ERS075193_00154 PE=4 SV=1 | 78.5 | 19 | 3 | 3 | 26.1 |
| M9UIP8\|M9UIP8_CHLTH | Dehydrogenase OS=Chlamydia trachomatis L2/434/Bu(f) GN=CTLFINAL_04565 PE=4 SV=1 | 78.5 | 18 | 3 | 3 | 26.8 |
| sp\|O84616\|PQQCL_CHLTR | PqqC-like protein OS=Chlamydia trachomatis (strain D/UW-3/Cx) GN=CT_610 PE=1 SV=1 | 78.5 | 18 | 3 | 3 | 26.7 |
| G4NMQ5\|G4NMQ5_CHLT4 | Chlamydia protein associating with death domains OS=Chlamydia trachomatis serovar A (strain A2497) GN=CTO_0663 PE=4 SV=1 | 78.5 | 18 | 3 | 3 | 27.5 |
| G4NPH0\|G4NPH0_CHLT4 | AMP nucleosidase OS=Chlamydia trachomatis serovar A (strain A2497) GN=CTO_0820 PE=4 SV=1 | 78.4 | 9 | 2 | 2 | 32.0 |
| A0A0H2X1U4\|A0A0H2X1U4_CHLTA | AMP nucleosidase OS=Chlamydia trachomatis serovar A (strain ATCC VR-571B / DSM 19440 / HAR-13) GN=amn PE=4 SV=1 | 78.4 | 9 | 2 | 2 | 32.0 |
| A0A0E9CTJ7\|A0A0E9CTJ7_CHLTH | 50S ribosomal protein L15 OS=Chlamydia trachomatis GN=rplO PE=3 SV=1 | 77.5 | 18 | 2 | 2 | 18.6 |
| G4NND5\|G4NND5_CHLT4 | 6-phosphogluconolactonase OS=Chlamydia trachomatis serovar A (strain A2497) GN=CTO_0204 PE=4 SV=1 | 76.7 | 8 | 2 | 2 | 29.8 |
| G4NNX3\|G4NNX3_CHLT4 | 50S ribosomal protein L31 type B OS=Chlamydia trachomatis serovar A (strain A2497) GN=rpmE2 PE=3 SV=1 | 76.7 | 39 | 4 | 4 | 12.2 |
| sp\|Q3KN00\|RL31B_CHLTA | 50S ribosomal protein L31 type B OS=Chlamydia trachomatis serovar A (strain ATCC VR-571B / DSM 19440 / HAR-13) GN=rpmE2 PE=3 SV=1 | 76.7 | 39 | 4 | 4 | 12.2 |
| G4NPJ4\|G4NPJ4_CHLT4 | Acyl-acyl carrier protein synthetase OS=Chlamydia trachomatis serovar A (strain A2497) GN=CTO_0846 PE=4 SV=1 | 76.7 | 8 | 3 | 3 | 59.4 |
| A0A0H2X0W7\|A0A0H2X0W7_CHLTA | Hypothetical secreted protein OS=Chlamydia trachomatis serovar A (strain ATCC VR-571B / DSM 19440 / HAR-13) GN=CTA_0275 PE=4 SV=1 | 74.7 | 12 | 2 | 2 | 24.0 |
| G4NP76\|G4NP76_CHLT4 | Putative secreted protein OS=Chlamydia trachomatis serovar A (strain A2497) GN=CTO_0275 PE=4 SV=1 | 74.7 | 12 | 2 | 2 | 24.0 |
| A0A0E9G4K3\|A0A0E9G4K3_CHLTH | Serine hydroxymethyltransferase OS=Chlamydia trachomatis GN=glyA_2 PE=3 SV=1 | 74.2 | 4 | 3 | 2 | 47.1 |
| G4NN07\|G4NN07_CHLT4 | CpxR OS=Chlamydia trachomatis serovar A (strain A2497) GN=CTO_0683 PE=4 SV=1 | 74.1 | 27 | 4 | 4 | 25.7 |
| A0A0E9AYR5\|A0A0E9AYR5_CHLTH | Phosphoglycerate kinase OS=Chlamydia trachomatis GN=pgk PE=3 SV=1 | 73.8 | 14 | 4 | 4 | 43.1 |
| sp\|B0B8R9\|PGK_CHLT2 | Phosphoglycerate kinase OS=Chlamydia trachomatis serovar L2 (strain 434/Bu / ATCC VR-902B) GN=pgk PE=3 SV=1 | 73.8 | 14 | 4 | 4 | 43.1 |
| A0A0E9FZN2\|A0A0E9FZN2_CHLTH | SH3 domain-containing protein OS=Chlamydia trachomatis GN=ERS066953_00510 PE=4 SV=1 | 72.3 | 5 | 2 | 2 | 48.5 |
| A0A0E9CWX0\|A0A0E9CWX0_CHLTH | 30S ribosomal protein S17 OS=Chlamydia trachomatis GN=rpsQ PE=3 SV=1 | 70.4 | 33 | 3 | 3 | 9.6 |
| G4NPD6\|G4NPD6_CHLT4 | 30S ribosomal protein S17 OS=Chlamydia trachomatis serovar A (strain A2497) GN=rpsQ PE=3 SV=1 | 70.4 | 33 | 3 | 3 | 9.6 |
| sp\|B0BCF8\|RS17_CHLTB | 30S ribosomal protein S17 OS=Chlamydia trachomatis serovar L2b (strain UCH-1/proctitis) GN=rpsQ PE=3 SV=1 | 70.4 | 33 | 3 | 3 | 9.6 |
| O84569\|O84569_CHLTR | Uncharacterized protein OS=Chlamydia trachomatis (strain D/UW-3/Cx) GN=CT_565 PE=4 SV=1 | 67.4 | 12 | 2 | 2 | 16.0 |
| A0A0E9CXQ1\|A0A0E9CXQ1_CHLTH | Putative inner membrane protein OS=Chlamydia trachomatis GN=ERS075185_00276 PE=4 SV=1 | 67.4 | 12 | 2 | 2 | 16.0 |
| A0A0H3MLF9\|A0A0H3MLF9_CHLT2 | Putative membrane protein OS=Chlamydia trachomatis serovar L2 (strain 434/Bu / ATCC VR-902B) GN=CTL0828 PE=4 SV=1 | 67.4 | 12 | 2 | 2 | 16.0 |
| M9UIM3\|M9UIM3_CHLTH | Membrane protein OS=Chlamydia trachomatis L2/434/Bu(f) GN=CTLFINAL_04320 PE=4 SV=1 | 67.4 | 12 | 2 | 2 | 16.0 |
| A0A0H2X1H7\|A0A0H2X1H7_CHLTA | Uncharacterized protein OS=Chlamydia trachomatis serovar A (strain ATCC VR-571B / DSM 19440 / HAR-13) GN=CTA_0615 PE=4 SV=1 | 67.4 | 12 | 2 | 2 | 16.0 |
| A0A0E9AW84\|A0A0E9AW84_CHLTH | Putative inner membrane protein OS=Chlamydia trachomatis GN=ERS066953_01300 PE=4 SV=1 | 67.4 | 12 | 2 | 2 | 16.0 |
| A0A0E9GFA7\|A0A0E9GFA7_CHLTH | Chaperone protein DnaK OS=Chlamydia trachomatis GN=dnaK_4 PE=2 SV=1 | 65.5 | 2 | 2 | 2 | 68.5 |
| A0A0H3MDT2\|A0A0H3MDT2_CHLT2 | Proline dipeptidase OS=Chlamydia trachomatis serovar L2 (strain 434/Bu / ATCC VR-902B) GN=pepP PE=4 SV=1 | 65.0 | 5 | 2 | 2 | 39.3 |
| M9UII9\|M9UII9_CHLTH | Aminopeptidase OS=Chlamydia trachomatis L2/434/Bu(f) GN=CTLFINAL_04365 PE=4 SV=1 | 65.0 | 5 | 2 | 2 | 39.3 |
| A0A0E9AYD3\|A0A0E9AYD3_CHLTH | Proline dipeptidase OS=Chlamydia trachomatis GN=pepP PE=4 SV=1 | 65.0 | 5 | 2 | 2 | 39.3 |
| sp\|O84537\|FABZ_CHLTR | 3-hydroxyacyl-[acyl-carrier-protein] dehydratase FabZ OS=Chlamydia trachomatis (strain D/UW-3/Cx) GN=fabZ PE=3 SV=1 | 60.1 | 11 | 2 | 2 | 16.6 |
| G4NMX4\|G4NMX4_CHLT4 | MYG1 protein OS=Chlamydia trachomatis serovar A (strain A2497) GN=CTO_0421 PE=4 SV=1 | 54.9 | 9 | 2 | 2 | 35.0 |

**Table E3**. SEQUEST identification of 125 CtB protein hits with peptide FDR of 0.1%. Abbreviations: Cov. = protein sequence coverage, # Uniq. Pep. = number of unique peptides, # PSMs = number of peptide-spectrum matches

| **Accession** | **Description** | **Score** | **Cov.**  **[%]** | **# Uniq.**  **Pep.** | **#**  **PSMs** | **MW**  **[kDa]** | **calc.**  **pI** |
| --- | --- | --- | --- | --- | --- | --- | --- |
| P23421 | Major outer membrane porin, serovar B OS=Chlamydia trachomatis GN=ompA PE=2 SV=1 - [MOMPB_CHLTH] | 1499.9 | 43.7 | 9 | 428 | 42.5 | 5.34 |
| Q6U5G4 | Major outer membrane porin (Fragment) OS=Chlamydia trachomatis GN=omp1 PE=3 SV=1 - [Q6U5G4_CHLTH] | 652.4 | 25.7 | 1 | 206 | 31.2 | 5.10 |
| B0B9L8 | 60 kDa chaperonin OS=Chlamydia trachomatis serovar L2 (strain 434/Bu / ATCC VR-902B) GN=groL PE=3 SV=1 - [CH60_CHLT2] | 553.0 | 55.9 | 19 | 161 | 58.1 | 5.35 |
| A0A0E9F3I9 | ATP synthase subunit beta OS=Chlamydia trachomatis GN=sctN_4 PE=3 SV=1 - [A0A0E9F3I9_CHLTH] | 138.7 | 3.0 | 1 | 35 | 50.7 | 4.94 |
| P26758 | Large cysteine-rich periplasmic protein OmcB, serovar C OS=Chlamydia trachomatis GN=omcB PE=2 SV=1 - [OMCBC_CHLTH] | 130.1 | 22.1 | 7 | 37 | 58.6 | 7.21 |
| A0A0E9CBG7 | Chaperone protein DnaK OS=Chlamydia trachomatis GN=dnaK PE=2 SV=1 - [A0A0E9CBG7_CHLTH] | 117.7 | 26.4 | 8 | 48 | 70.8 | 5.14 |
| A0A0E9ASC3 | Putative membrane associated protein OS=Chlamydia trachomatis GN=ERS066953_00494 PE=4 SV=1 - [A0A0E9ASC3_CHLTH] | 92.1 | 25.6 | 1 | 19 | 9.8 | 8.34 |
| A0A0E9DC36 | Gag gene protein p24 (Core nucleocapsid protein) OS=Chlamydia trachomatis GN=ERS082928_02139 PE=4 SV=1 - [A0A0E9DC36_CHLTH] | 91.5 | 21.3 | 1 | 21 | 31.6 | 8.91 |
| A0A0E9DKJ7 | Gag gene protein p24 (Core nucleocapsid protein) OS=Chlamydia trachomatis GN=ERS082931_00949 PE=4 SV=1 - [A0A0E9DKJ7_CHLTH] | 84.3 | 18.3 | 2 | 20 | 42.4 | 8.84 |
| G4NMM0 | Uncharacterized protein OS=Chlamydia trachomatis serovar A (strain A2497) GN=CTO_0629 PE=4 SV=1 - [G4NMM0_CHLT4] | 80.6 | 19.6 | 4 | 25 | 44 | 9.23 |
| K0G3B3 | CHLPN 76 kD protein-like OS=Chlamydia trachomatis GN=CT623 PE=4 SV=1 - [K0G3B3_CHLTH] | 79.9 | 34.5 | 9 | 22 | 48.3 | 8.19 |
| A0A0E9DYT2 | Manganese-binding protein OS=Chlamydia trachomatis GN=troA_1 PE=3 SV=1 - [A0A0E9DYT2_CHLTH] | 69.7 | 40.5 | 7 | 20 | 37 | 5.73 |
| P0CD71 | Elongation factor Tu OS=Chlamydia trachomatis (strain D/UW-3/Cx) GN=tuf PE=3 SV=1 - [EFTU_CHLTR] | 61.8 | 33.5 | 8 | 14 | 43.3 | 5.53 |
| O84588 | Protein CT_584 OS=Chlamydia trachomatis (strain D/UW-3/Cx) GN=CT_584 PE=3 SV=1 - [Y584_CHLTR] | 56.8 | 33.9 | 4 | 29 | 21.1 | 5.87 |
| B0B8Q5 | Elongation factor Ts OS=Chlamydia trachomatis serovar L2 (strain 434/Bu / ATCC VR-902B) GN=tsf PE=3 SV=1 - [EFTS_CHLT2] | 52.7 | 15.6 | 3 | 14 | 30.9 | 5.90 |
| A0A0E9CNY3 | Glyceraldehyde-3-phosphate dehydrogenase OS=Chlamydia trachomatis GN=gap PE=3 SV=1 - [A0A0E9CNY3_CHLTH] | 51.3 | 32.2 | 4 | 15 | 19.1 | 5.17 |
| B0B9N4 | 30S ribosomal protein S9 OS=Chlamydia trachomatis serovar L2 (strain 434/Bu / ATCC VR-902B) GN=rpsI PE=3 SV=1 - [RS9_CHLT2] | 48.7 | 20.9 | 2 | 14 | 14.5 | 11.02 |
| A0A0E9D199 | ATP-dependent Clp protease proteolytic subunit OS=Chlamydia trachomatis GN=clpP-2 PE=3 SV=1 - [A0A0E9D199_CHLTH] | 46.2 | 19.3 | 2 | 12 | 21.1 | 5.59 |
| A0A0E9AW79 | Type III secretion structural protein OS=Chlamydia trachomatis GN=pilQ PE=3 SV=1 - [A0A0E9AW79_CHLTH] | 42.9 | 9.7 | 5 | 10 | 100.2 | 5.76 |
| A0A0E9CZX7 | Type III secretion system ATPase OS=Chlamydia trachomatis GN=sctN PE=4 SV=1 - [A0A0E9CZX7_CHLTH] | 38.9 | 9.5 | 3 | 10 | 48.2 | 5.81 |
| Q1ALV0 | S2 ribosomal protein (Fragment) OS=Chlamydia trachomatis GN=rs2 PE=3 SV=1 - [Q1ALV0_CHLTH] | 38.3 | 19.7 | 2 | 9 | 15.4 | 8.88 |
| A0A0E9AQW3 | Polymorphic outer membrane protein OS=Chlamydia trachomatis GN=ERS066953_00399 PE=4 SV=1 - [A0A0E9AQW3_CHLTH] | 36.7 | 5.9 | 5 | 9 | 160.4 | 4.88 |
| A0A0E9CNS8 | Polymorphic outer membrane protein OS=Chlamydia trachomatis GN=pmpF_1 PE=4 SV=1 - [A0A0E9CNS8_CHLTH] | 33.5 | 14.9 | 5 | 14 | 83.5 | 5.97 |
| A0A0E9B124 | Transaldolase OS=Chlamydia trachomatis GN=talA PE=3 SV=1 - [A0A0E9B124_CHLTH] | 32.2 | 10.4 | 2 | 8 | 36.1 | 5.03 |
| A0A0E9CKY8 | Serine protease OS=Chlamydia trachomatis GN=htrA PE=4 SV=1 - [A0A0E9CKY8_CHLTH] | 31.7 | 3.9 | 1 | 8 | 34.9 | 5.57 |
| A8KPL3 | Thio-Specific Antioxidant (TSA) Peroxidase (Fragment) OS=Chlamydia trachomatis GN=ahpC PE=4 SV=1 - [A8KPL3_CHLTH] | 31.3 | 32.8 | 4 | 8 | 21.7 | 4.86 |
| A0A0E9FYU5 | 30S ribosomal protein S11 OS=Chlamydia trachomatis GN=rpsK_1 PE=3 SV=1 - [A0A0E9FYU5_CHLTH] | 28.8 | 16.9 | 1 | 7 | 9.2 | 10.95 |
| Q3KLX6 | Glucose-6-phosphate isomerase OS=Chlamydia trachomatis serovar A (strain ATCC VR-571B / DSM 19440 / HAR-13) GN=pgi PE=3 SV=2 - [G6PI_CHLTA] | 25.8 | 20.2 | 6 | 6 | 57.6 | 6.06 |
| A0A0E9ESU7 | Chaperone protein DnaK OS=Chlamydia trachomatis GN=dnaK_3 PE=2 SV=1 - [A0A0E9ESU7_CHLTH] | 25.6 | 2.6 | 1 | 6 | 65.5 | 4.73 |
| A0A0E9D869 | Protein disulfide isomerase OS=Chlamydia trachomatis GN=dsbH_2 PE=4 SV=1 - [A0A0E9D869_CHLTH] | 25.1 | 29.9 | 3 | 6 | 18.5 | 8.32 |
| A0A0E9CZQ4 | Enolase OS=Chlamydia trachomatis GN=eno PE=3 SV=1 - [A0A0E9CZQ4_CHLTH] | 23.9 | 8.0 | 2 | 6 | 45.4 | 4.73 |
| B0B895 | 50S ribosomal protein L16 OS=Chlamydia trachomatis serovar L2 (strain 434/Bu / ATCC VR-902B) GN=rplP PE=3 SV=1 - [RL16_CHLT2] | 22.7 | 12.3 | 1 | 5 | 15.8 | 11.30 |
| A0A0H2X245 | Polymorphic outer membrane protein OS=Chlamydia trachomatis serovar A (strain ATCC VR-571B / DSM 19440 / HAR-13) GN=pmpF PE=4 SV=1 - [A0A0H2X245_CHLTA] | 21.6 | 9.3 | 5 | 5 | 112.7 | 8.34 |
| O84296 | Superoxide dismutase [Mn] OS=Chlamydia trachomatis (strain D/UW-3/Cx) GN=sodA PE=3 SV=1 - [SODM_CHLTR] | 21.3 | 6.3 | 1 | 5 | 23.5 | 6.33 |
| A0A0E9C5C6 | Elongation factor G OS=Chlamydia trachomatis GN=fusA PE=3 SV=1 - [A0A0E9C5C6_CHLTH] | 20.2 | 5.2 | 2 | 5 | 76.5 | 5.34 |
| A0A0E9CQA8 | Fructose-bisphosphate aldolase OS=Chlamydia trachomatis GN=fbaB PE=4 SV=1 - [A0A0E9CQA8_CHLTH] | 19.9 | 22.0 | 1 | 6 | 9.9 | 7.99 |
| A0A0E9AUN0 | Outer membrane protein OS=Chlamydia trachomatis GN=ERS066953_00140 PE=4 SV=1 - [A0A0E9AUN0_CHLTH] | 19.7 | 4.0 | 2 | 5 | 88.7 | 9.03 |
| Q83U76 | Polymorphic membrane protein H (Fragment) OS=Chlamydia trachomatis GN=pmpH PE=4 SV=1 - [Q83U76_CHLTH] | 19.6 | 4.0 | 2 | 5 | 104.9 | 6.43 |
| A0A0E9AZS4 | Peptidyl-prolyl cis-trans isomerase OS=Chlamydia trachomatis GN=mip PE=4 SV=1 - [A0A0E9AZS4_CHLTH] | 19.4 | 19.7 | 3 | 5 | 25.5 | 4.88 |
| Q2TGM2 | Polymorphic membrane protein B OS=Chlamydia trachomatis GN=pmpB PE=4 SV=1 - [Q2TGM2_CHLTH] | 19.0 | 5.1 | 4 | 4 | 183 | 5.92 |
| A0A0H2X1M5 | Phosphopeptide binding protein OS=Chlamydia trachomatis serovar A (strain ATCC VR-571B / DSM 19440 / HAR-13) GN=CTA_0721 PE=4 SV=1 - [A0A0H2X1M5_CHLTA] | 18.8 | 8.1 | 4 | 5 | 89.6 | 4.60 |
| B2XRL7 | Tarp protein OS=Chlamydia trachomatis PE=4 SV=1 - [B2XRL7_CHLTH] | 18.8 | 7.7 | 3 | 5 | 100.5 | 4.46 |
| A0A0E9ATJ7 | Probable cytosol aminopeptidase OS=Chlamydia trachomatis GN=pepA_1 PE=3 SV=1 - [A0A0E9ATJ7_CHLTH] | 18.8 | 10.2 | 4 | 5 | 54.2 | 6.00 |
| B0B926 | 50S ribosomal protein L25 OS=Chlamydia trachomatis serovar L2 (strain 434/Bu / ATCC VR-902B) GN=rplY PE=3 SV=1 - [RL25_CHLT2] | 18.8 | 28.1 | 3 | 5 | 20.4 | 8.76 |
| Q58HD1 | Major outer membrane porin (Fragment) OS=Chlamydia trachomatis GN=omp1 PE=3 SV=1 - [Q58HD1_CHLTH] | 18.5 | 6.1 | 1 | 5 | 33.9 | 4.88 |
| K0GGT0 | Ribosome-recycling factor OS=Chlamydia trachomatis GN=frr PE=3 SV=1 - [K0GGT0_CHLTH] | 18.0 | 17.9 | 2 | 6 | 20 | 8.56 |
| A0A0E9FYS4 | Thioredoxin reductase OS=Chlamydia trachomatis GN=trxB_1 PE=3 SV=1 - [A0A0E9FYS4_CHLTH] | 17.0 | 18.0 | 3 | 4 | 33.5 | 5.95 |
| P0CE08 | DNA-directed RNA polymerase subunit alpha OS=Chlamydia trachomatis (strain D/UW-3/Cx) GN=rpoA PE=3 SV=1 - [RPOA_CHLTR] | 16.0 | 7.7 | 2 | 4 | 41.8 | 5.47 |
| A0A0E9CYI8 | 50S ribosomal protein L10 OS=Chlamydia trachomatis GN=rplJ PE=3 SV=1 - [A0A0E9CYI8_CHLTH] | 15.6 | 14.5 | 2 | 5 | 18.8 | 6.34 |
| Q83U05 | Polymorphic membrane protein E (Fragment) OS=Chlamydia trachomatis GN=pmpE PE=4 SV=1 - [Q83U05_CHLTH] | 15.5 | 5.4 | 3 | 4 | 101.6 | 7.21 |
| A0A0E9E9K2 | Protein translocase subunit OS=Chlamydia trachomatis GN=ERS066953_01398 PE=4 SV=1 - [A0A0E9E9K2_CHLTH] | 15.1 | 12.3 | 1 | 4 | 12.8 | 9.20 |
| A0A0E9CXT7 | N utilization substance protein A OS=Chlamydia trachomatis GN=nusA_2 PE=4 SV=1 - [A0A0E9CXT7_CHLTH] | 14.9 | 11.9 | 2 | 4 | 32.9 | 5.02 |
| A0A0E9CZR5 | Histone H1--like developmental protein OS=Chlamydia trachomatis GN=hctA PE=4 SV=1 - [A0A0E9CZR5_CHLTH] | 13.2 | 11.2 | 1 | 3 | 13.7 | 10.70 |
| A0A0E9E330 | Anti-sigma factor antagonist OS=Chlamydia trachomatis GN=btrV PE=3 SV=1 - [A0A0E9E330_CHLTH] | 13.0 | 41.4 | 3 | 3 | 12.5 | 5.39 |
| B0B9B1 | Aspartyl/glutamyl-tRNA(Asn/Gln) amidotransferase subunit B OS=Chlamydia trachomatis serovar L2 (strain 434/Bu / ATCC VR-902B) GN=gatB PE=3 SV=1 - [GATB_CHLT2] | 12.8 | 6.8 | 2 | 3 | 54.9 | 6.32 |
| B0B8V8 | 6,7-dimethyl-8-ribityllumazine synthase OS=Chlamydia trachomatis serovar L2 (strain 434/Bu / ATCC VR-902B) GN=ribH PE=3 SV=1 - [RISB_CHLT2] | 12.4 | 33.8 | 3 | 3 | 16.4 | 6.74 |
| A0A0E9D2X3 | 30S ribosomal protein S3 OS=Chlamydia trachomatis GN=rpsC PE=3 SV=1 - [A0A0E9D2X3_CHLTH] | 12.4 | 15.5 | 2 | 3 | 23.9 | 10.02 |
| A0A0E9CSV1 | MotA/TolQ/ExbB proton channel family protein OS=Chlamydia trachomatis GN=tolQ PE=3 SV=1 - [A0A0E9CSV1_CHLTH] | 12.4 | 16.4 | 1 | 3 | 14.5 | 6.52 |
| A0A0E9CB48 | Transcription termination factor Rho OS=Chlamydia trachomatis GN=rho PE=3 SV=1 - [A0A0E9CB48_CHLTH] | 12.2 | 6.9 | 2 | 3 | 51.7 | 7.36 |
| A0A0E9CGP2 | 30S ribosomal protein S13 OS=Chlamydia trachomatis GN=rpsM PE=3 SV=1 - [A0A0E9CGP2_CHLTH] | 11.9 | 25.4 | 2 | 3 | 13.8 | 10.71 |
| A0A0E9CN91 | Amino acid ABC transporter substrate-binding protein OS=Chlamydia trachomatis GN=ERS075194_01180 PE=4 SV=1 - [A0A0E9CN91_CHLTH] | 11.7 | 24.3 | 1 | 3 | 7.6 | 10.26 |
| P0CE01 | 50S ribosomal protein L6 OS=Chlamydia trachomatis (strain D/UW-3/Cx) GN=rplF PE=3 SV=1 - [RL6_CHLTR] | 11.4 | 18.0 | 2 | 3 | 19.8 | 9.96 |
| A0A0E9D403 | Peptide deformylase OS=Chlamydia trachomatis GN=def PE=3 SV=1 - [A0A0E9D403_CHLTH] | 9.4 | 24.3 | 2 | 2 | 20.5 | 5.90 |
| A0A0E9DQ65 | Leucine dehydrogenase OS=Chlamydia trachomatis GN=ldh PE=4 SV=1 - [A0A0E9DQ65_CHLTH] | 9.3 | 23.3 | 1 | 2 | 11.1 | 8.13 |
| A0A0E9DNL0 | Polymorphic outer membrane protein OS=Chlamydia trachomatis GN=pmpI PE=4 SV=1 - [A0A0E9DNL0_CHLTH] | 8.5 | 10.0 | 1 | 2 | 21.2 | 9.19 |
| B7SC61 | PorB (Fragment) OS=Chlamydia trachomatis GN=porB PE=4 SV=1 - [B7SC61_CHLTH] | 8.4 | 18.5 | 1 | 2 | 13.8 | 4.78 |
| O84666 | UPF0109 protein CT_659 OS=Chlamydia trachomatis (strain D/UW-3/Cx) GN=CT_659 PE=3 SV=1 - [Y659_CHLTR] | 8.4 | 35.9 | 1 | 2 | 8.8 | 8.43 |
| A0A0E9CPX5 | 30S ribosomal protein S1 OS=Chlamydia trachomatis GN=rpsA PE=4 SV=1 - [A0A0E9CPX5_CHLTH] | 8.2 | 10.7 | 2 | 2 | 17.3 | 5.15 |
| A0A0E9AZ23 | Succinyl-CoA ligase [ADP-forming] subunit alpha OS=Chlamydia trachomatis GN=sucD PE=3 SV=1 - [A0A0E9AZ23_CHLTH] | 8.1 | 6.2 | 1 | 2 | 30.2 | 5.48 |
| A0A0E9C6A0 | Pyruvate kinase OS=Chlamydia trachomatis GN=pykF PE=3 SV=1 - [A0A0E9C6A0_CHLTH] | 8.1 | 3.3 | 1 | 2 | 53.7 | 6.40 |
| A0A0E9CCK9 | Transcription termination/antitermination protein NusG OS=Chlamydia trachomatis GN=nusG PE=3 SV=1 - [A0A0E9CCK9_CHLTH] | 8.0 | 18.7 | 2 | 2 | 20.7 | 5.31 |
| A0A0E9CM38 | Polynucleotide phosphorylase/polyadenylase OS=Chlamydia trachomatis GN=pnp PE=4 SV=1 - [A0A0E9CM38_CHLTH] | 7.8 | 11.3 | 2 | 2 | 27.2 | 6.42 |
| A0A0E9EAX5 | Putative protease OS=Chlamydia trachomatis GN=ERS133246_04554 PE=4 SV=1 - [A0A0E9EAX5_CHLTH] | 7.8 | 4.6 | 1 | 2 | 42.3 | 6.43 |
| A0A0E9ATU0 | Oligoendopeptidase F OS=Chlamydia trachomatis GN=pepF PE=4 SV=1 - [A0A0E9ATU0_CHLTH] | 7.8 | 5.6 | 2 | 2 | 69 | 5.95 |
| A0A0E9AR22 | Uncharacterized protein OS=Chlamydia trachomatis GN=greA_1 PE=3 SV=1 - [A0A0E9AR22_CHLTH] | 7.7 | 4.8 | 2 | 2 | 80.9 | 5.38 |
| A0A0E9B081 | ATP-dependent Clp protease%2C subunit B OS=Chlamydia trachomatis GN=clpB PE=3 SV=1 - [A0A0E9B081_CHLTH] | 7.7 | 1.5 | 1 | 2 | 96.6 | 5.47 |
| B0BCD9 | Nucleoside diphosphate kinase OS=Chlamydia trachomatis serovar L2b (strain UCH-1/proctitis) GN=ndk PE=3 SV=1 - [NDK_CHLTB] | 7.6 | 8.5 | 1 | 2 | 15.3 | 5.49 |
| A0A0E9CV36 | Serine hydroxymethyltransferase OS=Chlamydia trachomatis GN=glyA_2 PE=4 SV=1 - [A0A0E9CV36_CHLTH] | 7.6 | 15.9 | 2 | 2 | 19.5 | 6.81 |
| A0A0E9AVM4 | 6-phosphogluconate dehydrogenase, decarboxylating OS=Chlamydia trachomatis GN=gnd PE=3 SV=1 - [A0A0E9AVM4_CHLTH] | 7.5 | 6.9 | 2 | 2 | 52.6 | 5.63 |
| A0A0E9CD67 | 50S ribosomal protein L2 OS=Chlamydia trachomatis GN=rplB PE=3 SV=1 - [A0A0E9CD67_CHLTH] | 7.5 | 5.3 | 2 | 2 | 31.4 | 10.43 |
| P64386 | Probable DNA-binding protein HU OS=Chlamydia trachomatis (strain D/UW-3/Cx) GN=hup PE=3 SV=1 - [DBH_CHLTR] | 7.5 | 10.0 | 1 | 2 | 11.4 | 11.06 |
| A0A0E9CTT3 | Polymorphic outer membrane protein OS=Chlamydia trachomatis GN=pmpC PE=4 SV=1 - [A0A0E9CTT3_CHLTH] | 7.3 | 3.2 | 1 | 2 | 55.1 | 6.62 |
| A0A0E9CX90 | 5'-nucleotidase SurE OS=Chlamydia trachomatis GN=surE PE=3 SV=1 - [A0A0E9CX90_CHLTH] | 7.3 | 10.3 | 2 | 2 | 31.5 | 4.92 |
| A0A0E9CAZ5 | 30S ribosomal protein S4 OS=Chlamydia trachomatis GN=rpsD PE=3 SV=1 - [A0A0E9CAZ5_CHLTH] | 4.9 | 11.5 | 1 | 1 | 23.8 | 10.05 |
| A0A0E9CN44 | Malonyl-CoA-[acyl-carrier-protein] transacylase OS=Chlamydia trachomatis GN=fabD PE=4 SV=1 - [A0A0E9CN44_CHLTH] | 4.7 | 13.6 | 1 | 1 | 17.9 | 5.27 |
| A0A0E9D5V8 | 30S ribosomal protein S16 OS=Chlamydia trachomatis GN=rpsP PE=3 SV=1 - [A0A0E9D5V8_CHLTH] | 4.7 | 12.1 | 1 | 1 | 13.4 | 10.46 |
| Q3KLT4 | 50S ribosomal protein L27 OS=Chlamydia trachomatis serovar A (strain ATCC VR-571B / DSM 19440 / HAR-13) GN=rpmA PE=3 SV=1 - [RL27_CHLTA] | 4.6 | 21.7 | 1 | 1 | 8.9 | 11.03 |
| A0A0E9CE81 | Phosphoglycerate kinase OS=Chlamydia trachomatis GN=pgk_1 PE=3 SV=1 - [A0A0E9CE81_CHLTH] | 4.6 | 6.5 | 1 | 1 | 43 | 5.97 |
| A0A0E9AZU9 | Type III secretion cytoplasmic membrane protein SctJ OS=Chlamydia trachomatis GN=prgK PE=4 SV=1 - [A0A0E9AZU9_CHLTH] | 4.4 | 4.9 | 1 | 1 | 35.5 | 5.72 |
| A0A0E9CRL6 | Thymidylate kinase OS=Chlamydia trachomatis GN=tmk PE=4 SV=1 - [A0A0E9CRL6_CHLTH] | 4.3 | 23.8 | 1 | 1 | 9.6 | 8.51 |
| A0A0E9F314 | LSU ribosomal protein L1E (L4P) OS=Chlamydia trachomatis GN=ERS133246_06945 PE=4 SV=1 - [A0A0E9F314_CHLTH] | 4.3 | 15.3 | 1 | 1 | 12.1 | 7.14 |
| B0B9B0 | Glutamyl-tRNA(Gln) amidotransferase subunit A OS=Chlamydia trachomatis serovar L2 (strain 434/Bu / ATCC VR-902B) GN=gatA PE=3 SV=1 - [GATA_CHLT2] | 4.3 | 3.1 | 1 | 1 | 53.6 | 6.23 |
| A0A0E9CRP3 | HAD superfamily hydrolase/phosphatase OS=Chlamydia trachomatis GN=ERS075194_01797 PE=4 SV=1 - [A0A0E9CRP3_CHLTH] | 4.3 | 8.6 | 1 | 1 | 17.3 | 4.82 |
| A0A0E9DD28 | Conjugal transfer ATP-binding protein TraC OS=Chlamydia trachomatis GN=ERS082928_01128 PE=4 SV=1 - [A0A0E9DD28_CHLTH] | 4.2 | 27.2 | 1 | 2 | 16.7 | 8.28 |
| A0A0E9DVP9 | Uncharacterised protein OS=Chlamydia trachomatis GN=ERS133246_01556 PE=4 SV=1 - [A0A0E9DVP9_CHLTH] | 4.2 | 11.8 | 1 | 1 | 20 | 9.26 |
| A0A0E9B1X8 | Thiol:disulfide interchange protein OS=Chlamydia trachomatis GN=ERS066955_00827 PE=4 SV=1 - [A0A0E9B1X8_CHLTH] | 4.2 | 4.7 | 1 | 1 | 49.2 | 5.38 |
| A0A0E9G272 | Thioredoxin OS=Chlamydia trachomatis GN=trxA PE=4 SV=1 - [A0A0E9G272_CHLTH] | 4.1 | 28.6 | 1 | 1 | 7.7 | 6.54 |
| A0A0E9B0E1 | Enoyl-ACP reductase OS=Chlamydia trachomatis GN=fabI PE=4 SV=1 - [A0A0E9B0E1_CHLTH] | 4.1 | 5.0 | 1 | 1 | 32 | 5.35 |
| B0B7M2 | V-type ATP synthase subunit D OS=Chlamydia trachomatis serovar L2 (strain 434/Bu / ATCC VR-902B) GN=atpD PE=3 SV=1 - [VATD_CHLT2] | 4.1 | 5.9 | 1 | 1 | 23.2 | 8.98 |
| A0A0E9AZE6 | Uncharacterised protein OS=Chlamydia trachomatis GN=ERS066955_00730 PE=4 SV=1 - [A0A0E9AZE6_CHLTH] | 4.1 | 5.0 | 1 | 1 | 36.2 | 4.75 |
| A0A0E9CZ22 | Acetyl-CoA carboxylase biotin carboxyl carrier protein subunit OS=Chlamydia trachomatis GN=accB PE=4 SV=1 - [A0A0E9CZ22_CHLTH] | 4.0 | 10.4 | 1 | 1 | 18.2 | 5.12 |
| A0A0E9CQM4 | Protein RecA OS=Chlamydia trachomatis GN=recA PE=3 SV=1 - [A0A0E9CQM4_CHLTH] | 3.9 | 5.3 | 1 | 1 | 36.7 | 6.80 |
| A0A0E9ATY1 | Putative cytosolic protein OS=Chlamydia trachomatis GN=ERS066953_00424 PE=4 SV=1 - [A0A0E9ATY1_CHLTH] | 3.9 | 11.0 | 1 | 1 | 18.4 | 4.61 |
| W8VNJ2 | Major outer membrane porin (Fragment) OS=Chlamydia trachomatis GN=ompA PE=3 SV=1 - [W8VNJ2_CHLTH] | 3.9 | 5.6 | 1 | 1 | 36.8 | 4.98 |
| Q6LCD5 | 10 kDa chaperonin (Fragment) OS=Chlamydia trachomatis GN=hypA PE=3 SV=1 - [Q6LCD5_CHLTH] | 3.9 | 18.6 | 1 | 1 | 10.7 | 4.94 |
| A0A0E9F3V8 | Putative cytosolic protein OS=Chlamydia trachomatis GN=ERS095036_10045 PE=4 SV=1 - [A0A0E9F3V8_CHLTH] | 3.9 | 8.0 | 1 | 1 | 22.2 | 4.72 |
| A0A0E9CRR3 | Putative nucleotide transport protein OS=Chlamydia trachomatis GN=tlcA_1 PE=4 SV=1 - [A0A0E9CRR3_CHLTH] | 3.9 | 5.5 | 1 | 1 | 24.4 | 9.95 |
| A0A0E9CUI5 | Uridylate kinase OS=Chlamydia trachomatis GN=pyrH_3 PE=4 SV=1 - [A0A0E9CUI5_CHLTH] | 3.8 | 24.0 | 1 | 1 | 10.5 | 4.96 |
| B0B889 | 30S ribosomal protein S8 OS=Chlamydia trachomatis serovar L2 (strain 434/Bu / ATCC VR-902B) GN=rpsH PE=3 SV=1 - [RS8_CHLT2] | 3.8 | 19.6 | 1 | 1 | 15.1 | 10.27 |
| A0A0E9EK46 | Outer membrane protein OS=Chlamydia trachomatis GN=ompH PE=4 SV=1 - [A0A0E9EK46_CHLTH] | 3.8 | 15.5 | 1 | 1 | 14 | 4.60 |
| Q3KM43 | 50S ribosomal protein L11 OS=Chlamydia trachomatis serovar A (strain ATCC VR-571B / DSM 19440 / HAR-13) GN=rplK PE=3 SV=1 - [RL11_CHLTA] | 3.7 | 7.1 | 1 | 1 | 15.1 | 9.67 |
| A0A0E9CV83 | Carbohydrate-selective porin%2C OprB family protein OS=Chlamydia trachomatis GN=aaxA PE=4 SV=1 - [A0A0E9CV83_CHLTH] | 3.7 | 5.2 | 1 | 1 | 30.1 | 9.11 |
| A0A0E9AVY3 | Translation initiation factor IF-3 OS=Chlamydia trachomatis GN=infC_1 PE=4 SV=1 - [A0A0E9AVY3_CHLTH] | 3.7 | 20.0 | 1 | 1 | 8.4 | 6.67 |
| B0B928 | 30S ribosomal protein S6 OS=Chlamydia trachomatis serovar L2 (strain 434/Bu / ATCC VR-902B) GN=rpsF PE=3 SV=1 - [RS6_CHLT2] | 3.7 | 12.5 | 1 | 1 | 12.9 | 8.81 |
| A0A0E9DP73 | Protein of uncharacterised function (DUF1207) OS=Chlamydia trachomatis GN=ERS095037_01102 PE=4 SV=1 - [A0A0E9DP73_CHLTH] | 3.7 | 4.1 | 1 | 1 | 39.1 | 6.07 |
| A0A0E9EUP2 | ATP synthase subunit alpha OS=Chlamydia trachomatis GN=sctN_2 PE=3 SV=1 - [A0A0E9EUP2_CHLTH] | 3.7 | 2.3 | 1 | 1 | 56.1 | 5.05 |
| A0A0E9CRA0 | Malate dehydrogenase OS=Chlamydia trachomatis GN=mdh PE=4 SV=1 - [A0A0E9CRA0_CHLTH] | 3.6 | 11.2 | 1 | 1 | 11.6 | 5.45 |
| A0A0E9C8R9 | 50S ribosomal protein L18 OS=Chlamydia trachomatis GN=rplR PE=3 SV=1 - [A0A0E9C8R9_CHLTH] | 3.6 | 10.6 | 1 | 1 | 13.4 | 10.32 |
| G4NNN6 | Uncharacterized protein OS=Chlamydia trachomatis serovar A (strain A2497) GN=CTO_0755 PE=4 SV=1 - [G4NNN6_CHLT4] | 3.6 | 7.1 | 1 | 1 | 34.6 | 5.17 |
| A0A0E9AZP9 | Cell division protein OS=Chlamydia trachomatis GN=ERS066955_00453 PE=4 SV=1 - [A0A0E9AZP9_CHLTH] | 3.6 | 3.3 | 1 | 1 | 45.2 | 8.05 |
| A0A0E9D2D6 | Cation efflux system protein OS=Chlamydia trachomatis GN=ERS075185_00347 PE=4 SV=1 - [A0A0E9D2D6_CHLTH] | 3.6 | 3.6 | 1 | 1 | 60.9 | 6.11 |
| A0A0E9ERX5 | Uncharacterised protein OS=Chlamydia trachomatis GN=ERS095036_06689 PE=4 SV=1 - [A0A0E9ERX5_CHLTH] | 3.6 | 3.2 | 1 | 1 | 43.5 | 9.09 |
| B0B9D5 | 50S ribosomal protein L19 OS=Chlamydia trachomatis serovar L2 (strain 434/Bu / ATCC VR-902B) GN=rplS PE=3 SV=1 - [RL19_CHLT2] | 3.6 | 9.1 | 1 | 1 | 13.1 | 9.94 |
| A0A0E9B1C0 | Protease IV OS=Chlamydia trachomatis GN=sohB PE=4 SV=1 - [A0A0E9B1C0_CHLTH] | 3.6 | 4.5 | 1 | 1 | 35.7 | 7.97 |
| A0A0E9CRD3 | Deoxyuridine 5'-triphosphate nucleotidohydrolase OS=Chlamydia trachomatis GN=dut PE=4 SV=1 - [A0A0E9CRD3_CHLTH] | 3.5 | 16.5 | 1 | 1 | 11.6 | 5.39 |

**Table E4**. PEAKS DB identification of 190 CtB protein hits with FDR of 0.1% (on peptide level) and 0% (on protein level). Abbreviations: Cov. = protein sequence coverage, # Uniq. Pep. = number of unique peptides # Pep. = number of peptides

| **Accession** | **Description** | **Score**  **(-10lgP)** | **Cov.**  **[%]** | **# Pep.** | **# Uniq. Pep.** | **MW**  **[kDa]** |
| --- | --- | --- | --- | --- | --- | --- |
| G4NM84\|G4NM84_CHLT4 | 60 kDa chaperonin OS=Chlamydia trachomatis serovar A (strain A2497) GN=groL PE=3 SV=1 | 361.4 | 70 | 46 | 3 | 58.1 |
| sp\|Q3KMQ9\|CH60_CHLTA | 60 kDa chaperonin OS=Chlamydia trachomatis serovar A (strain ATCC VR-571B / DSM 19440 / HAR-13) GN=groL PE=2 SV=3 | 361.4 | 70 | 46 | 3 | 58.1 |
| A0A0E9CT34\|A0A0E9CT34_CHLTH | Elongation factor Tu OS=Chlamydia trachomatis GN=tuf PE=3 SV=1 | 260.0 | 71 | 30 | 2 | 43.3 |
| sp\|Q3KM40\|EFTU_CHLTA | Elongation factor Tu OS=Chlamydia trachomatis serovar A (strain ATCC VR-571B / DSM 19440 / HAR-13) GN=tuf PE=3 SV=1 | 260.0 | 71 | 30 | 2 | 43.3 |
| Q2TV41\|Q2TV41_CHLTH | Polymorphic membrane protein D OS=Chlamydia trachomatis GN=pmpD PE=4 SV=1 | 254.0 | 14 | 20 | 2 | 160.7 |
| A0A0E9DIU3\|A0A0E9DIU3_CHLTH | Gag gene protein p24 (Core nucleocapsid protein) OS=Chlamydia trachomatis GN=ERS082929_01031 PE=4 SV=1 | 260.6 | 29 | 15 | 3 | 65.7 |
| sp\|Q3KMX6\|AMPA_CHLTA | Probable cytosol aminopeptidase OS=Chlamydia trachomatis serovar A (strain ATCC VR-571B / DSM 19440 / HAR-13) GN=pepA PE=3 SV=1 | 263.4 | 34 | 13 | 2 | 54.1 |
| G4NLX2\|G4NLX2_CHLT4 | DegP OS=Chlamydia trachomatis serovar A (strain A2497) GN=CTO_0897 PE=4 SV=1 | 169.7 | 12 | 6 | 2 | 53.3 |
| Q6LCD5\|Q6LCD5_CHLTH | 10 kDa chaperonin (Fragment) OS=Chlamydia trachomatis GN=hypA PE=3 SV=1 | 148.0 | 38 | 3 | 3 | 10.7 |
| A0A0E9CAW6\|A0A0E9CAW6_CHLTH | 10 kDa chaperonin OS=Chlamydia trachomatis GN=groES PE=3 SV=1 | 148.0 | 36 | 3 | 3 | 11.2 |
| A0A0H2X1N0\|A0A0H2X1N0_CHLTA | SctN OS=Chlamydia trachomatis serovar A (strain ATCC VR-571B / DSM 19440 / HAR-13) GN=sctN PE=4 SV=1 | 242.0 | 38 | 14 | 2 | 48.2 |
| G4NN56\|G4NN56_CHLT4 | SctN OS=Chlamydia trachomatis serovar A (strain A2497) GN=CTO_0726 PE=4 SV=1 | 242.0 | 38 | 14 | 2 | 48.2 |
| G4NMP7\|G4NMP7_CHLT4 | Thioredoxin peroxidase OS=Chlamydia trachomatis serovar A (strain A2497) GN=CTO_0654 PE=4 SV=1 | 268.5 | 67 | 13 | 13 | 22.7 |
| A0A0H2X1C0\|A0A0H2X1C0_CHLTA | Thioredoxin peroxidase OS=Chlamydia trachomatis serovar A (strain ATCC VR-571B / DSM 19440 / HAR-13) GN=ahpC PE=4 SV=1 | 268.5 | 67 | 13 | 13 | 22.7 |
| sp\|O84217\|ALF1_CHLTR | Probable fructose-bisphosphate aldolase class 1 OS=Chlamydia trachomatis (strain D/UW-3/Cx) GN=fbaB PE=3 SV=1 | 185.3 | 37 | 10 | 3 | 38.0 |
| A0A0E9F3I9\|A0A0E9F3I9_CHLTH | ATP synthase subunit beta OS=Chlamydia trachomatis GN=sctN_4 PE=3 SV=1 | 153.7 | 8 | 3 | 2 | 50.7 |
| sp\|O84031\|RL19_CHLTR | 50S ribosomal protein L19 OS=Chlamydia trachomatis (strain D/UW-3/Cx) GN=rplS PE=3 SV=1 | 164.9 | 49 | 7 | 7 | 13.1 |
| sp\|B0BB14\|RL19_CHLTB | 50S ribosomal protein L19 OS=Chlamydia trachomatis serovar L2b (strain UCH-1/proctitis) GN=rplS PE=3 SV=1 | 164.9 | 49 | 7 | 7 | 13.1 |
| sp\|O84805\|RL25_CHLTR | 50S ribosomal protein L25 OS=Chlamydia trachomatis (strain D/UW-3/Cx) GN=rplY PE=3 SV=1 | 189.7 | 46 | 6 | 6 | 20.4 |
| G4NP91\|G4NP91_CHLT4 | Integration host factor beta-subunit OS=Chlamydia trachomatis serovar A (strain A2497) GN=CTO_0289 PE=3 SV=1 | 124.5 | 21 | 3 | 3 | 17.0 |
| M9UF21\|M9UF21_CHLTH | Integration host factor OS=Chlamydia trachomatis L2/434/Bu(f) GN=CTLFINAL_04880 PE=3 SV=2 | 124.5 | 21 | 3 | 3 | 17.0 |
| A0A0E9AUE0\|A0A0E9AUE0_CHLTH | Uncharacterised protein OS=Chlamydia trachomatis GN=ERS066953_01368 PE=4 SV=1 | 213.1 | 50 | 8 | 8 | 21.1 |
| sp\|O84588\|Y584_CHLTR | Protein CT_584 OS=Chlamydia trachomatis (strain D/UW-3/Cx) GN=CT_584 PE=3 SV=1 | 213.1 | 50 | 8 | 8 | 21.1 |
| A0A0H2X1F6\|A0A0H2X1F6_CHLTA | Uncharacterized protein OS=Chlamydia trachomatis serovar A (strain ATCC VR-571B / DSM 19440 / HAR-13) GN=CTA_0634 PE=4 SV=1 | 213.1 | 50 | 8 | 8 | 21.1 |
| M9UIQ4\|M9UIQ4_CHLTH | Uncharacterized protein OS=Chlamydia trachomatis L2/434/Bu(f) GN=CTLFINAL_04420 PE=4 SV=1 | 213.1 | 50 | 8 | 8 | 21.1 |
| G4NMM5\|G4NMM5_CHLT4 | Uncharacterized protein OS=Chlamydia trachomatis serovar A (strain A2497) GN=CTO_0634 PE=4 SV=1 | 213.1 | 50 | 8 | 8 | 21.1 |
| A0A0H3MCR9\|A0A0H3MCR9_CHLT2 | Uncharacterized protein OS=Chlamydia trachomatis serovar L2 (strain 434/Bu / ATCC VR-902B) GN=CTL0847 PE=4 SV=1 | 213.1 | 50 | 8 | 8 | 21.1 |
| A0A0E9CFY0\|A0A0E9CFY0_CHLTH | 30S ribosomal protein S1 OS=Chlamydia trachomatis GN=rpsA PE=4 SV=1 | 177.9 | 16 | 10 | 10 | 63.6 |
| sp\|O84100\|RS1_CHLTR | 30S ribosomal protein S1 OS=Chlamydia trachomatis (strain D/UW-3/Cx) GN=rpsA PE=3 SV=1 | 177.9 | 16 | 10 | 10 | 63.6 |
| M9UEF8\|M9UEF8_CHLTH | Low calcium response locus protein D OS=Chlamydia trachomatis L2/434/Bu(f) GN=CTLFINAL_01820 PE=4 SV=1 | 143.0 | 14 | 7 | 7 | 78.0 |
| G4NM61\|G4NM61_CHLT4 | Type III secretion inner membrane protein SctV OS=Chlamydia trachomatis serovar A (strain A2497) GN=CTO_0095 PE=4 SV=1 | 143.0 | 14 | 7 | 7 | 78.0 |
| O84092\|O84092_CHLTR | Low Calcium Response D OS=Chlamydia trachomatis (strain D/UW-3/Cx) GN=lcrD PE=4 SV=1 | 143.0 | 14 | 7 | 7 | 78.0 |
| A0A0H3MCY4\|A0A0H3MCY4_CHLT2 | Low calcium response protein D (Predicted to be part of the TTSS apparatus) OS=Chlamydia trachomatis serovar L2 (strain 434/Bu / ATCC VR-902B) GN=lcrD PE=4 SV=1 | 143.0 | 14 | 7 | 7 | 78.0 |
| A0A0H2X0L8\|A0A0H2X0L8_CHLTA | Low calcium response protein D OS=Chlamydia trachomatis serovar A (strain ATCC VR-571B / DSM 19440 / HAR-13) GN=lcrD PE=4 SV=1 | 143.0 | 14 | 7 | 7 | 78.0 |
| A0A0E9DLE6\|A0A0E9DLE6_CHLTH | Putative membrane transport protein OS=Chlamydia trachomatis GN=invA PE=4 SV=1 | 143.0 | 14 | 7 | 7 | 78.0 |
| A0A0E9CZH0\|A0A0E9CZH0_CHLTH | Putative membrane transport protein OS=Chlamydia trachomatis GN=invA PE=4 SV=1 | 143.0 | 14 | 7 | 7 | 78.0 |
| G4NNS7\|G4NNS7_CHLT4 | 6 7-dimethyl-8-ribityllumazine synthase OS=Chlamydia trachomatis serovar A (strain A2497) GN=ribH PE=3 SV=1 | 191.7 | 41 | 6 | 6 | 17.2 |
| Q83TT3\|Q83TT3_CHLTH | Major outer membrane protein OS=Chlamydia trachomatis GN=porB PE=4 SV=1 | 200.2 | 25 | 7 | 2 | 37.3 |
| A0A0E9CCK9\|A0A0E9CCK9_CHLTH | Transcription termination/antitermination protein NusG OS=Chlamydia trachomatis GN=nusG PE=3 SV=1 | 205.7 | 37 | 5 | 2 | 20.8 |
| K0GGT0\|K0GGT0_CHLTH | Ribosome-recycling factor OS=Chlamydia trachomatis GN=frr PE=3 SV=1 | 208.6 | 45 | 8 | 4 | 20.1 |
| G4NN64\|G4NN64_CHLT4 | Ribosome-recycling factor OS=Chlamydia trachomatis serovar A (strain A2497) GN=frr PE=3 SV=1 | 208.6 | 45 | 8 | 4 | 20.1 |
| A0A0H3MCF1\|A0A0H3MCF1_CHLT2 | Uncharacterized protein OS=Chlamydia trachomatis serovar L2 (strain 434/Bu / ATCC VR-902B) GN=CTL0028 PE=4 SV=1 | 204.7 | 77 | 7 | 7 | 8.8 |
| A0A0E9D2L0\|A0A0E9D2L0_CHLTH | UPF0109 protein ERS066953_00249 OS=Chlamydia trachomatis GN=ERS066953_00249 PE=3 SV=1 | 204.7 | 77 | 7 | 7 | 8.8 |
| M9UG13\|M9UG13_CHLTH | UPF0109 protein CTLFINAL_00155 OS=Chlamydia trachomatis L2/434/Bu(f) GN=CTLFINAL_00155 PE=3 SV=1 | 204.7 | 77 | 7 | 7 | 8.8 |
| A0A0H2X2Z8\|A0A0H2X2Z8_CHLTA | RNA binding protein OS=Chlamydia trachomatis serovar A (strain ATCC VR-571B / DSM 19440 / HAR-13) GN=CTA_0716 PE=4 SV=1 | 204.7 | 77 | 7 | 7 | 8.8 |
| sp\|O84666\|Y659_CHLTR | UPF0109 protein CT_659 OS=Chlamydia trachomatis (strain D/UW-3/Cx) GN=CT_659 PE=3 SV=1 | 204.7 | 77 | 7 | 7 | 8.8 |
| sp\|B0B899\|RL2_CHLT2 | 50S ribosomal protein L2 OS=Chlamydia trachomatis serovar L2 (strain 434/Bu / ATCC VR-902B) GN=rplB PE=3 SV=1 | 193.3 | 25 | 7 | 7 | 31.5 |
| sp\|O84530\|RL2_CHLTR | 50S ribosomal protein L2 OS=Chlamydia trachomatis (strain D/UW-3/Cx) GN=rplB PE=3 SV=1 | 193.3 | 25 | 7 | 7 | 31.5 |
| A0A0E9AXH1\|A0A0E9AXH1_CHLTH | 50S ribosomal protein L2 OS=Chlamydia trachomatis GN=rplB PE=3 SV=1 | 193.3 | 25 | 7 | 7 | 31.5 |
| A0A0H3MLD3\|A0A0H3MLD3_CHLT2 | SSU ribosomal protein S5P OS=Chlamydia trachomatis serovar L2 (strain 434/Bu / ATCC VR-902B) GN=rpsE PE=4 SV=1 | 151.5 | 25 | 5 | 5 | 17.8 |
| sp\|Q3KLI5\|RS5_CHLTA | 30S ribosomal protein S5 OS=Chlamydia trachomatis serovar A (strain ATCC VR-571B / DSM 19440 / HAR-13) GN=rpsE PE=3 SV=1 | 151.5 | 25 | 5 | 5 | 17.8 |
| A0A0E9D3E4\|A0A0E9D3E4_CHLTH | 30S ribosomal protein S5 OS=Chlamydia trachomatis GN=rpsE PE=3 SV=1 | 151.5 | 25 | 5 | 5 | 17.8 |
| M9UIB3\|M9UIB3_CHLTH | 30S ribosomal protein S5 OS=Chlamydia trachomatis L2/434/Bu(f) GN=rpsE PE=3 SV=1 | 151.5 | 25 | 5 | 5 | 17.8 |
| A0A0E9C6W0\|A0A0E9C6W0_CHLTH | Trigger factor OS=Chlamydia trachomatis GN=tig PE=3 SV=1 | 127.8 | 10 | 3 | 3 | 50.1 |
| sp\|Q3KKY7\|TIG_CHLTA | Trigger factor OS=Chlamydia trachomatis serovar A (strain ATCC VR-571B / DSM 19440 / HAR-13) GN=tig PE=3 SV=1 | 127.8 | 10 | 3 | 3 | 50.1 |
| sp\|B0B930\|RL9_CHLT2 | 50S ribosomal protein L9 OS=Chlamydia trachomatis serovar L2 (strain 434/Bu / ATCC VR-902B) GN=rplI PE=3 SV=1 | 125.2 | 23 | 3 | 3 | 18.4 |
| M9ULS5\|M9ULS5_CHLTH | 50S ribosomal protein L9 OS=Chlamydia trachomatis L2/434/Bu(f) GN=rplI PE=3 SV=1 | 125.2 | 23 | 3 | 3 | 18.4 |
| G4NPM5\|G4NPM5_CHLT4 | 50S ribosomal protein L9 OS=Chlamydia trachomatis serovar A (strain A2497) GN=rplI PE=3 SV=1 | 125.2 | 23 | 3 | 3 | 18.4 |
| A0A0E9CXI8\|A0A0E9CXI8_CHLTH | Malate dehydrogenase OS=Chlamydia trachomatis GN=mdh PE=3 SV=1 | 162.9 | 21 | 7 | 2 | 35.7 |
| sp\|O84381\|MDH_CHLTR | Malate dehydrogenase OS=Chlamydia trachomatis (strain D/UW-3/Cx) GN=mdh PE=3 SV=1 | 162.9 | 21 | 7 | 2 | 35.7 |
| sp\|Q3KKP0\|RS6_CHLTA | 30S ribosomal protein S6 OS=Chlamydia trachomatis serovar A (strain ATCC VR-571B / DSM 19440 / HAR-13) GN=rpsF PE=3 SV=1 | 139.2 | 29 | 4 | 4 | 12.9 |
| sp\|B0B928\|RS6_CHLT2 | 30S ribosomal protein S6 OS=Chlamydia trachomatis serovar L2 (strain 434/Bu / ATCC VR-902B) GN=rpsF PE=3 SV=1 | 139.2 | 29 | 4 | 4 | 12.9 |
| M9UGM4\|M9UGM4_CHLTH | 30S ribosomal protein S6 OS=Chlamydia trachomatis L2/434/Bu(f) GN=rpsF PE=3 SV=1 | 139.2 | 29 | 4 | 4 | 12.9 |
| sp\|O84377\|AAXA_CHLTR | Porin AaxA OS=Chlamydia trachomatis (strain D/UW-3/Cx) GN=aaxA PE=3 SV=2 | 125.5 | 10 | 3 | 3 | 51.5 |
| A0A0E9CU42\|A0A0E9CU42_CHLTH | Carbohydrate-selective porin%2C OprB family protein OS=Chlamydia trachomatis GN=aaxA PE=4 SV=1 | 125.5 | 10 | 3 | 3 | 51.5 |
| G4NMV6\|G4NMV6_CHLT4 | Uncharacterized protein OS=Chlamydia trachomatis serovar A (strain A2497) GN=CTO_0404 PE=4 SV=1 | 125.5 | 10 | 3 | 3 | 52.0 |
| sp\|B0BC75\|RS7_CHLTB | 30S ribosomal protein S7 OS=Chlamydia trachomatis serovar L2b (strain UCH-1/proctitis) GN=rpsG PE=3 SV=1 | 153.2 | 17 | 2 | 2 | 17.8 |
| G4NNI2\|G4NNI2_CHLT4 | 30S ribosomal protein S7 OS=Chlamydia trachomatis serovar A (strain A2497) GN=rpsG PE=3 SV=1 | 153.2 | 17 | 2 | 2 | 17.8 |
| A0A0E9G3M0\|A0A0E9G3M0_CHLTH | Transporter OS=Chlamydia trachomatis GN=ERS066954_00215 PE=4 SV=1 | 143.0 | 9 | 4 | 4 | 77.1 |
| A0A0H3MH86\|A0A0H3MH86_CHLT2 | Uncharacterized protein OS=Chlamydia trachomatis serovar L2 (strain 434/Bu / ATCC VR-902B) GN=CTL0643 PE=4 SV=1 | 143.0 | 9 | 4 | 4 | 77.1 |
| G4NNG0\|G4NNG0_CHLT4 | 50S ribosomal protein L21 OS=Chlamydia trachomatis serovar A (strain A2497) GN=rplU PE=3 SV=1 | 151.5 | 45 | 4 | 4 | 12.3 |
| A0A0H3MB37\|A0A0H3MB37_CHLT2 | Cytochrome d ubiquinol oxidase subunit I OS=Chlamydia trachomatis serovar L2 (strain 434/Bu / ATCC VR-902B) GN=cydA PE=4 SV=1 | 91.2 | 4 | 2 | 2 | 50.2 |
| A0A0H2X0C7\|A0A0H2X0C7_CHLTA | Cytochrome d ubiquinol oxidase subunit I OS=Chlamydia trachomatis serovar A (strain ATCC VR-571B / DSM 19440 / HAR-13) GN=cydA PE=4 SV=1 | 91.2 | 4 | 2 | 2 | 50.2 |
| O84016\|O84016_CHLTR | Cytochrome Oxidase Subunit I OS=Chlamydia trachomatis (strain D/UW-3/Cx) GN=cydA PE=4 SV=1 | 91.2 | 4 | 2 | 2 | 50.2 |
| M9UM80\|M9UM80_CHLTH | Type III secretion system chaperone OS=Chlamydia trachomatis L2/434/Bu(f) GN=CTLFINAL_01575 PE=4 SV=1 | 121.9 | 20 | 2 | 2 | 18.4 |
| A0A0H3MBJ5\|A0A0H3MBJ5_CHLT2 | Putative type III secretion system chaperone OS=Chlamydia trachomatis serovar L2 (strain 434/Bu / ATCC VR-902B) GN=CTL0299 PE=4 SV=1 | 121.9 | 20 | 2 | 2 | 18.4 |
| A0A0H2X292\|A0A0H2X292_CHLTA | Anti-sigma F factor antagonist OS=Chlamydia trachomatis serovar A (strain ATCC VR-571B / DSM 19440 / HAR-13) GN=rsbV PE=4 SV=1 | 206.6 | 51 | 5 | 5 | 12.5 |
| M9UM43\|M9UM43_CHLTH | Anti-sigma factor antagonist OS=Chlamydia trachomatis L2/434/Bu(f) GN=CTLFINAL_03560 PE=3 SV=1 | 206.6 | 51 | 5 | 5 | 12.5 |
| O84431\|O84431_CHLTR | Anti-sigma factor antagonist OS=Chlamydia trachomatis (strain D/UW-3/Cx) GN=rsbV_1 PE=3 SV=1 | 206.6 | 51 | 5 | 5 | 12.5 |
| A0A0H2X182\|A0A0H2X182_CHLTA | Hypothetical membrane associated protein OS=Chlamydia trachomatis serovar A (strain ATCC VR-571B / DSM 19440 / HAR-13) GN=CTA_0002 PE=4 SV=1 | 122.7 | 40 | 2 | 2 | 9.8 |
| A0A0E9D6X4\|A0A0E9D6X4_CHLTH | Putative membrane associated protein OS=Chlamydia trachomatis GN=ERS075185_00609 PE=4 SV=1 | 122.7 | 40 | 2 | 2 | 9.8 |
| M9UKV6\|M9UKV6_CHLTH | Membrane protein OS=Chlamydia trachomatis L2/434/Bu(f) GN=CTLFINAL_01355 PE=4 SV=1 | 122.7 | 40 | 2 | 2 | 9.8 |
| G4NNU9\|G4NNU9_CHLT4 | Putative membrane associated protein OS=Chlamydia trachomatis serovar A (strain A2497) GN=CTO_0002 PE=4 SV=1 | 122.7 | 40 | 2 | 2 | 9.8 |
| sp\|O84007\|GATB_CHLTR | Aspartyl/glutamyl-tRNA(Asn/Gln) amidotransferase subunit B OS=Chlamydia trachomatis (strain D/UW-3/Cx) GN=gatB PE=3 SV=1 | 181.4 | 17 | 5 | 5 | 55.0 |
| M9UE49\|M9UE49_CHLTH | Aspartyl/glutamyl-tRNA(Asn/Gln) amidotransferase subunit B OS=Chlamydia trachomatis L2/434/Bu(f) GN=gatB PE=3 SV=1 | 181.4 | 17 | 5 | 5 | 55.0 |
| A0A0E9C8U7\|A0A0E9C8U7_CHLTH | Ribonuclease E OS=Chlamydia trachomatis GN=rng PE=4 SV=1 | 108.1 | 8 | 2 | 2 | 59.4 |
| A0A0H2X210\|A0A0H2X210_CHLTA | Ribonuclease E OS=Chlamydia trachomatis serovar A (strain ATCC VR-571B / DSM 19440 / HAR-13) GN=cafE PE=4 SV=1 | 108.1 | 8 | 2 | 2 | 59.3 |
| M9UKL6\|M9UKL6_CHLTH | Ribonuclease G OS=Chlamydia trachomatis L2/434/Bu(f) GN=CTLFINAL_00950 PE=4 SV=1 | 108.1 | 8 | 2 | 2 | 59.4 |
| A0A0H3MBC8\|A0A0H3MBC8_CHLT2 | Ribonuclease E OS=Chlamydia trachomatis serovar L2 (strain 434/Bu / ATCC VR-902B) GN=cafE PE=4 SV=1 | 108.1 | 8 | 2 | 2 | 59.4 |
| O84814\|O84814_CHLTR | Axial Filament Protein OS=Chlamydia trachomatis (strain D/UW-3/Cx) GN=cafE PE=4 SV=1 | 108.1 | 8 | 2 | 2 | 59.3 |
| A0A0E9DHL4\|A0A0E9DHL4_CHLTH | Ribonuclease E OS=Chlamydia trachomatis GN=rng PE=4 SV=1 | 108.1 | 8 | 2 | 2 | 59.3 |
| G4NPN0\|G4NPN0_CHLT4 | Ribonuclease E OS=Chlamydia trachomatis serovar A (strain A2497) GN=CTO_0880 PE=4 SV=1 | 108.1 | 7 | 2 | 2 | 60.9 |
| sp\|B0B7S2\|DEF_CHLT2 | Peptide deformylase OS=Chlamydia trachomatis serovar L2 (strain 434/Bu / ATCC VR-902B) GN=def PE=3 SV=1 | 177.1 | 30 | 5 | 5 | 20.5 |
| sp\|Q3KM05\|DEF_CHLTA | Peptide deformylase OS=Chlamydia trachomatis serovar A (strain ATCC VR-571B / DSM 19440 / HAR-13) GN=def PE=3 SV=1 | 177.1 | 30 | 5 | 5 | 20.5 |
| A0A0E9DI70\|A0A0E9DI70_CHLTH | 1-deoxy-D-xylulose 5-phosphate reductoisomerase OS=Chlamydia trachomatis GN=dxr PE=3 SV=1 | 85.1 | 6 | 2 | 2 | 41.8 |
| A0A0E9CXM5\|A0A0E9CXM5_CHLTH | 1-deoxy-D-xylulose 5-phosphate reductoisomerase OS=Chlamydia trachomatis GN=dxr PE=3 SV=1 | 85.1 | 6 | 2 | 2 | 41.7 |
| A0A0E9EDI2\|A0A0E9EDI2_CHLTH | Serine--tRNA ligase OS=Chlamydia trachomatis GN=serS_1 PE=3 SV=1 | 118.6 | 13 | 4 | 4 | 48.4 |
| sp\|Q3KKW5\|SYS_CHLTA | Serine--tRNA ligase OS=Chlamydia trachomatis serovar A (strain ATCC VR-571B / DSM 19440 / HAR-13) GN=serS PE=3 SV=1 | 118.6 | 13 | 4 | 4 | 48.3 |
| sp\|Q3KLH6\|RL16_CHLTA | 50S ribosomal protein L16 OS=Chlamydia trachomatis serovar A (strain ATCC VR-571B / DSM 19440 / HAR-13) GN=rplP PE=3 SV=1 | 148.6 | 20 | 2 | 2 | 15.8 |
| sp\|B0BCG0\|RL16_CHLTB | 50S ribosomal protein L16 OS=Chlamydia trachomatis serovar L2b (strain UCH-1/proctitis) GN=rplP PE=3 SV=1 | 148.6 | 20 | 2 | 2 | 15.8 |
| A0A0E9CFD6\|A0A0E9CFD6_CHLTH | 50S ribosomal protein L16 OS=Chlamydia trachomatis GN=rplP_1 PE=3 SV=1 | 148.6 | 20 | 2 | 2 | 15.8 |
| A0A0E9DS10\|A0A0E9DS10_CHLTH | ABC transporter ATPase OS=Chlamydia trachomatis GN=dppF PE=3 SV=1 | 134.2 | 19 | 4 | 2 | 31.0 |
| A0A0E9CMC9\|A0A0E9CMC9_CHLTH | Elongation factor P OS=Chlamydia trachomatis GN=efp-2 PE=3 SV=1 | 108.1 | 24 | 3 | 3 | 19.5 |
| A0A0E9DDN6\|A0A0E9DDN6_CHLTH | Elongation factor P OS=Chlamydia trachomatis GN=efp-2 PE=3 SV=1 | 108.1 | 23 | 3 | 3 | 20.5 |
| sp\|O84804\|GLGA_CHLTR | Glycogen synthase OS=Chlamydia trachomatis (strain D/UW-3/Cx) GN=glgA PE=3 SV=1 | 82.1 | 5 | 2 | 2 | 53.4 |
| A0A0E9CL94\|A0A0E9CL94_CHLTH | Glycogen synthase OS=Chlamydia trachomatis GN=glgA2 PE=3 SV=1 | 82.1 | 5 | 2 | 2 | 53.4 |
| O84412\|O84412_CHLTR | DnaK Suppressor OS=Chlamydia trachomatis (strain D/UW-3/Cx) GN=dksA PE=4 SV=1 | 129.8 | 43 | 4 | 4 | 13.9 |
| A0A0H3MCC6\|A0A0H3MCC6_CHLT2 | DnaK suppressor protein OS=Chlamydia trachomatis serovar L2 (strain 434/Bu / ATCC VR-902B) GN=dksA PE=4 SV=1 | 129.8 | 43 | 4 | 4 | 13.9 |
| A0A0E9FXT1\|A0A0E9FXT1_CHLTH | DnaK suppressor protein OS=Chlamydia trachomatis GN=yocK PE=4 SV=1 | 129.8 | 43 | 4 | 4 | 13.9 |
| M9UHY5\|M9UHY5_CHLTH | Molecular chaperone DnaK OS=Chlamydia trachomatis L2/434/Bu(f) GN=CTLFINAL_03465 PE=4 SV=2 | 129.8 | 39 | 4 | 4 | 15.3 |
| M9UFX8\|M9UFX8_CHLTH | Dihydrolipoyl dehydrogenase OS=Chlamydia trachomatis L2/434/Bu(f) GN=CTLFINAL_04275 PE=4 SV=1 | 105.3 | 5 | 2 | 2 | 49.5 |
| A0A0H2X2R2\|A0A0H2X2R2_CHLTA | Dihydrolipoamide dehydrogenase OS=Chlamydia trachomatis serovar A (strain ATCC VR-571B / DSM 19440 / HAR-13) GN=lpdA PE=4 SV=1 | 105.3 | 5 | 2 | 2 | 49.5 |
| A0A0E9CS98\|A0A0E9CS98_CHLTH | Dihydrolipoyl dehydrogenase OS=Chlamydia trachomatis GN=pdhD PE=4 SV=1 | 105.3 | 5 | 2 | 2 | 49.5 |
| A0A0H3MHJ2\|A0A0H3MHJ2_CHLT2 | Dihydrolipoamide dehydrogenase OS=Chlamydia trachomatis serovar L2 (strain 434/Bu / ATCC VR-902B) GN=lpdA PE=4 SV=1 | 105.3 | 5 | 2 | 2 | 49.5 |
| A0A0E9CCI4\|A0A0E9CCI4_CHLTH | Dihydrolipoyl dehydrogenase OS=Chlamydia trachomatis GN=pdhD PE=4 SV=1 | 105.3 | 5 | 2 | 2 | 49.5 |
| A0A0E9DGP2\|A0A0E9DGP2_CHLTH | Pyruvate dehydrogenase%2C E1 component%2C beta subunit OS=Chlamydia trachomatis GN=pdhB PE=4 SV=1 | 84.4 | 8 | 2 | 2 | 36.2 |
| A0A0H3MBQ9\|A0A0H3MBQ9_CHLT2 | Putative exported protein OS=Chlamydia trachomatis serovar L2 (strain 434/Bu / ATCC VR-902B) GN=CTL0473 PE=4 SV=1 | 80.1 | 6 | 2 | 2 | 33.1 |
| A0A0E9DL21\|A0A0E9DL21_CHLTH | Putative membrane spanning protein OS=Chlamydia trachomatis GN=ERS066953_00163 PE=4 SV=1 | 80.1 | 6 | 2 | 2 | 33.1 |
| sp\|Q3KKY8\|CLPP2_CHLTA | ATP-dependent Clp protease proteolytic subunit 2 OS=Chlamydia trachomatis serovar A (strain ATCC VR-571B / DSM 19440 / HAR-13) GN=clpP2 PE=3 SV=1 | 108.1 | 26 | 4 | 4 | 22.0 |
| M9ULH7\|M9ULH7_CHLTH | ATP-dependent Clp protease proteolytic subunit OS=Chlamydia trachomatis L2/434/Bu(f) GN=clpP PE=3 SV=1 | 108.1 | 26 | 4 | 4 | 22.0 |
| A0A0E9FWY4\|A0A0E9FWY4_CHLTH | MotA/TolQ/ExbB proton channel family protein OS=Chlamydia trachomatis GN=exbB PE=3 SV=1 | 107.2 | 14 | 2 | 2 | 25.9 |
| M9UI72\|M9UI72_CHLTH | Oxidoreductase OS=Chlamydia trachomatis L2/434/Bu(f) GN=CTLFINAL_03620 PE=4 SV=1 | 102.1 | 5 | 2 | 2 | 39.9 |
| A0A0H2X2I2\|A0A0H2X2I2_CHLTA | NADPH-cytochrome P450 reductase OS=Chlamydia trachomatis serovar A (strain ATCC VR-571B / DSM 19440 / HAR-13) GN=cysJ PE=4 SV=1 | 102.1 | 5 | 2 | 2 | 39.9 |
| G4NNH9\|G4NNH9_CHLT4 | NADPH-cytochrome P450 reductase OS=Chlamydia trachomatis serovar A (strain A2497) GN=CTO_0475 PE=4 SV=1 | 102.1 | 5 | 2 | 2 | 39.9 |
| O84442\|O84442_CHLTR | Sulfite Reductase OS=Chlamydia trachomatis (strain D/UW-3/Cx) GN=cysJ PE=4 SV=1 | 102.1 | 5 | 2 | 2 | 39.9 |
| A0A0E9DWF3\|A0A0E9DWF3_CHLTH | Pyruvate dehydrogenase E1 component subunit alpha OS=Chlamydia trachomatis GN=pdhA_1 PE=4 SV=1 | 86.2 | 8 | 2 | 2 | 37.2 |
| G4NP68\|G4NP68_CHLT4 | Pyruvate dehydrogenase E1 component subunit alpha OS=Chlamydia trachomatis serovar A (strain A2497) GN=pdhA PE=4 SV=1 | 86.2 | 8 | 2 | 2 | 37.2 |
| A0A0H3MGN2\|A0A0H3MGN2_CHLT2 | ADP ATP carrier protein OS=Chlamydia trachomatis serovar L2 (strain 434/Bu / ATCC VR-902B) GN=CTL0321 PE=4 SV=1 | 84.6 | 4 | 2 | 2 | 58.1 |
| M9UEC9\|M9UEC9_CHLTH | ATPase AAA OS=Chlamydia trachomatis L2/434/Bu(f) GN=CTLFINAL_01695 PE=4 SV=1 | 84.6 | 4 | 2 | 2 | 58.1 |
| sp\|O84068\|TLC1_CHLTR | ADP ATP carrier protein 1 OS=Chlamydia trachomatis (strain D/UW-3/Cx) GN=tlcA PE=3 SV=1 | 84.6 | 4 | 2 | 2 | 58.1 |
| A0A0E9DLH1\|A0A0E9DLH1_CHLTH | ADP%2C ATP carrier protein OS=Chlamydia trachomatis GN=tlc-1 PE=4 SV=1 | 84.6 | 4 | 2 | 2 | 58.1 |
| sp\|P66123\|RL27_CHLTR | 50S ribosomal protein L27 OS=Chlamydia trachomatis (strain D/UW-3/Cx) GN=rpmA PE=3 SV=1 | 90.3 | 33 | 2 | 2 | 8.9 |
| A0A0E9C7I7\|A0A0E9C7I7_CHLTH | 50S ribosomal protein L27 OS=Chlamydia trachomatis GN=rpmA PE=3 SV=1 | 90.3 | 33 | 2 | 2 | 8.9 |
| G4NNF9\|G4NNF9_CHLT4 | 50S ribosomal protein L27 OS=Chlamydia trachomatis serovar A (strain A2497) GN=rpmA PE=3 SV=1 | 90.3 | 33 | 2 | 2 | 8.9 |
| sp\|Q3KLT4\|RL27_CHLTA | 50S ribosomal protein L27 OS=Chlamydia trachomatis serovar A (strain ATCC VR-571B / DSM 19440 / HAR-13) GN=rpmA PE=3 SV=1 | 90.3 | 33 | 2 | 2 | 8.9 |
| A0A0E9D620\|A0A0E9D620_CHLTH | Cysteine--tRNA ligase OS=Chlamydia trachomatis GN=cysS PE=3 SV=1 | 86.7 | 4 | 2 | 2 | 57.2 |
| A0A0H2X1S7\|A0A0H2X1S7_CHLTA | HAD superfamily hydrolase/phosphatase OS=Chlamydia trachomatis serovar A (strain ATCC VR-571B / DSM 19440 / HAR-13) GN=CTA_0109 PE=4 SV=1 | 152.9 | 19 | 4 | 4 | 34.3 |
| A0A0E9CFX5\|A0A0E9CFX5_CHLTH | HAD superfamily hydrolase/phosphatase OS=Chlamydia trachomatis GN=ERS075185_00712 PE=4 SV=1 | 152.9 | 19 | 4 | 4 | 34.3 |
| M9UHS2\|M9UHS2_CHLTH | Deoxyuridine 5'-triphosphate nucleotidohydrolase OS=Chlamydia trachomatis L2/434/Bu(f) GN=dut PE=3 SV=1 | 127.5 | 37 | 3 | 3 | 15.3 |
| A0A0E9DSY4\|A0A0E9DSY4_CHLTH | Deoxyuridine 5'-triphosphate nucleotidohydrolase OS=Chlamydia trachomatis GN=dut_2 PE=3 SV=1 | 127.5 | 37 | 3 | 3 | 15.3 |
| sp\|B0BBS3\|DUT_CHLTB | Deoxyuridine 5'-triphosphate nucleotidohydrolase OS=Chlamydia trachomatis serovar L2b (strain UCH-1/proctitis) GN=dut PE=3 SV=1 | 127.5 | 37 | 3 | 3 | 15.3 |
| O84776\|O84776_CHLTR | Hydrolase/phosphatase homolog OS=Chlamydia trachomatis (strain D/UW-3/Cx) GN=CT_771 PE=3 SV=1 | 114.0 | 25 | 3 | 3 | 17.4 |
| A0A0H2X385\|A0A0H2X385_CHLTA | Phosphohydrolase (MutT/nudix family protein) OS=Chlamydia trachomatis serovar A (strain ATCC VR-571B / DSM 19440 / HAR-13) GN=CTA_0841 PE=4 SV=1 | 114.0 | 25 | 3 | 3 | 17.4 |
| G4NPJ0\|G4NPJ0_CHLT4 | Phosphohydrolase (MutT/nudix family protein) OS=Chlamydia trachomatis serovar A (strain A2497) GN=CTO_0841 PE=4 SV=1 | 114.0 | 25 | 3 | 3 | 17.4 |
| A0A0E9DK93\|A0A0E9DK93_CHLTH | MutT/Nudix family protein OS=Chlamydia trachomatis GN=ERS075193_00895 PE=3 SV=1 | 114.0 | 25 | 3 | 3 | 17.4 |
| sp\|B0B9I6\|FOLD_CHLT2 | Bifunctional protein FolD OS=Chlamydia trachomatis serovar L2 (strain 434/Bu / ATCC VR-902B) GN=folD PE=3 SV=1 | 101.2 | 11 | 2 | 2 | 30.9 |
| sp\|B0BB65\|FOLD_CHLTB | Bifunctional protein FolD OS=Chlamydia trachomatis serovar L2b (strain UCH-1/proctitis) GN=folD PE=3 SV=1 | 101.2 | 11 | 2 | 2 | 30.9 |
| M9UI50\|M9UI50_CHLTH | Metallophosphoesterase OS=Chlamydia trachomatis L2/434/Bu(f) GN=CTLFINAL_03765 PE=4 SV=1 | 110.8 | 14 | 3 | 3 | 37.1 |
| sp\|Q3KMZ0\|SYM_CHLTA | Methionine--tRNA ligase OS=Chlamydia trachomatis serovar A (strain ATCC VR-571B / DSM 19440 / HAR-13) GN=metG PE=3 SV=1 | 113.6 | 6 | 3 | 3 | 62.7 |
| A0A0E9DB98\|A0A0E9DB98_CHLTH | Methionine--tRNA ligase OS=Chlamydia trachomatis GN=metG_1 PE=3 SV=1 | 113.6 | 6 | 3 | 3 | 62.7 |
| A0A0E9CW55\|A0A0E9CW55_CHLTH | Peptidoglycan associated lipoprotein OS=Chlamydia trachomatis GN=pal PE=3 SV=1 | 91.7 | 11 | 2 | 2 | 21.5 |
| M9UNW8\|M9UNW8_CHLTH | Membrane protein OS=Chlamydia trachomatis L2/434/Bu(f) GN=CTLFINAL_04505 PE=3 SV=1 | 91.7 | 11 | 2 | 2 | 21.5 |
| A0A0H2X2P6\|A0A0H2X2P6_CHLTA | Peptidoglycan-associated lipoprotein OS=Chlamydia trachomatis serovar A (strain ATCC VR-571B / DSM 19440 / HAR-13) GN=pal PE=4 SV=1 | 91.7 | 11 | 2 | 2 | 21.5 |
| A0A0H3MDU9\|A0A0H3MDU9_CHLT2 | Peptidoglycan-associated lipoprotein OS=Chlamydia trachomatis serovar L2 (strain 434/Bu / ATCC VR-902B) GN=pal PE=4 SV=1 | 91.7 | 11 | 2 | 2 | 21.5 |
| sp\|O84586\|PARA_CHLTR | ParA family protein CT_582 OS=Chlamydia trachomatis (strain D/UW-3/Cx) GN=CT_582 PE=3 SV=1 | 97.7 | 24 | 4 | 4 | 28.2 |
| A0A0H3MDT7\|A0A0H3MDT7_CHLT2 | Chromosome partitioning ATPase (ParA family) OS=Chlamydia trachomatis serovar L2 (strain 434/Bu / ATCC VR-902B) GN=minD PE=4 SV=1 | 97.7 | 24 | 4 | 4 | 28.2 |
| A0A0E9DFM9\|A0A0E9DFM9_CHLTH | ATPase%2C ParA FAMILY OS=Chlamydia trachomatis GN=soj_1 PE=4 SV=1 | 97.7 | 24 | 4 | 4 | 28.2 |
| A0A0E9D3V4\|A0A0E9D3V4_CHLTH | ATPase%2C ParA FAMILY OS=Chlamydia trachomatis GN=soj_1 PE=4 SV=1 | 97.7 | 24 | 4 | 4 | 28.2 |
| M9UMM8\|M9UMM8_CHLTH | Chromosome partitioning protein ParA OS=Chlamydia trachomatis L2/434/Bu(f) GN=CTLFINAL_04410 PE=4 SV=1 | 97.7 | 24 | 4 | 4 | 28.2 |
| G4NMM3\|G4NMM3_CHLT4 | ATPase ParA FAMILY OS=Chlamydia trachomatis serovar A (strain A2497) GN=CTO_0632 PE=4 SV=1 | 97.7 | 24 | 4 | 4 | 28.6 |
| A0A0E9DV71\|A0A0E9DV71_CHLTH | ATPase%2C ParA FAMILY OS=Chlamydia trachomatis GN=soj_4 PE=4 SV=1 | 97.7 | 24 | 4 | 4 | 28.6 |
| G4NPH0\|G4NPH0_CHLT4 | AMP nucleosidase OS=Chlamydia trachomatis serovar A (strain A2497) GN=CTO_0820 PE=4 SV=1 | 78.4 | 9 | 2 | 2 | 32.0 |
| A0A0H2X1U4\|A0A0H2X1U4_CHLTA | AMP nucleosidase OS=Chlamydia trachomatis serovar A (strain ATCC VR-571B / DSM 19440 / HAR-13) GN=amn PE=4 SV=1 | 78.4 | 9 | 2 | 2 | 32.0 |
| A0A0E9D1J8\|A0A0E9D1J8_CHLTH | Protein RsbW OS=Chlamydia trachomatis GN=btrW PE=4 SV=1 | 107.7 | 23 | 3 | 3 | 16.4 |
| O84553\|O84553_CHLTR | Sigma regulatory factor-histidine kinase OS=Chlamydia trachomatis (strain D/UW-3/Cx) GN=rsbW PE=4 SV=1 | 107.7 | 23 | 3 | 3 | 16.4 |
| A0A0E9CFX6\|A0A0E9CFX6_CHLTH | Protein RsbW OS=Chlamydia trachomatis GN=btrW PE=4 SV=1 | 107.7 | 23 | 3 | 3 | 16.4 |
| M9UMJ4\|M9UMJ4_CHLTH | Histidine kinase OS=Chlamydia trachomatis L2/434/Bu(f) GN=CTLFINAL_04235 PE=4 SV=1 | 107.7 | 23 | 3 | 3 | 16.4 |
| A0A0H3MLE8\|A0A0H3MLE8_CHLT2 | Sigma regulatory factor-histidine kinase OS=Chlamydia trachomatis serovar L2 (strain 434/Bu / ATCC VR-902B) GN=rsbW PE=4 SV=1 | 107.7 | 23 | 3 | 3 | 16.4 |
| G4NPG9\|G4NPG9_CHLT4 | RsbW OS=Chlamydia trachomatis serovar A (strain A2497) GN=CTO_0599 PE=4 SV=1 | 107.7 | 22 | 3 | 3 | 17.3 |
| A0A0E9D4E7\|A0A0E9D4E7_CHLTH | Glutamate--tRNA ligase OS=Chlamydia trachomatis GN=gltX PE=3 SV=1 | 112.9 | 4 | 2 | 2 | 58.6 |
| G4NNJ1\|G4NNJ1_CHLT4 | Glutamate--tRNA ligase OS=Chlamydia trachomatis serovar A (strain A2497) GN=gltX PE=3 SV=1 | 112.9 | 4 | 2 | 2 | 58.5 |
| A0A0H3MGR8\|A0A0H3MGR8_CHLT2 | Biotin carboxyl carrier protein of acetyl-CoA carboxylase OS=Chlamydia trachomatis serovar L2 (strain 434/Bu / ATCC VR-902B) GN=accB PE=4 SV=1 | 107.6 | 20 | 2 | 2 | 18.2 |
| A0A0E9D783\|A0A0E9D783_CHLTH | Acetyl-CoA carboxylase biotin carboxyl carrier protein subunit OS=Chlamydia trachomatis GN=accB PE=4 SV=1 | 107.6 | 20 | 2 | 2 | 18.2 |
| G4NPJ4\|G4NPJ4_CHLT4 | Acyl-acyl carrier protein synthetase OS=Chlamydia trachomatis serovar A (strain A2497) GN=CTO_0846 PE=4 SV=1 | 76.7 | 6 | 2 | 2 | 59.4 |
| A0A0H2X0W7\|A0A0H2X0W7_CHLTA | Hypothetical secreted protein OS=Chlamydia trachomatis serovar A (strain ATCC VR-571B / DSM 19440 / HAR-13) GN=CTA_0275 PE=4 SV=1 | 74.7 | 12 | 2 | 2 | 24.0 |
| G4NP76\|G4NP76_CHLT4 | Putative secreted protein OS=Chlamydia trachomatis serovar A (strain A2497) GN=CTO_0275 PE=4 SV=1 | 74.7 | 12 | 2 | 2 | 24.0 |
| A0A0E9CQK7\|A0A0E9CQK7_CHLTH | Uncharacterized conserved protein OS=Chlamydia trachomatis GN=ERS075194_01595 PE=4 SV=1 | 90.9 | 26 | 3 | 3 | 18.3 |
| sp\|B0B929\|RS18_CHLT2 | 30S ribosomal protein S18 OS=Chlamydia trachomatis serovar L2 (strain 434/Bu / ATCC VR-902B) GN=rpsR PE=3 SV=1 | 97.2 | 27 | 3 | 3 | 9.4 |
| sp\|Q3KKN9\|RS18_CHLTA | 30S ribosomal protein S18 OS=Chlamydia trachomatis serovar A (strain ATCC VR-571B / DSM 19440 / HAR-13) GN=rpsR PE=3 SV=1 | 97.2 | 27 | 3 | 3 | 9.4 |
| A0A0E9DK62\|A0A0E9DK62_CHLTH | 30S ribosomal protein S18 OS=Chlamydia trachomatis GN=rpsR PE=3 SV=1 | 97.2 | 27 | 3 | 3 | 9.4 |
| A0A0E9DSX9\|A0A0E9DSX9_CHLTH | Thymidylate kinase OS=Chlamydia trachomatis GN=tmk_2 PE=3 SV=1 | 83.9 | 17 | 2 | 2 | 22.5 |
| sp\|B0B9T8\|KTHY_CHLT2 | Thymidylate kinase OS=Chlamydia trachomatis serovar L2 (strain 434/Bu / ATCC VR-902B) GN=tmk PE=3 SV=1 | 83.9 | 17 | 2 | 2 | 22.5 |
| A0A0H2X1D2\|A0A0H2X1D2_CHLTA | Methyltransferase OS=Chlamydia trachomatis serovar A (strain ATCC VR-571B / DSM 19440 / HAR-13) GN=yhhF PE=4 SV=1 | 140.8 | 18 | 2 | 2 | 20.9 |
| O84494\|O84494_CHLTR | Methylase OS=Chlamydia trachomatis (strain D/UW-3/Cx) GN=yhhF PE=4 SV=1 | 140.8 | 18 | 2 | 2 | 20.9 |
| A0A0H3MDP4\|A0A0H3MDP4_CHLT2 | Methyltransferase OS=Chlamydia trachomatis serovar L2 (strain 434/Bu / ATCC VR-902B) GN=CTL0748 PE=4 SV=1 | 140.8 | 18 | 2 | 2 | 20.9 |
| G4NPI2\|G4NPI2_CHLT4 | RNA binding protein OS=Chlamydia trachomatis serovar A (strain A2497) GN=CTO_0833 PE=3 SV=1 | 92.1 | 24 | 3 | 3 | 15.5 |
| A0A0E9DER9\|A0A0E9DER9_CHLTH | Chlamydia protein associating with death domains OS=Chlamydia trachomatis GN=ERS075193_00154 PE=4 SV=1 | 78.5 | 15 | 2 | 2 | 26.1 |
| M9UIP8\|M9UIP8_CHLTH | Dehydrogenase OS=Chlamydia trachomatis L2/434/Bu(f) GN=CTLFINAL_04565 PE=4 SV=1 | 78.5 | 15 | 2 | 2 | 26.8 |
| sp\|O84616\|PQQCL_CHLTR | PqqC-like protein OS=Chlamydia trachomatis (strain D/UW-3/Cx) GN=CT_610 PE=1 SV=1 | 78.5 | 15 | 2 | 2 | 26.7 |
| G4NMQ5\|G4NMQ5_CHLT4 | Chlamydia protein associating with death domains OS=Chlamydia trachomatis serovar A (strain A2497) GN=CTO_0663 PE=4 SV=1 | 78.5 | 14 | 2 | 2 | 27.5 |
